# Frequency and prognostic value of mutations associated with the homologous recombination DNA repair pathway in a large pan cancer cohort

**DOI:** 10.1038/s41598-020-76975-6

**Published:** 2020-11-19

**Authors:** Daniel R. Principe, Matthew Narbutis, Regina Koch, Ajay Rana

**Affiliations:** 1 Division of Surgical Oncology, Department of Surgery, College of Medicine, The University of Illinois at Chicago, 840 S. Wood Street, Suite 601 Clinical Sciences Building, Chicago, IL 60612 USA; 2 Medical Scientist Training Program, University of Illinois College of Medicine, Chicago, IL USA; 3 University of Illinois College of Medicine, Chicago, IL USA; 4 Jesse Brown VA Medical Center, Chicago, IL USA

**Keywords:** Tumour biomarkers, Cancer, Cancer genetics

## Abstract

PARP inhibitors have shown remarkable efficacy in the clinical management of several *BRCA*-mutated tumors. This approach is based on the long-standing hypothesis that PARP inhibition will impair the repair of single stranded breaks, causing synthetic lethality in tumors with loss of high-fidelity double-strand break homologous recombination. While this is now well accepted and has been the basis of several successful clinical trials, emerging evidence strongly suggests that mutation to several additional genes involved in homologous recombination may also have predictive value for PARP inhibitors. While this notion is supported by early clinical evidence, the mutation frequencies of these and other functionally related genes are largely unknown, particularly in cancers not classically associated with homologous recombination deficiency. We therefore evaluated the mutation status of 22 genes associated with the homologous recombination DNA repair pathway or PARP inhibitor sensitivity, first in a pan-cancer cohort of 55,586 patients, followed by a more focused analysis in The Cancer Genome Atlas cohort of 12,153 patients. In both groups we observed high rates of mutations in a variety of HR-associated genes largely unexplored in the setting of PARP inhibition, many of which were associated also with poor clinical outcomes. We then extended our study to determine which mutations have a known oncogenic role, as well as similar to known oncogenic mutations that may have a similar phenotype. Finally, we explored the individual cancer histologies in which these genomic alterations are most frequent. We concluded that the rates of deleterious mutations affecting genes associated with the homologous recombination pathway may be underrepresented in a wide range of human cancers, and several of these genes warrant further and more focused investigation, particularly in the setting of PARP inhibition and HR deficiency.

## Introduction

Precision medicine has become standard of care in the management of several malignancies. This approach involves the identification of clinically actionable molecular features, typically via Next Generation Sequencing (NGS), and the subsequent implementation of a specific, targeted therapy. For example, Tyrosine kinase inhibitors such as imatinib, bosutinib, and dasatinib targeting the BCR-ABL fusion protein have improved outcomes in Philadelphia chromosome positive leukemia^1^, and similar approaches have made a considerable impact in breast cancer^2^, non-small cell lung cancer (NSCLC)^3^, and several other cancer types. This approach has significantly improved outcomes in a variety of tumor types, as a recent pan-cancer trial of 1144 patients determined that those harboring distinct molecular aberrations and treated with a matched targeted therapy had significant improvements in overall response rates, time-to-treatment failure, and overall survival^4^.

There is a large body of evidence strongly supporting the use of selective PARP inhibitors such as olaparib or talazoparib in *BRCA*-mutated tumors^5^. This approach has strong scientific rationale, as patients with *BRCA* mutations are thought to have homologous recombination deficiency (HRD), thereby limiting their ability to repair double stranded DNA breaks. The use of PARP inhibitors in these patients limits their ability to undergo single stranded break repair, leading to the accumulation of DNA damage and eventually cell death^6,7^. This approach has shown clinical efficacy in the management of *BRCA*-mutated breast, ovarian, pancreas, and prostate cancers^8–11^, and more recently glioblastoma multiforme and metastatic thymomas^12,13^.

While *BRCA* is a strong predictor for the efficacy of PARP inhibitors, homologous recombination (HR) involves a wide range of additional DNA repair genes, some of which may also have predictive value for PARP inhibitors. For instance, in metastatic prostate cancer, mutations to genes more modestly associated with the pathway such as *ATM*, *CHEK2*, and *PALB2* are strongly associated with clinical responses to olaparib^14,15^. For example, *ATM*-deficient cell lines have shown increased sensitivity to PARP inhibition than their *ATM*-proficient counterparts in variety of cancer types^16–20^. Likewise, 88% of prostate cancer patients with *CHECK2* mutations showed clinical responses to PARP inhibition^14^, with similar results observed in other studies, many including additional cancer histologies^21–24^. Similarly, *PALB2* mutated breast cancer also appears to be highly sensitive to PARP inhibition^25^. This appears to extend to genes that are far up or downstream of the HR pathway, as *PTEN* deficient tumors have been suggested to respond to PARP inhibitors due to loss of *RAD51*, though this remains unclear and *PTEN* is not currently considered a useful predictor for PARP inhibition^26–29^. However, it is clear that there are several additional HR associated mutations that may also be informative when stratifying patients for PARP inhibition.

While several studies have explored the mutation frequency and predictive value of established HR associated genes such as *BRCA1/2* or upstream HR-associated genes *ATM*, *CHEK2*, and *PALB2*, few have evaluated alterations to other functionally related HR genes. This is particularly true for genes more weakly associated with HRD, some of which are beginning to show predictive value for PARP inhibition^30^. Such genes include *BARD1*, *BRIP1*, *FAAP20*, *FAN1*, *FANCE*, *FANCM*, *RAD51B*, *RAD51C*, and *RAD51D,* all of which have been suggested to predict for PARP inhibitor sensitivity^31–33^, with additional context specific roles emerging for genes such as *POLQ*. For instance, though loss of *POLQ* appears to upregulate HR activity in HR-proficient cells, loss of *POLQ* is also seemingly central to PARP inhibitor sensitivity in the setting of topoisomerase, ATR, or *FANCD2*-deficiency^34,35^.

Hence, it is clear that stratifying patients based solely BRCA mutations will likely under predict for those who will derive clinical benefit from PARP inhibition. We therefore evaluated the mutation status of 22 genes with either established, emerging, or potential roles in either the HR repair pathway or PARP inhibitor sensitivity, first in a pan cancer analysis of over 55,000 patients compiled from several genomic databases, followed by a more focused analysis of The Cancer Genome Atlas (TCGA) cohort, which allowed for more insight into disease-specific mutation frequencies. Interestingly, we observed high rates of mutations in a variety of largely unexplored HR genes, many of which were associated with poor clinical outcomes. We then identified the individual cancer types in which these alterations are most frequent. Though many of the observed mutations are currently of unknown significance, these newly identified genomic alterations warrant further investigation, particularly in the setting of homologous recombination deficiency and PARP inhibition.

## Methods

### Pan-cancer genomic database analysis

Patient data was visualized using cBioportal for Cancer Genomics as described in the original references^36,37^, and DNA/RNA sequencing analyses and protocols can be found per the references listed above. Using this dataset, survival was assessed using the Kaplan Meier method. Subsequent genetic analyses were restricted to fully sequenced tumors and gene sequences compared to a reference population as described previously^38^. A complete list of studies included in this analysis is listed in the supplemental materials section.

### TCGA database analysis

Provisional TCGA patient datasets were downloaded (https://tcga-data.nci.nih.gov/tcga/) and visualized using cBioportal for Cancer Genomics as described. Detailed information regarding the TCGA dataset and DNA sequencing analyses and protocols can be found on the TCGA data portal webpage listed above. Like the pan-cancer dataset, survival was assessed using the Kaplan Meier method, and subsequent genetic analyses were restricted to fully sequenced tumors also as described previously^38^.

#### List of studies included in TCGA analysis

Data from each of the following studies was compiled and visualized as described above: Pan-Lung Cancer (TCGA, Nat Genet 2016), Adrenocortical Carcinoma (TCGA, Provisional), Cholangiocarcinoma (TCGA, Provisional), Bladder Urothelial Carcinoma (TCGA, Provisional), Colorectal Adenocarcinoma (TCGA, Provisional), Breast Invasive Carcinoma (TCGA, Provisional), Brain Lower Grade Glioma (TCGA, Provisional), Merged Cohort of LGG and GBM (TCGA, Cell 2016), Glioblastoma Multiforme (TCGA, Provisional), Cervical Squamous Cell Carcinoma and Endocervical Adenocarcinoma (TCGA, Provisional), TCGA data for Esophagus-Stomach Cancers (TCGA, Nature 2017), Esophageal Carcinoma (TCGA, Provisional), Stomach Adenocarcinoma (TCGA, Provisional), Uveal Melanoma (TCGA, Provisional), Head and Neck Squamous Cell Carcinoma (TCGA, Provisional), Kidney Renal Clear Cell Carcinoma (TCGA, Provisional), Kidney Chromophobe (TCGA, Provisional), Kidney Renal Papillary Cell Carcinoma (TCGA, Provisional), Liver Hepatocellular Carcinoma (TCGA, Provisional), Lung Adenocarcinoma (TCGA, Provisional), Lung Squamous Cell Carcinoma (TCGA, Provisional), Lymphoid Neoplasm Diffuse Large B-cell Lymphoma (TCGA, Provisional), Acute Myeloid Leukemia (TCGA, Provisional), Ovarian Serous Cystadenocarcinoma (TCGA, Provisional), Pancreatic Adenocarcinoma (TCGA, Provisional), Pancreatic Adenocarcinoma (ICGC, Nature 2012), Mesothelioma (TCGA, Provisional), Prostate Adenocarcinoma (TCGA, Provisional), Skin Cutaneous Melanoma (TCGA, Provisional), Pheochromocytoma and Paraganglioma (TCGA, PanCancer Atlas), Sarcoma (TCGA, Provisional), Testicular Germ Cell Cancer (TCGA, Provisional), Thymoma (TCGA, Provisional), Thyroid Carcinoma (TCGA, Provisional), Uterine Carcinosarcoma (TCGA, Provisional), Uterine Corpus Endometrial Carcinoma (TCGA, Provisional).

### Inclusion/exclusion criteria

All genomic analyses were restricted to fully sequenced tumors. All studies listed were included in pan-cancer survival analyses, though mutation frequencies were limited to samples with an N ≥ 25.

### Statistical analysis

Data were analyzed by either student’s T test, Xi squared test, or ANOVA fit to a general linear model in Minitab express, the validity of which was tested by adherence to the normality assumption and the fitted plot of the residuals. Results were considered significant at either p or q < 0.05 unless otherwise noted.

## Results

### Mutations to genes associated with the homologous recombination pathway predict for poor clinical outcomes in a large pan-cancer study

To determine the frequency of pathway mutations in a large sample size, we first evaluated the mutation status of 22 key homologous recombination genes in a pooled pan-cancer cohort of 55,586 patients from 32 different cancer types (individual studies detailed in the supplemental methods). These genes include: *ATM*, *BARD1*, *BRCA1*, *BRCA2*, *BRIP1*, *CDK12*, *CHEK2*, *DMC1*, *FAAP20*, *FAN1*, *FANCD2*, *FANCE*, *FANCL*, *FANCM*, *PALB2*, *POLQ*, *RAD51*, *RAD51B*, *RAD51C*, *RAD51D*, *RAD54L*, and *XRCC3*. HR pathway mutations were common in this cohort, affecting 7117 (13.4%) of patients. *ATM* and *BRCA2* mutations were most common, affecting 2160 (4.1%) and 1452 (2.7%) of patients respectively, followed by *BRCA1* (822 or 1.5%), *CDK12* (805 or 1.5%), and *POLQ* (634 or 1.19%). These mutation frequencies are summarized in Table 1.

**Table 1:** Mutation frequencies of genes associated with the homologous recombination DNA repair pathway in a pan cancer cohort (N = 55,586).

Gene	Observed mutations pan cancer (N = 55,586)	Mutation frequency (%)
ATM	2160	4.056
BRCA2	1452	2.727
BRCA1	822	1.544
CDK12	805	1.512
POLQ	634	1.191
BRIP1	573	1.076
PALB2	507	0.952
FANCM	494	0.928
CHEK2	479	0.899
BARD1	429	0.806
FANCD2	377	0.708
RAD54L	235	0.441
FAN1	189	0.355
RAD51C	166	0.312
FANCE	134	0.252
RAD51B	150	0.282
RAD51D	112	0.210
RAD51	109	0.205
DMC1	84	0.158
FANCL	70	0.131
FAAP20	39	0.073
XRCC3	34	0.064
Any HR mutation	7117	13.365

**Figure 1:**
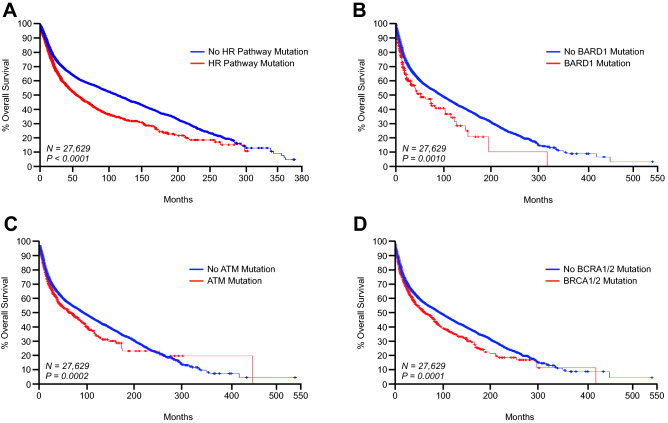
Mutations to genes associated with the homologous recombination pathway predict for poor clinical outcomes in a large pan-cancer study. We determined the mutation status of *ATM*, *BARD1*, *BRCA1*, *BRCA2*, *BRIP1*, *CDK12*, *CHEK2*, *DMC1*, *FAAP20*, *FAN1*, *FANCD2*, *FANCE*, *FANCL*, *FANCM*, *PALB2*, *POLQ*, *RAD51*, *RAD51B*, *RAD51C*, *RAD51D*, *RAD54L*, and *XRCC3* in a in a pooled pan-cancer cohort of 55,586 patients from 32 different cancer types. Of these patients, survival data was available from 27,629, which were used for subsequent analyses. Kaplan Meier plots are displayed showing overall survival from patients with or without a mutation to: (**A**) one or more of the genes listed above, (**B**) *BARD1*, (**C**) *ATM*, or (**D**) *BRCA1* and/or *BRCA2*.

Of the initial 55,586 patients, survival data was available for 33,633 (60.5%). Of these 33,633 patients, 4472 (13.3%) had an identifiable mutation to the queried HR genes, whereas 29,161 (86.7%) did not. Additionally, patients with any HR pathway mutation had significantly poorer outcomes, with a median overall survival of 60.5 months compared to the 105.91 months in patients with no HR pathway mutation (Fig. 1, Table 2). Interestingly, several HR genes were independent predictors of poor outcomes including *ATM*, *BARD1*, *BRCA2*, *CDK12*, *DMC1*, *FAAP20*, *PALB2*, and *POLQ*, though it is important to note that it is unlikely that these patients were treated with a PARP inhibitor (Fig. 1, Table 2).

**Table 2:** Select mutations to genes associated with the homologous recombination DNA repair pathway are associated with poor survival in a pan cancer cohort (N = 33,633).

Gene	Median months survival without mutation	Median months survival with mutation	P value
All genes	105.91	60.50	P < 0.0001
ATM	99.97	71.68	P = 1.610 × 10^–4^
BARD1	98.80	53.77	P = 1.34 × 10^–4^
BRCA1	99.00	82.63	P = 0.203
BRAC2	99.90	73.16	P = 1.577 × 10^–4^
BRCA1/2	100.62	73.16	P = 1.792 × 10^–5^
BRIP1	98.80	80.00	P = 0.139
CHEK2	86.37	98.5	P = 0.872
CDK12	99.40	53.15	P = 5.195 × 10^–3^
DMC1	98.77	50.3	P = 5.579 × 10^–3^
FAAP20	98.70	37.71	P = 0.0136
FAN1	98.70	76.97	P = 0.833
FANCD2	98.70	74.00	P = 0.606
FANCE	98.32	–	–
FANCL	98.50	109/00	P = 0.885
FANCM	98.83	57.59	P = 0.0567
PALB2	98.90	50.72	P = 0.0114
POLQ	99.53	63.50	P = 1.141 × 10^–3^
RAD51	98.50	72.01	P = 0.809
RAD51B	98.370	156.9	P = 0.549
RAD51C	98.70	42.00	P = 0.137
RAD51D	98.70	75.43	P = 0.121
RAD54L	98.50	77.00	P = 0.367
XRCC3	98.37	109.00	P = 0.295

### Mutations to genes associated with the homologous recombination pathway similarly predict poor clinical outcomes in The Cancer Genome Atlas cohorts

While these data suggest that as a whole, HR pathway mutations may have prognostic value, these results may be skewed should HR mutations be more frequently observed in more aggressive cancers. Additionally, given the relatively small sample sizes of several individualized cancer cohorts included in our pan-cancer analysis and varied methods of measuring outcomes, we next repeated the study, this time restricting our analysis to the cancer genome atlas (TCGA) datasets (N = 12,153). Though the combined TCGA dataset has a smaller combined sample size, these data represent a smaller number of cancer types typically with larger sample sizes in each. Additionally, while outcomes were not available for each for roughly half of patients in the previous pan cancer dataset, in the TCGA dataset survival data was available for nearly all patients.

**Figure 2:**
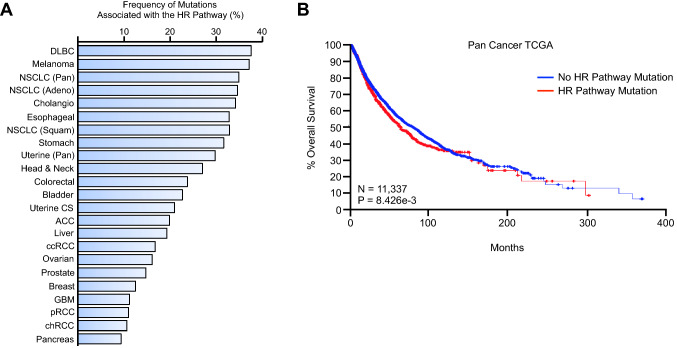
Mutations to genes associated with the homologous recombination pathway are frequent in several cancer histologies for which PARP inhibitors are not currently approved. (**A**) We determined the mutation status of *ATM*, *BARD1*, *BRCA1*, *BRCA2*, *BRIP1*, *CDK12*, *CHEK2*, *DMC1*, *FAAP20*, *FAN1*, *FANCD2*, *FANCE*, *FANCL*, *FANCM*, *PALB2*, *POLQ*, *RAD51*, *RAD51B*, *RAD51C*, *RAD51D*, *RAD54L*, and *XRCC3* in a in The Cancer Genome Atlas (TCGA) pan-cancer cohort of 12,153 patents, and show the combined mutation frequency across all genes by percent for the most represented cancer types. DLBC: diffuse large B cell lymphoma, NSCLC: non-small cell lung cancer, ACC: adenoid cystic carcinoma, ccRCC: clear cell renal cell carcinoma, GBM: glioblastoma multiforme, pRCC: papillary renal cell carcinoma, chRCC: chromophobe renal cell carcinoma. (**B**) Of these 12,153 patients, survival data was available from 11,337, which were used for subsequent analyses. A Kaplan–Meier plot is displayed showing overall survival from patients with or without a mutation to one or more of the genes listed above.

Using this new sample set, we determined the rate of mutations to the HR pathway both overall and by by cancer type (Fig. 2A). HR pathway mutations were particularly common among diffuse large B cell lymphomas and melanoma patients, with combined mutation rates of 37.5 and 37.33%, respectively (Fig. 2A). This was closely followed by lung adenocarcinoma (34.78%), cholangiocarcinoma (34.29%), pan-esophageal cancer (32.97%), squamous cell lung cancer (32.96%), pan-stomach cancer (31.65%), and pan-uterine cancer (30%) (Fig. 2A). Several other cancer types had mutation frequencies between 20 and 30%, including pan-head and neck, colorectal, uterine carcinoma, and adenoid cystic carcinoma (Fig. 2A). Once again, mutations affecting the combined gene set were associated with poor outcomes in the combined cancer cohort (Fig. 2B), with several mutations to select also independently predicting for poor outcomes (Supplementary Table S1).

### Mutations to genes associated with the homologous recombination pathway are heterogeneous and frequently associate with mutations to a variety of unrelated genes

**Figure 3:**
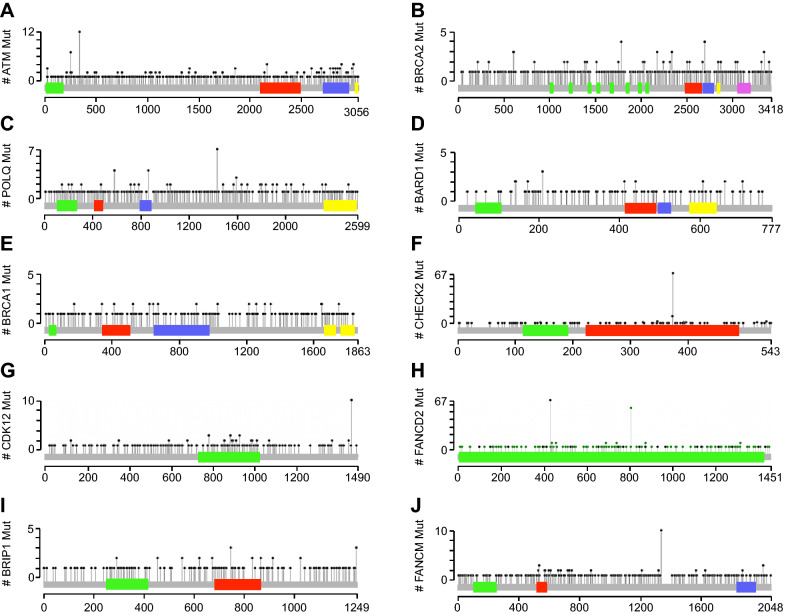
Location of the most frequently represented mutations in genes associated with the homologous recombination pathway in The Cancer Genome Atlas cohort. Lolipop plots displaying the most common mutations to (**A**) *ATM* (**B**) *BRCA2* (**C**) *POLQ* (**D**) *BARD1* (**E**) *BRCA1* (**F**) *CHEK2* (**G**) *CDK12* (**H**) *FANCD2* (**I**) *BRIP1* (**J**) *FANCM*.

We next analyzed the frequency of mutations affecting each individual gene. Once again, *ATM* was the most frequently altered gene, with mutation observed in 4% of all cases (Supplementary Table S2). With respect to *ATM*, we observed a total of 485 mutations, 339 of which were missense, 142 truncating, and 4 in-frame mutations of unknown significance (Fig. 3A). This was followed by *BARD1* (2.51%), *BRCA1* (2.49%), and *BRCA2* (1.98%), each with a similar distribution of mutations. While mutations to *POLQ*, *FANCM*, *CHECK2*, *CDK12*, and *FANCD2* were among the most heterogeneous, these had a relatively low frequency, most effecting only one patient (Fig. 3B–J).

We subsequently analyzed the entirety of the mutations identified in this study using the OncoKB precision oncology knowledge base^39^. This approach predicts for mutations most likely to alter protein function, as well as compares these mutations to those reported in the literature to provide additional insight into which are likely oncogenic, neutral mutations, or of unknown significance. While the majority of mutations identified in this study have yet to be uncharacterized, a sizeable fraction was analogous to those reported previously to have a role in PARP inhibitor sensitivity and/or HRD and likely to have oncogenic function, though this requires further exploration (Supplementary Table S3). Interestingly, several HR-associated mutations often co-occurred in the same patients, suggesting patients with select HR-associated mutations are likely to incur additional HR-associated mutations (Supplementary Table S4). Additionally, patients with HR-associated mutations also harbored mutations to several non-HR genes with higher frequency than those without HR associated mutations (Supplementary Table S5), several of which were also independent predictors of poor clinical outcomes (Supplementary Table S6).

### Mutations to genes associated with the homologous recombination pathway are frequent in several cancer histologies for which PARP inhibitors are not currently approved

In order to identify genes that may be the most useful in determining the status of the HR pathway in select cancer types, we next determined the mutation frequency of these 22 genes in the eight cancers with the highest rate of HR mutation. As mentioned previously, HR mutations were observed most frequently in diffuse large B cell lymphoma, affecting roughly 38% of patients, though this may be inflated given the small sample size of the study (N = 47). In diffuse large B cell lymphoma patients, *ATM* mutations were the most frequently represented, affecting nearly 15% of patients, followed by *POLQ* which was mutated in 10.6% (Supplementary Table S7). Other mutations were less common, but again the relevance of these findings are limited due to the small sample size, and warrant exploration in a larger cohort.

Also as mentioned, HR mutations were also common in cutaneous melanomas (37.5%), though this represented data from 288 patients. In this group, *BRCA* mutations were observed in 11.5% of patients, though mutations to *ATM*, *BRIP1*, *FANCM*, and other genes were also common (Table 3). This was paralleled by both lung adenocarcinoma (N = 660) and squamous lung cancers (N = 484), which had an overall *BRCA* mutation frequency of 7.7% and 10.7%, respectively (Table 4). These cancers also had high rates of *ATM*, *POLQ*, *FANCM* mutations, as well as those to several other genes (Table 5). *ATM*, *BRCA*, *CHECK2*, and *CDK12* mutations were frequent in cholangiocarcinoma (Supplementary Table S8, N = 34) with similar results in uterine carcinoma (Supplementary Table S9, N = 57), though the significance of these results is limited by small sample sizes.

**Table 3:** Mutation frequencies of genes associated with the homologous recombination DNA repair pathway in the TCGA Cutaneous Melanoma cohort (N = 288).

Gene	Observed mutations melanoma (N = 288)	Mutation frequency (%)
ATM	16	5.556
BARD1	1	0.347
BRCA1	15	5.208
BRAC2	20	6.9444
BRCA1/2	33	11.458
BRIP1	15	5.208
CHEK2	7	2.4305
CDK12	4	1.388
DMC1	11	3.819
FAAP20	2	0.694
FAN1	2	0.694
FANCD2	13	4.514
FANCE	2	0.694
FANCL	–	–
FANCM	17	5.903
PALB2	2	0.694
POLQ	7	2.431
RAD51	1	0.347
RAD51B	2	0.694
RAD51C	4	1.389
RAD51D	–	–
RAD54L	–	–
XRCC3	–	–
Any HR mutation	108	37.500

**Table 4:** Mutation frequencies of genes associated with the homologous recombination DNA repair pathway in the TCGA Pan Lung Cancer cohort (N = 1144).

Gene	Observed mutations lung adenocarcinoma (N = 660)	Mutation frequency (%)	Observed mutations lung squamous cell carcinoma (N = 484)	Mutation frequency (%)
ATM	59	8.939	28	5.785
BARD1	15	2.273	7	1.446
BRCA1	24	3.636	24	4.959
BRAC2	30	4.545	30	6.198
BRCA1/2	51	7.727	52	10.744
BRIP1	23	3.485	5	1.033
CHEK2	13	1.967	9	1.860
CDK12	22	3.333	15	3.099
DMC1	1	0.152	0	–
FAAP20	0	–	1	0.207
FAN1	11	1.667	4	0.826
FANCD2	7	1.061	7	1.446
FANCE	5	0.758	1	0.207
FANCL	3	0.455	7	1.446
FANCM	44	6.667	20	4.132
PALB2	13	1.970	13	2.686
POLQ	42	6.364	37	7.645
RAD51	1	0.152	3	0.620
RAD51B	3	0.455	6	1.240
RAD51C	6	0.910	5	1.033
RAD51D	3	0.455	4	0.826
RAD54L	8	1.212	3	0.620
XRCC3	0	–	2	0.413
Any HR mutation	235	35.606	166	34.298

**Table 5:** Mutation frequencies of genes associated with the homologous recombination DNA repair pathway in the TCGA Esophageal cohort (N = 185).

Gene	Observed mutations esophageal adenocarcinoma (N = 89)	Mutation frequency (%)	Observed mutations esophageal squamous cell carcinoma (N = 96)	Mutation frequency (%)
ATM	11	12.360	10	10.417
BARD1	3	3.371	2	2.083
BRCA1	3	3.371	3	3.125
BRAC2	5	5.618	4	4.167
BRCA1/2	7	7.865	7	7.292
BRIP1	3	3.371	2	2.083
CHEK2	3	3.371	2	2.083
CDK12	3	3.371	2	2.083
DMC1	2	2.247	0	–
FAAP20	0	–	0	–
FAN1	1	1.124	0	–
FANCD2	5	5.618	1	1.042
FANCE	1	1.124	0	–
FANCL	0	–	0	–
FANCM	8	8.989	3	3.125
PALB2	1	1.124	0	–
POLQ	4	4.494	4	4.167
RAD51	3	3.371	0	–
RAD51B	1	1.124	0	–
RAD51C	0	–	0	–
RAD51D	0	–	0	–
RAD54L	1	1.124	0	–
XRCC3	0	–	0	–
Any HR mutation	36	40.449	25	26.042

In esophageal cancers, HR mutations were common to both adenocarcinoma (N = 89) and squamous (N = 96) cancers, though they were more frequent to the former (Table 5). While *ATM*, *BRCA*, and *POLQ* mutations were similarly prevalent in both cancer types, adenocarcinoma patients had a high frequency to mutations effecting *FANCM* (8.9%) and *FANCD2* (5.6%), comprising a majority of the difference between the two cancers in overall HR mutation rate (Table 5).

In stomach cancer, mutation rates also varied extensively depending on cancer subtype. For instance, in the four subtypes represented in the TCGA stomach adenocarcinoma cohort, HR mutations were most common in mucinous adenocarcinoma by percent at 41%, though this represents a very small sample size of only 21 patients (Table 6). In tubular (N = 61), diffuse adenocarcinomas (N = 70), and non-specified carcinomas (N = 228), rates were 37.7%, 21.4%, and 32.5% respectively (Table 6). However, the relative distribution of HR mutation among subtypes were varied, though all subtypes had relatively high rates of *ATM*, *BRCA*, and *POLQ* mutations, with *FANCM* mutations common to mucinous and non-specified carcinomas (Table 6). Finally, we evaluated squamous cancers of the head and neck (N = 512), which had an overall HR mutation frequency of 27.1%. This group had little in the way of *ATM* mutations (2.9%), though we observed comparatively high rates of *BRCA*, *CHECK* and *POLQ* mutations (Table 7).

**Table 6:** Mutation frequencies of genes associated with the homologous recombination DNA repair pathway in the TCGA Stomach adenocarcinoma cohort (N = 395).

Gene	Observed mutations mucinous adenocarcinoma (N = 21)	Observed mutations tubular adenocarcinoma (N = 61)	Observed mutations diffuse adenocarcinoma (N = 70)	Observed mutations non-specified adenocarcinoma (N = 288)
ATM	4 (19.05%)	5 (8.20%)	3 (4.29%)	26 (11.40%)
BARD1	0 (0%)	2 (3.28%)	1 (1.43%)	10 (4.39%)
BRCA1	1 (4.76%)	3 (4.92%)	2 (2.86%)	9 (3.95%)
BRAC2	3 (4.29%)	5 (8.20%)	5 (7.14%)	24 (10.53%)
BRCA1/2	4 (19.05%)	6 (9.84%)	6 (8.57%)	30 (13.16%)
BRIP1	2 (9.52%)	2 (3.28%)	0 (0%)	2 (0.88%)
CHEK2	1 (4.76%)	2 (3.28%)	0 (0%)	6 (2.63%)
CDK12	0 (0%)	5 (8.20%)	2 (2.86%)	12 (5.26%)
DMC1	1 (4.76%)	0 (0%)	0 (0%)	7 (3.07%)
FAAP20	1 (4.76%)	0 (0%)	1 (1.43%)	1 (0.44%)
FAN1	0 (0%)	3 (4.92%)	0 (0%)	8 (3.51%)
FANCD2	2 (9.52%)	2 (3.28%)	2 (2.86%)	9 (3.95%)
FANCE	0 (0%)	0 (0%)	1 (1.43%)	6 (2.63%)
FANCL	1 (4.76%)	0 (0%)	1 (1.43%)	3 (1.32%)
FANCM	2 (9.52%)	2 (3.28%)	5 (7.14%)	20 (8.77%)
PALB2	1 (4.76%)	1 (1.64%)	0 (0%)	9 (3.95%)
POLQ	4 (19.05%)	2 (3.28%)	1 (1.43%)	24 (10.53%)
RAD51	0 (0%)	0 (0%)	0 (0%)	1 (0.44%)
RAD51B	0 (0%)	0 (0%)	0 (0%)	2 (0.88%)
RAD51C	0 (0%)	0 (0%)	0 (0%)	2 (0.88%)
RAD51D	1 (4.76%)	2 (3.28%)	0 (0%)	1 (0.44%)
RAD54L	0 (0%)	0 (0%)	0 (0%)	4 (1.75%)
XRCC3	0 (0%)	1 (1.64%)	0 (0%)	3 (1.32%)
Any HR mutation	9 (42.68%)	23 (37.70%)	15 (21.43%)	74 (32.46%)

**Table 7:** Mutation frequencies of genes associated with the homologous recombination DNA repair pathway in the TCGA Head & Neck squamous cell carcinoma cohort (N = 512).

Gene	Observed mutations head and neck squamous cell carcinoma (N = 512)	Mutation frequency (%)
ATM	15	2.930
BARD1	9	1.758
BRCA1	11	2.148
BRAC2	23	4.492
BRCA1/2	34	6.641
BRIP1	13	2.539
CHEK2	21	4.102
CDK12	7	1.367
DMC1	1	0.195
FAAP20	–	–
FAN1	5	0.977
FANCD2	10	1.953
FANCE	–	–
FANCL	4	0.781
FANCM	10	1.953
PALB2	7	1.367
POLQ	22	4.2975
RAD51	1	0.195
RAD51B	1	0.195
RAD51C	3	0.586
RAD51D	2	0.391
RAD54L	5	0.977
XRCC3	–	–
Any HR mutation	139	27.148

## Discussion

The efficacy of PARP inhibitors in *BRCA*-mutated tumors stems largely from the known roles for PARP in mediating single stranded break repair^40,41^. Thus, initial trials were based on the hypothesis that inhibiting the repair of single stranded breaks will cause synthetic lethality in tumors with loss of high-fidelity double-strand break homologous recombination^40^. As discussed, this approach has shown tremendous efficacy in several *BRCA*-mutant cancers, including those of the breast, ovary, prostate, colon, thymus, and pancreas^10,13,42^. Olaparib became the first FDA-approved PARP inhibitor based on results from Study 19, a randomized, placebo-controlled trial in ovarian cancer showing an improvement in both progression-free and overall survival^43^.

Additionally, olaparib was soon approved for *BRCA*-mutated breast cancer following the phase III OlympiAD trial, which showed improvements in both response rate and progression-free survival when compared to standard therapy^44^. Subsequently, PARP inhibitors have shown efficacy in the second line, and olaparib, rucaparib and niraparib have now been approved as maintenance therapy for HR deficient ovarian cancer patients following platinum-based chemotherapy^45–47^. However, while PARP inhibitors have no doubt improved clinical outcomes in *BRCA*-mutated tumors, there is mounting biologic evidence that other molecular subsets may also derive clinical benefit from PARP inhibitors^30^. These include patients with genomic alterations in *ATM*, *BARD1*, *BRIP1*, *CHEK2*, *FAAP20*, *FAN1*, *FANCE*, *FANCM*, *PALB2*, *POLQ*, *RAD51B*, *RAD51C*, and *RAD51D*^31–33^. While mutations of these and other HR genes are certainly less established indicators of HRD, those affecting *ATM* and *PALB2* have already been shown to associate with responsiveness to PARP inhibition^14^.

Thus, when evaluating a pan-cancer cohort, we found that by expanding our search to include several HR genes beyond those most frequently associated with PARP inhibitors, there may be several additional patient groups who also have genetic loss of HRD and may therefore also respond to PARP inhibition. This is consistent to results observed in a similar study, which also found that expanding criteria identifies a larger group of patients who potentially harbor defects to the HR pathway^48^. In our study, when restricting our analysis to *BRCA*-mutated tumors, we found that only ~ 4% of patients are represented. When including *ATM*-mutated tumors, this number more than doubles to 8.36%. However, when including the other genes in our panel, as many as 13.36% of patients are now represented. While we cannot conclusively state that the entirety of these patients are in fact HR deficient and would derive clinical benefit from PARP inhibition, as mutations to *BARD1*, *CDK12*, *DMC1*, *PALB2*, and *POLQ* seem to predict for poor outcomes in this cohort, their predictive value for PARP inhibition is not established and warrants continued exploration.

This is particularly true for the many cancer histologies identified in this study for which PARP inhibitors are not widely used or FDA approved. For instance, though limited by a small sample size, we found that nearly 40% of diffuse large B cell lymphoma patients harbor mutations to genes associated with the HR pathway, though *BRCA* mutations were only observed in 6.38%. Though early evidence supports the addition of the PARP inhibitor veliparib to bendamustine and rituximab in B-cell lymphomas^49^, the role for PARP inhibitors in diffuse large B-cell lymphoma is still under investigation. Still, recent evidence points to additional predictive criteria expanding beyond *BRCA* mutations, with less-studied HR-associated genes such as *LMO2* appearing to predict for sensitivity to PARP inhibition^50^.

As discussed, we also identified a high frequency in HR mutations in cutaneous melanoma patients. Murine models have supported a pro-metastatic role for PARP-1, and PARP inhibition is showing early promise in combination with radiotherapy in murine models of uveal melanoma ^51,52^. However, like with diffuse large B cell lymphoma, clinical data is rather limited. A 2013 phase II study suggests that the PARP inhibitor rucaparib cooperates with temozolomide in metastatic melanoma^53^, though there are a relatively small number of subsequent clinical studies, likely as *BRCA1/2* mutations are not typically considered a cause of malignant melanoma^54^. However, in the TCGA cohort examined in our study, we found that *BRCA* mutations are represented in as many as 11.5% of cutaneous melanoma patients, with many patients also harboring mutations to *ATM*, *BRIP1*, *CHECK2*, *DMC1*, *FANCD2*, *FANCM*, and *POLQ*. As 37.5% of this patient cohort had at least one mutation affecting the HR pathway, the use of these and other mutations warrant consideration when exploring PARP inhibitors in subsequent clinical trials.

Using this expanded gene panel, we found that HRD in lung, bile duct, esophageal, stomach, uterine, and head and neck cancers may also be underreported. This may be of clinical significance, as PARP inhibitors are showing early promise in several of these cancer histologies, particularly when combined with chemotherapy or radiation^55–64^. However, we must note that an inherent limitation of our study is though we identified several mutations in HR associated genes, relatively few have been characterized, particularly with respect to either HRD or PARP inhibition. Additionally, as our data is largely dependent on sequencing from formalin fixed paraffin embedded tissues, these rates of mutation may be inflated due to technical artifacts. Hence, it is not clear how many patients identified in this study will in fact have HRD or would benefit from PARP inhibition. Additionally, clinical response to PARP inhibition is not solely driven by HRD, involving several other factors including replication, oxidative, and ER stress^65–69^.

Therefore, specific alterations to these and other genes warrant further investigation prior to any being proposed as a reliable surrogate for HRD, particularly in the setting of other cellular processes. Further, should PARP inhibitors be combined with other DNA-damaging agents such as chemo or radiotherapy, a patient’s HRD status may become less relevant, as early evidence suggests that such approaches may have efficacy in multiple *TP53* mutated but HR-intact tumor types^70^. However, improving the selection criteria for PARP inhibition in monotherapy or without additional DNA-damaging agents will require careful evaluation of these and potentially other HR associated genes in hopes of identifying the patients who will most benefit from this approach.

## Supplementary information

Supplementary Information.

## Abbreviations

PARP: Poly ADP ribose polymerase
HR: Homologous recombination
NGS: Next generation sequencing
NSCLC: Non-small cell lung cancer
TCGA: The Cancer Genome Atlas
