# Supplementary material for: Frequency and prognostic value of mutations associated with the homologous recombination DNA repair pathway in a large pan cancer cohort
Source: Sci Rep. 2020 Nov 19;10:20223. doi: 10.1038/s41598-020-76975-6 (PMC7677533; doi:10.1038/s41598-020-76975-6)
Supplement: Supplementary file 1 — Supplementary Information. [file 41598_2020_76975_MOESM1_ESM.docx]

**Supplement: Frequency and Prognostic Value of Mutations Associated with the Homologous Recombination DNA Repair Pathway in a Large Pan Cancer Cohort**

Daniel R. Principe^1,2*^, Matthew Narbutis^1*^, Regina Koch^3^, and Ajay Rana^1,4#^

^1^Department of Surgery, University of Illinois at Chicago, Chicago, IL, ^2^Medical Scientist Training Program, University of Illinois College of Medicine, Chicago, IL, ^3^University of Illinois College of Medicine, Chicago, IL, ^4^Jesse Brown VA Medical Center, Chicago, IL.

*Joint first authors.

Short Title: Homologous Recombination Mutation Frequency in Cancer

Pages 78

Tables 9

Words 26,360

^#^Correspondence to:

Ajay Rana, Ph.D.

Endowed Professor

Department of Surgery, Division of Surgical Oncology

College of Medicine, The University of Illinois at Chicago

840 S. Wood Street

Suite 601 Clinical Sciences Building

Chicago, IL 60612

Tel: (312) 413-7271

Email: [arana@uic.edu](mailto:arana@uic.edu)

**Conflict of Interest Disclosure:** The authors have no conflicts to disclose.

***List of studies included in Pan-Cancer genome analysis***

Data from each of the following studies was compiled and visualized as described above: Acinar Cell Carcinoma of the Pancreas (JHU, J Pathol 2014); Acral Melanoma (TGEN, Genome Res 2017); Acute Lymphoblastic Leukemia (St Jude, Nat Genet 2016); Adenoid Cystic Carcinoma (FMI, Am J Surg Pathl. 2014); Adenoid Cystic Carcinoma (JHU, Cancer Prev Res 2016); Adenoid Cystic Carcinoma (MDA, Clin Cancer Res 2015); Adenoid Cystic Carcinoma (MGH, Nat Gen 2016); Adenoid Cystic Carcinoma (MSKCC, Nat Genet 2013); Adenoid Cystic Carcinoma (Sanger/MDA, JCI 2013); Adenoid Cystic Carcinoma of the Breast (MSKCC, J Pathol. 2015); Ampullary Carcinoma (Baylor College of Medicine, Cell Reports 2016); Bladder Cancer (MSKCC, Eur Urol 2014); Bladder Cancer (MSKCC, J Clin Onco 2013); Bladder Urothelial Carcinoma (BGI, Nat Genet 2013); Bladder Urothelial Carcinoma (DFCI/MSKCC, Cancer Discov 2014); Breast Cancer (METABRIC, Nature 2012 & Nat Commun 2016); Breast Fibroepithelial Tumors (Duke-NUS, Nat Genet 2015); Breast Invasive Carcinoma (British Columbia, Nature 2012); Breast Invasive Carcinoma (Broad, Nature 2012); Breast Invasive Carcinoma (Sanger, Nature 2012); Cholangiocarcinoma (National Cancer Centre of Singapore, Nat Genet 2013); Cholangiocarcinoma (National University of Singapore, Nat Genet 2012); Chronic Lymphocytic Leukemia (Broad, Cell 2013); Chronic Lymphocytic Leukemia (IUOPA, Nature 2015); Chronic lymphocytic leukemia (ICGC, Nature Genetics 2011); Colorectal Adenocarcinoma (DFCI, Cell Reports 2016); Colorectal Adenocarcinoma (Genentech, Nature 2012); Colorectal Adenocarcinoma Triplets (MSKCC, Genome Biol 2014); Cutaneous Squamous Cell Carcinoma (DFCI, Clin Cancer Res 2015); Cutaneous Squamous Cell Carcinoma (MD Anderson, Clin Cancer Res 2014); Cutaneous T Cell Lymphoma (Columbia U, Nat Genet 2015); Cystic Tumor of the Pancreas (Johns Hopkins, PNAS 2011); Desmoplastic Melanoma (Broad Institute, Nat Genet 2015); Diffuse Large B cell Lymphoma (DFCI, Nat Med 2018); Diffuse Large B-Cell Lymphoma (Duke, Cell 2017); Diffuse Large B-cell Lymphoma (BCGSC, Blood 2013); Esophageal Adenocarcinoma (DFCI, Nat Genet 2013); Esophageal Squamous Cell Carcinoma (ICGC, Nature 2014); Esophageal Squamous Cell Carcinoma (UCLA, Nat Genet 2014); Ewing Sarcoma (Institut Curie, Cancer Discov 2014); Gallbladder Carcinoma (Shanghai, Nat Genet 2014); Gastric Adenocarcinoma (TMUCIH, PNAS 2015); Glioma (MSK, 2018); Head and Neck Squamous Cell Carcinoma (Broad, Science 2011); Head and Neck Squamous Cell Carcinoma (Johns Hopkins, Science 2011); Hepatocellular Adenoma (INSERM, Cancer Cell 2014); Hepatocellular Carcinomas (INSERM, Nat Genet 2015); Histiocytosis Cobimetinib (MSK, Nature 2019); Insulinoma (Shanghai, Nat Commun 2013); Intrahepatic Cholangiocarcinoma (JHU, Nat Genet 2013); Kidney Renal Clear Cell Carcinoma (BGI, Nat Genet 2012); Kidney Renal Clear Cell Carcinoma (IRC, Nat Genet 2014); Liver Hepatocellular Adenoma and Carcinomas (MSK, PLOS One 2018); Liver Hepatocellular Carcinoma (AMC, Hepatology 2014); Liver Hepatocellular Carcinoma (RIKEN, Nat Genet 2012); Low-Grade Gliomas (UCSF, Science 2014); Lung Adenocarcinoma (Broad, Cell 2012); Lung Adenocarcinoma (TSP, Nature 2008); MSK-IMPACT Clinical Sequencing Cohort (MSKCC, Nat Med 2017); MSS Mixed Solid Tumors (Broad/Dana-Farber, Nat Genet 2018); Malignant Peripheral Nerve Sheath Tumor (MSKCC, Nat Genet 2014); Mantle Cell Lymphoma (IDIBIPS, PNAS 2013); Medulloblastoma (Broad, Nature 2012); Medulloblastoma (PCGP, Nature 2012); Medulloblastoma (Sickkids, Nature 2016); Metastatic Breast Cancer (INSERM, PLoS Med 2016); Metastatic Melanoma (MSKCC, JCO Precis Oncol 2017); Metastatic Melanoma (UCLA, Cell 2016); Metastatic Prostate Adenocarcinoma (MCTP, Nature 2012); Metastatic Prostate Adenocarcinoma (SU2C/PCF Dream Team, PNAS 2019); Metastatic Prostate Cancer (SU2C/PCF Dream Team, Cell 2015); Multiple Myeloma (Broad, Cancer Cell 2014); Myelodysplasia (UTokyo, Nature 2011); Nasopharyngeal Carcinoma (Singapore, Nat Genet 2014); Neuroblastoma (AMC Amsterdam, Nature 2012); Neuroblastoma (Broad, Nature 2015); Neuroendocrine Prostate Cancer (Multi-Institute, Nat Med 2016); Non-Hodgkin Lymphoma (BCGSC, Nature 2011); Non-Small Cell Lung Cancer (University of Turin, Lung Cancer 2017); Oral Squamous Cell Carcinoma (MD Anderson, Cancer Discov 2013); Pancreatic Adenocarcinoma (QCMG, Nature 2016); Pancreatic Cancer (UTSW, Nat Commun 2015); Pancreatic Neuroendocrine Tumors (Johns Hopkins University, Science 2011); Pancreatic Neuroendocrine Tumors (Multi-Institute, Nature 2017); Pediatric Acute Lymphoid Leukemia - Phase II (TARGET, 2018); Pediatric Acute Myeloid Leukemia (TARGET, 2018); Pediatric Ewing Sarcoma (DFCI, Cancer Discov 2014); Pediatric Neuroblastoma (TARGET, 2018); Pediatric Pan-Cancer (DKFZ, Nature 2017); Pediatric Pan-cancer (Columbia U, Genome Med 2016); Pediatric Rhabdoid Tumor (TARGET, 2018); Pediatric Wilms' Tumor (TARGET, 2018); Pleural Mesothelioma (NYU, Cancer Res 2015); Primary Central Nervous System Lymphoma (Mayo Clinic, Clin Cancer Res 2015); Prostate Adenocarcinoma (Broad/Cornell, Nat Genet 2012); Prostate Adenocarcinoma (Fred Hutchinson CRC, Nat Med 2016); Prostate Adenocarcinoma (MSKCC, Cancer Cell 2010); Prostate Adenocarcinoma (SMMU, Eur Urol 2017); Prostate Adenocarcinoma Organoids (MSKCC, Cell 2014); Renal Clear Cell Carcinoma (UTokyo, Nat Genet 2013); Renal Non-Clear Cell Carcinoma (Genentech, Nat Genet 2014); Rhabdoid Cancer (BCGSC, Cancer Cell 2016); SUMMIT - Neratinib Basket Study (Multi-Institute, Nature 2018); Skin Cutaneous Melanoma (Broad, Cell 2012); Skin Cutaneous Melanoma (Yale, Nat Genet 2012); Skin Cutaneous Melanoma(Broad, Cancer Discov 2014); Small Cell Carcinoma of the Ovary (MSKCC, Nat Genet 2014); Small Cell Lung Cancer (Johns Hopkins, Nat Genet 2012); Small Cell Lung Cancer (U Cologne, Nature 2015); Small-Cell Lung Cancer (Multi-Institute, Cancer Cell 2017); Squamous Cell Carcinoma of the Vulva (CUK, Exp Mol Med 2018); Stomach Adenocarcinoma (Pfizer and UHK, Nat Genet 2014); Stomach Adenocarcinoma (U Tokyo, Nat Genet 2014); The Metastatic Breast Cancer Project (Provisional, October 2018); The Metastatic Prostate Cancer Project (Provisional, December 2018); Thoracic PDX (MSK, Provisional); Thymic Epithelial Tumors (NCI, Nat Genet 2014); Unclassified Renal Cell Carcinoma (MSK, Nature 2016); Upper Tract Urothelial Cancer (MSK, Eur Urol 2015); Upper Tract Urothelial Carcinoma (Cornell/Baylor/MDACC, Nat Comm 2019); Urothelial Carcinoma (Cornell/Trento, Nat Gen 2016); Uveal Melanoma (QIMR, Oncotarget 2016); Pan-Lung Cancer (TCGA, Nat Genet 2016); Adrenocortical Carcinoma (TCGA, Provisional); Cholangiocarcinoma (TCGA, Provisional); Cholangiocarcinoma (MSK, Clin Cancer Res 2018); Gallbladder Cancer (MSK, Cancer 2018); Bladder Cancer (TCGA, Cell 2017); Bladder Cancer (MSKCC, Nat Genet 2016); Nonmuscle Invasive Bladder Cancer (MSK Eur Urol 2017); Bladder Urothelial Carcinoma (TCGA, Provisional); Metastatic Colorectal Cancer (MSKCC, Cancer Cell 2018); Colorectal Adenocarcinoma (TCGA, Provisional); Breast Cancer (MSK, Cancer Cell 2018); Breast Cancer Xenografts (British Columbia, Nature 2015); Breast Invasive Carcinoma (TCGA, Provisional); Brain Lower Grade Glioma (TCGA, Provisional); Merged Cohort of LGG and GBM (TCGA, Cell 2016); Glioblastoma Multiforme (TCGA, Provisional); Anaplastic Oligodendroglioma and Anaplastic Oligoastrocytoma (MSKCC, Neuro Oncol 2017); Medulloblastoma (ICGC, Nature 2012); Pilocytic Astrocytoma (ICGC, Nature Genetics 2013); Cervical Squamous Cell Carcinoma and Endocervical Adenocarcinoma (TCGA, Provisional); TCGA data for Esophagus-Stomach Cancers (TCGA, Nature 2017); Metastatic Esophagogastric Cancer (MSKCC, Cancer Discovery 2017); Esophageal Carcinoma (TCGA, Provisional); Stomach Adenocarcinoma (TCGA, Provisional); Stomach Adenocarcinoma (UHK, Nat Genet 2011); Uveal Melanoma (TCGA, Provisional); Head and Neck Squamous Cell Carcinoma (TCGA, Provisional); Kidney Renal Clear Cell Carcinoma (TCGA, Provisional); Kidney Chromophobe (TCGA, Provisional); Kidney Renal Papillary Cell Carcinoma (TCGA, Provisional); Hepatocellular Carcinoma (MSK, Clin Cancer Res 2018); Liver Hepatocellular Carcinoma (TCGA, Provisional); Small Cell Lung Cancer (CLCGP, Nat Genet 2012); Non-Small Cell Lung Cancer (MSKCC, J Clin Oncol 2018); Lung Adenocarcinoma (TCGA, Provisional); Lung Adenocarcinoma (MSKCC, Science 2015); Non-Small Cell Cancer (MSKCC, Cancer Discov 2017); Lung Squamous Cell Carcinoma (TCGA, Provisional); Acute Lymphoblastic Leukemia (St Jude, Nat Genet 2015); Diffuse Large B-Cell Lymphoma (Broad, PNAS 2012); Lymphoid Neoplasm Diffuse Large B-cell Lymphoma (TCGA, Provisional); Mature B-cell malignancies (MD Anderson Cancer Center); Acute Myeloid Leukemia (TCGA, Provisional); Hypodiploid Acute Lymphoid Leukemia (St Jude, Nat Genet 2013); Recurrent and Metastatic Head & Neck Cancer (MSKCC, JAMA Oncol 2016); TMB and Immunotherapy (MSKCC, Nat Genet 2019); Ovarian Serous Cystadenocarcinoma (TCGA, Provisional); Pancreatic Adenocarcinoma (TCGA, Provisional); Pancreatic Adenocarcinoma (ICGC, Nature 2012); Neuroblastoma (Broad, Nat Genet 2013); Mesothelioma (TCGA, Provisional); Prostate Adenocarcinoma (Broad/Cornell, Cell 2013); Prostate Adenocarcinoma (CPC-GENE, Nature 2017); Prostate Adenocarcinoma (MSKCC, PNAS 2014); Prostate Adenocarcinoma (MSKCC/DFCI, Nature Genetics 2018); Prostate Adenocarcinoma (TCGA, Provisional); Prostate Cancer (MSKCC, JCO Precis Oncol 2017); Melanoma (Broad/Dana Farber, Nature 2012); Skin Cutaneous Melanoma (TCGA, Provisional); Pheochromocytoma and Paraganglioma (TCGA, PanCancer Atlas); Rhabdomyosarcoma (NIH, Cancer Discov 2014); Sarcoma (MSKCC/Broad, Nat Genet 2010); Sarcoma (TCGA, Provisional); The Angiosarcoma Project - Count Me In (Provisional, September 2018); Germ Cell Tumors (MSKCC, J Clin Oncol 2016); Testicular Germ Cell Cancer (TCGA, Provisional); Thymoma (TCGA, Provisional); Poorly-Differentiated and Anaplastic Thyroid Cancers (MSKCC, JCI 2016); Thyroid Carcinoma (TCGA, Provisional); Endometrial Cancer (MSK, 2018); Uterine Carcinosarcoma (Johns Hopkins, Nat Commun 2014); Uterine Carcinosarcoma (TCGA, Provisional); Uterine Clear Cell Carcinoma (NIH, Cancer 2017); Uterine Corpus Endometrial Carcinoma (TCGA, Provisional).

| Gene | Median Months Survival without Mutation | Median Months Survival with Mutation | P Value |
| --- | --- | --- | --- |
| All Genes | 78.15 | 62.89 | P = 8.43x10^-3^ |
| ATM | 73.62 | 102.04 | P = 0.148 |
| BARD1 | 74.87 | 75.10 | P = 0.174 |
| BRCA1 | 74.87 | 95.27 | P = 0.380 |
| BRAC2 | 74.67 | 76.30 | P = 0.713 |
| BRCA1/2 | 74.87 | 76.15 | P = 0.663 |
| BRIP1 | 74.67 | 96.16 | P = 0.895 |
| CHEK2 | 74.87 | N/A | P = 0.289 |
| CDK12 | 75.10 | 53.15 | P = 0.264 |
| DMC1 | 75.10 | 61.24 | P = 0.849 |
| FAAP20 | 75.03 | 37.71 | P = 0.0977 |
| FAN1 | 74.87 | 76.97 | P = 0.987 |
| FANCD2 | 75.03 | 51.12 | P = 0.226 |
| FANCE | 74.67 | N/A | P = 0.0567 |
| FANCL | 75.03 | 62.89 | P = 0.926 |
| FANCM | 75.10 | 48.82 | P = 0.289 |
| PALB2 | 75.10 | 54.47 | P = 0.640 |
| POLQ | 75.13 | 61.24 | P = 0.181 |
| RAD51 | 74.97 | 72.01 | P = 0.711 |
| RAD51B | 74.67 | 173.91 | P = 0.0190 |
| RAD51C | 75.03 | 32.5 | P = 0.315 |
| RAD51D | 74.97 | 60.84 | P = 0.334 |
| RAD54L | 75.03 | 52.00 | P = 0.411 |
| XRCC3 | 74.97 | N/A | P = 0.594 |

**Table S1. Select mutations to genes associated with the homologous recombination DNA repair pathway are associated with poor survival in the TCGA pan cancer cohort (N = 12,153)**

| Gene | Observed Mutations Pan Cancer (N=12,153) | Mutation Frequency (%) |
| --- | --- | --- |
| ATM | 394 | 3.241997861 |
| BARD1 | 305 | 2.509668395 |
| BRCA1 | 303 | 2.493211553 |
| BRAC2 | 240 | 1.974821032 |
| BRIP1 | 176 | 1.44820209 |
| CHEK2 | 174 | 1.431745248 |
| CDK12 | 138 | 1.135522093 |
| DMC1 | 137 | 1.127293672 |
| FAAP20 | 106 | 0.872212622 |
| FAN1 | 93 | 0.76524315 |
| FANCD2 | 79 | 0.650045256 |
| FANCE | 61 | 0.501933679 |
| FANCL | 55 | 0.452563153 |
| FANCM | 41 | 0.33736526 |
| PALB2 | 38 | 0.312679997 |
| POLQ | 32 | 0.263309471 |
| RAD51 | 29 | 0.238624208 |
| RAD51B | 28 | 0.230395787 |
| RAD51C | 28 | 0.230395787 |
| RAD51D | 15 | 0.123426314 |
| RAD54L | 12 | 0.098741052 |
| XRCC3 | 12 | 0.098741052 |
| Any HR Mutation | 1814 | 14.926355632 |

**Table S2. Mutation frequencies of genes associated with the homologous recombination DNA repair pathway in the TCGA pan cancer cohort (N = 12,153)**

| **ATM Mutation** | **Mutation Type** | **Cancer Type** | **OncoKB Analysis** |
| --- | --- | --- | --- |
| R337H | Missense Mutation | Bladder Urothelial Carcinoma | Likely Oncogenic |
| R337H | Missense Mutation | Breast Invasive Ductal Carcinoma | Likely Oncogenic |
| R337C | Missense Mutation | Hepatocellular Carcinoma | Likely Oncogenic |
| R337C | Missense Mutation | Pancreatic Adenocarcinoma | Likely Oncogenic |
| R337C | Missense Mutation | Stomach Adenocarcinoma | Likely Oncogenic |
| R337C | Missense Mutation | Stomach Adenocarcinoma | Likely Oncogenic |
| R337C | Missense Mutation | Prostate Adenocarcinoma | Likely Oncogenic |
| R337C | Missense Mutation | Cutaneous Melanoma | Likely Oncogenic |
| R337C | Missense Mutation | Cutaneous Melanoma | Likely Oncogenic |
| R337C | Missense Mutation | Head and Neck Squamous Cell Carcinoma | Likely Oncogenic |
| R337C | Missense Mutation | Lung Adenocarcinoma | Likely Oncogenic |
| R337C | Missense Mutation | Lung Adenocarcinoma | Likely Oncogenic |
| R3008H | Missense Mutation | Lung Adenocarcinoma | Likely Oncogenic |
| R3008H | Missense Mutation | Lung Adenocarcinoma | Likely Oncogenic |
| R3008C | Missense Mutation | Stomach Adenocarcinoma | Oncogenic |
| R3008C | Missense Mutation | Lung Adenocarcinoma | Oncogenic |
| R3008L | Missense Mutation | Lung Adenocarcinoma | Likely Oncogenic |
| D1853N | Missense Mutation | Diffuse Large B-Cell Lymphoma | Likely Neutral |
| D1853N | Missense Mutation | Diffuse Large B-Cell Lymphoma | Likely Neutral |
| D1853N | Missense Mutation | Head and Neck Squamous Cell Carcinoma | Likely Neutral |
| R2832C | Missense Mutation | Stomach Adenocarcinoma | Likely Oncogenic |
| R2832C | Missense Mutation | Lung Adenocarcinoma | Likely Oncogenic |
| R2832S | Missense Mutation | Lung Adenocarcinoma | Likely Oncogenic |
| R250* | Nonsense Mutation | Uterine Endometrioid Carcinoma | Likely Oncogenic |
| R250* | Nonsense Mutation | Uterine Endometrioid Carcinoma | Likely Oncogenic |
| R250* | Nonsense Mutation | Renal Clear Cell Carcinoma | Likely Oncogenic |
| R250* | Nonsense Mutation | Colon Adenocarcinoma | Likely Oncogenic |
| R250* | Nonsense Mutation | Rectal Adenocarcinoma | Likely Oncogenic |
| L2890V | Missense Mutation | Stomach Adenocarcinoma | Likely Oncogenic |
| L2890V | Missense Mutation | Esophageal Squamous Cell Carcinoma | Likely Oncogenic |
| L2890R | Missense Mutation | Lung Adenocarcinoma | Likely Oncogenic |
| L2890R | Missense Mutation | Lung Adenocarcinoma | Likely Oncogenic |
| R250Sfs*3 | Frame Shift Deletion | Lung Adenocarcinoma | Likely Oncogenic |
| R250Sfs*3 | Frame Shift Deletion | Lung Adenocarcinoma | Likely Oncogenic |
| G2695S | Missense Mutation | Prostate Adenocarcinoma | Unknown |
| G2695S | Missense Mutation | Lung Adenocarcinoma | Unknown |
| G2695S | Missense Mutation | Lung Adenocarcinoma | Unknown |
| G2695C | Missense Mutation | Lung Adenocarcinoma | Unknown |
| G2695C | Missense Mutation | Lung Adenocarcinoma | Unknown |
| F570L | Missense Mutation | Uterine Endometrioid Carcinoma | Unknown |
| R3047* | Nonsense Mutation | Uterine Endometrioid Carcinoma | Likely Oncogenic |
| N2875S | Missense Mutation | Prostate Adenocarcinoma | Likely Oncogenic |
| N2875S | Missense Mutation | Breast Invasive Ductal Carcinoma | Likely Oncogenic |
| N2875K | Missense Mutation | Prostate Adenocarcinoma | Likely Oncogenic |
| L2427R | Missense Mutation | Lung Adenocarcinoma | Likely Oncogenic |
| Y2954C | Missense Mutation | Serous Ovarian Cancer | Unknown |
| E2164K | Missense Mutation | Prostate Adenocarcinoma | Unknown |
| E2164K | Missense Mutation | Breast Invasive Ductal Carcinoma | Unknown |
| E2164G | Missense Mutation | Bladder Urothelial Carcinoma | Unknown |
| E2164Q | Missense Mutation | Bladder Urothelial Carcinoma | Unknown |
| I2888L | Missense Mutation | Bladder Urothelial Carcinoma | Predicted Oncogenic |
| D2725G | Missense Mutation | Renal Clear Cell Carcinoma | Unknown |
| S2394L | Missense Mutation | Lung Squamous Cell Carcinoma | Likely Oncogenic |
| R2993* | Nonsense Mutation | Uterine Endometrioid Carcinoma | Likely Oncogenic |
| R2993* | Nonsense Mutation | Breast Invasive Ductal Carcinoma | Likely Oncogenic |
| R2993* | Nonsense Mutation | Breast Invasive Ductal Carcinoma | Likely Oncogenic |
| R1730* | Nonsense Mutation | Mucinous Adenocarcinoma of the Colon and Rectum | Likely Oncogenic |
| R1730* | Nonsense Mutation | Rectal Adenocarcinoma | Likely Oncogenic |
| R2691C | Missense Mutation | Uterine Endometrioid Carcinoma | Likely Oncogenic |
| R2691C | Missense Mutation | Uterine Endometrioid Carcinoma | Likely Oncogenic |
| R2691C | Missense Mutation | Cutaneous Melanoma | Likely Oncogenic |
| R248Q | Missense Mutation | Uterine Endometrioid Carcinoma | Unknown |
| L1408I | Missense Mutation | Uterine Endometrioid Carcinoma | Unknown |
| L1408I | Missense Mutation | Rectal Adenocarcinoma | Unknown |
| D2721N | Missense Mutation | Colon Adenocarcinoma | Unknown |
| L1408F | Missense Mutation | Stomach Adenocarcinoma | Unknown |
| D1208H | Missense Mutation | Serous Ovarian Cancer | Unknown |
| E2423G | Missense Mutation | Pancreatic Adenocarcinoma | Unknown |
| M1040V | Missense Mutation | Diffuse Large B-Cell Lymphoma | Unknown |
| R1618* | Nonsense Mutation | Uterine Carcinosarcoma/Uterine Malignant Mixed Mullerian Tumor | Likely Oncogenic |
| E522* | Nonsense Mutation | Uterine Endometrioid Carcinoma | Likely Oncogenic |
| D2720N | Missense Mutation | Breast Invasive Lobular Carcinoma | Unknown |
| L1439I | Missense Mutation | Uterine Endometrioid Carcinoma | Unknown |
| R23Q | Missense Mutation | Uterine Endometrioid Carcinoma | Unknown |
| R23Q | Missense Mutation | Stomach Adenocarcinoma | Unknown |
| S2812Y | Missense Mutation | Uterine Endometrioid Carcinoma | Unknown |
| S2812Y | Missense Mutation | Tubular Stomach Adenocarcinoma | Unknown |
| R2526G | Missense Mutation | Lung Adenocarcinoma | Unknown |
| D2720H | Missense Mutation | Lung Adenocarcinoma | Unknown |
| D2720H | Missense Mutation | Lung Adenocarcinoma | Unknown |
| L2780I | Missense Mutation | Uterine Endometrioid Carcinoma | Unknown |
| L2722M | Missense Mutation | Uterine Endometrioid Carcinoma | Unknown |
| L2722M | Missense Mutation | Uterine Endometrioid Carcinoma | Unknown |
| L2780R | Missense Mutation | Pancreatic Adenocarcinoma | Unknown |
| L2780R | Missense Mutation | Breast Invasive Ductal Carcinoma | Unknown |
| H2872R | Missense Mutation | Mucinous Adenocarcinoma of the Colon and Rectum | Unknown |
| Y2437C | Missense Mutation | Breast Invasive Ductal Carcinoma | Unknown |
| M1916V | Missense Mutation | Papillary Renal Cell Carcinoma | Unknown |
| R2526K | Missense Mutation | Cutaneous Melanoma | Unknown |
| R2763* | Nonsense Mutation | Tubular Stomach Adenocarcinoma | Likely Oncogenic |
| R1466Q | Missense Mutation | Stomach Adenocarcinoma | Predicted Oncogenic |
| R2598* | Nonsense Mutation | Uterine Endometrioid Carcinoma | Likely Oncogenic |
| R2598* | Nonsense Mutation | Tubular Stomach Adenocarcinoma | Likely Oncogenic |
| E770* | Nonsense Mutation | Lung Adenocarcinoma | Likely Oncogenic |
| E770* | Nonsense Mutation | Lung Adenocarcinoma | Likely Oncogenic |
| A2062V | Missense Mutation | Uterine Endometrioid Carcinoma | Likely Oncogenic |
| R2034* | Nonsense Mutation | Uterine Endometrioid Carcinoma | Likely Oncogenic |
| W1026Lfs*22 | Frame Shift Insertion | Bladder Urothelial Carcinoma | Likely Oncogenic |
| E2052* | Nonsense Mutation | Esophageal Squamous Cell Carcinoma | Likely Oncogenic |
| E2039K | Missense Mutation | Colon Adenocarcinoma | Likely Oncogenic |
| R1466* | Nonsense Mutation | Rectal Adenocarcinoma | Likely Oncogenic |
| Q2730P | Missense Mutation | Papillary Renal Cell Carcinoma | Likely Oncogenic |
| D2708V | Missense Mutation | Intrahepatic Cholangiocarcinoma | Likely Oncogenic |
| R2453P | Missense Mutation | Prostate Adenocarcinoma | Unknown |
| G1998E | Missense Mutation | Breast Invasive Ductal Carcinoma | Unknown |
| Y1753C | Missense Mutation | Glioblastoma Multiforme | Unknown |
| G1672A | Missense Mutation | Prostate Adenocarcinoma | Unknown |
| V2937G | Missense Mutation | Prostate Adenocarcinoma | Unknown |
| V2937G | Missense Mutation | Cutaneous Melanoma | Unknown |
| F2686C | Missense Mutation | Diffuse Type Stomach Adenocarcinoma | Unknown |
| G2695del | In Frame Deletion | Uterine Endometrioid Carcinoma | Unknown |
| G2695del | In Frame Deletion | Lung Adenocarcinoma | Unknown |
| G2695del | In Frame Deletion | Colon Adenocarcinoma | Unknown |
| R2459P | Missense Mutation | Lung Adenocarcinoma | Unknown |
| T939S | Missense Mutation | Lung Adenocarcinoma | Unknown |
| G1663V | Missense Mutation | Lung Adenocarcinoma | Unknown |
| L259I | Missense Mutation | Uterine Endometrioid Carcinoma | Unknown |
| T2853M | Missense Mutation | Uterine Endometrioid Carcinoma | Unknown |
| T2853M | Missense Mutation | Chromophobe Renal Cell Carcinoma | Unknown |
| F2140C | Missense Mutation | Uterine Endometrioid Carcinoma | Unknown |
| S978A | Missense Mutation | Uterine Endometrioid Carcinoma | Unknown |
| F1247L | Missense Mutation | Uterine Endometrioid Carcinoma | Unknown |
| L259F | Missense Mutation | Bladder Urothelial Carcinoma | Unknown |
| G558V | Missense Mutation | Bladder Urothelial Carcinoma | Unknown |
| T2853R | Missense Mutation | Renal Clear Cell Carcinoma | Unknown |
| G1663C | Missense Mutation | Lung Squamous Cell Carcinoma | Unknown |
| G1663C | Missense Mutation | Lung Squamous Cell Carcinoma | Unknown |
| R1973G | Missense Mutation | Lung Adenocarcinoma | Unknown |
| M349I | Missense Mutation | Esophageal Squamous Cell Carcinoma | Unknown |
| L2416P | Missense Mutation | Leiomyosarcoma | Unknown |
| F2140V | Missense Mutation | Colon Adenocarcinoma | Unknown |
| G2891R | Missense Mutation | Rectal Adenocarcinoma | Unknown |
| R2459C | Missense Mutation | Pancreatic Adenocarcinoma | Unknown |
| S978P | Missense Mutation | Diffuse Large B-Cell Lymphoma | Unknown |
| S707P | Missense Mutation | Diffuse Large B-Cell Lymphoma | Unknown |
| S707P | Missense Mutation | Diffuse Large B-Cell Lymphoma | Unknown |
| R248* | Nonsense Mutation | Lung Adenocarcinoma | Likely Oncogenic |
| R1575Yfs*10 | Frame Shift Deletion | Breast Invasive Ductal Carcinoma | Likely Oncogenic |
| S214Pfs*16 | Frame Shift Deletion | Stomach Adenocarcinoma | Likely Oncogenic |
| S214Pfs*16 | Frame Shift Deletion | Tubular Stomach Adenocarcinoma | Likely Oncogenic |
| K2811Sfs*46 | Frame Shift Deletion | Stomach Adenocarcinoma | Likely Oncogenic |
| K2811Sfs*46 | Frame Shift Deletion | Stomach Adenocarcinoma | Likely Oncogenic |
| R2849* | Nonsense Mutation | Uterine Endometrioid Carcinoma | Likely Oncogenic |
| Q1500* | Nonsense Mutation | Stomach Adenocarcinoma | Likely Oncogenic |
| Q2971* | Nonsense Mutation | Mucinous Stomach Adenocarcinoma | Likely Oncogenic |
| G204* | Nonsense Mutation | Stomach Adenocarcinoma | Likely Oncogenic |
| K2811Vfs*3 | Frame Shift Deletion | Tubular Stomach Adenocarcinoma | Likely Oncogenic |
| E958* | Nonsense Mutation | Lung Adenocarcinoma | Likely Oncogenic |
| E1892* | Nonsense Mutation | Lung Adenocarcinoma | Likely Oncogenic |
| R2263* | Nonsense Mutation | Lung Adenocarcinoma | Likely Oncogenic |
| I279Yfs*2 | Frame Shift Deletion | Lung Adenocarcinoma | Likely Oncogenic |
| Q2641* | Nonsense Mutation | Lung Adenocarcinoma | Likely Oncogenic |
| E1979* | Nonsense Mutation | Lung Adenocarcinoma | Likely Oncogenic |
| E1979* | Nonsense Mutation | Lung Adenocarcinoma | Likely Oncogenic |
| E3015* | Nonsense Mutation | Lung Adenocarcinoma | Likely Oncogenic |
| E3015* | Nonsense Mutation | Lung Adenocarcinoma | Likely Oncogenic |
| S1487* | Nonsense Mutation | Lung Adenocarcinoma | Likely Oncogenic |
| S1487* | Nonsense Mutation | Lung Adenocarcinoma | Likely Oncogenic |
| Q628* | Nonsense Mutation | Lung Adenocarcinoma | Likely Oncogenic |
| Q628* | Nonsense Mutation | Lung Adenocarcinoma | Likely Oncogenic |
| K1196* | Nonsense Mutation | Lung Adenocarcinoma | Likely Oncogenic |
| K1196* | Nonsense Mutation | Lung Adenocarcinoma | Likely Oncogenic |
| R1437* | Nonsense Mutation | Lung Adenocarcinoma | Likely Oncogenic |
| R1437* | Nonsense Mutation | Lung Adenocarcinoma | Likely Oncogenic |
| E1783* | Nonsense Mutation | Uterine Endometrioid Carcinoma | Likely Oncogenic |
| S719* | Nonsense Mutation | Uterine Endometrioid Carcinoma | Likely Oncogenic |
| E390* | Nonsense Mutation | Uterine Endometrioid Carcinoma | Likely Oncogenic |
| Q1946Vfs*18 | Frame Shift Deletion | Uterine Endometrioid Carcinoma | Likely Oncogenic |
| S131* | Nonsense Mutation | Uterine Endometrioid Carcinoma | Likely Oncogenic |
| E2139Ifs*6 | Frame Shift Deletion | Uterine Endometrioid Carcinoma | Likely Oncogenic |
| S2685* | Nonsense Mutation | Uterine Endometrioid Carcinoma | Likely Oncogenic |
| X2191_splice | Splice Site | Bladder Urothelial Carcinoma | Likely Oncogenic |
| E3022* | Nonsense Mutation | Bladder Urothelial Carcinoma | Likely Oncogenic |
| R2580* | Nonsense Mutation | Renal Clear Cell Carcinoma | Likely Oncogenic |
| K129* | Nonsense Mutation | Renal Clear Cell Carcinoma | Likely Oncogenic |
| R2419* | Nonsense Mutation | Renal Clear Cell Carcinoma | Likely Oncogenic |
| T2640Lfs*6 | Frame Shift Deletion | Renal Clear Cell Carcinoma | Likely Oncogenic |
| L427* | Nonsense Mutation | Lung Adenocarcinoma | Likely Oncogenic |
| X1332_splice | Splice Site | Lung Adenocarcinoma | Likely Oncogenic |
| A59S | Missense Mutation | Breast Invasive Lobular Carcinoma | Likely Oncogenic |
| L2452P | Missense Mutation | Papillary Thyroid Cancer | Likely Oncogenic |
| S214Ffs*40 | Frame Shift Insertion | Stomach Adenocarcinoma | Likely Oncogenic |
| Y137Tfs*16 | Frame Shift Deletion | Breast Invasive Ductal Carcinoma | Likely Oncogenic |
| W2769* | Nonsense Mutation | Colon Adenocarcinoma | Likely Oncogenic |
| G2425Vfs*15 | Frame Shift Deletion | Colon Adenocarcinoma | Likely Oncogenic |
| C1838Vfs*8 | Frame Shift Deletion | Colon Adenocarcinoma | Likely Oncogenic |
| E1822* | Nonsense Mutation | Colon Adenocarcinoma | Likely Oncogenic |
| E2676* | Nonsense Mutation | Colon Adenocarcinoma | Likely Oncogenic |
| E1666* | Nonsense Mutation | Colon Adenocarcinoma | Likely Oncogenic |
| Q2414* | Nonsense Mutation | Serous Ovarian Cancer | Likely Oncogenic |
| Q2269* | Nonsense Mutation | Breast Invasive Ductal Carcinoma | Likely Oncogenic |
| X1332_splice | Splice Site | Uterine Endometrioid Carcinoma | Likely Oncogenic |
| Q2433* | Nonsense Mutation | Cutaneous Melanoma | Likely Oncogenic |
| P2665N | Missense Mutation | Lung Adenocarcinoma | Unknown |
| N796T | Missense Mutation | Breast Invasive Ductal Carcinoma | Unknown |
| R1575L | Missense Mutation | Breast Invasive Ductal Carcinoma | Unknown |
| D1693N | Missense Mutation | Breast Invasive Ductal Carcinoma | Unknown |
| L2952F | Missense Mutation | Prostate Adenocarcinoma | Unknown |
| L1936S | Missense Mutation | Prostate Adenocarcinoma | Unknown |
| H1568L | Missense Mutation | Prostate Adenocarcinoma | Unknown |
| G2786S | Missense Mutation | Prostate Adenocarcinoma | Unknown |
| E586D | Missense Mutation | Esophageal Squamous Cell Carcinoma | Unknown |
| P2353H | Missense Mutation | Pancreatic Adenocarcinoma | Unknown |
| F2839L | Missense Mutation | Mucinous Adenocarcinoma of the Colon and Rectum | Unknown |
| I1659M | Missense Mutation | Stomach Adenocarcinoma | Unknown |
| Y2398C | Missense Mutation | Stomach Adenocarcinoma | Unknown |
| L1561P | Missense Mutation | Stomach Adenocarcinoma | Unknown |
| W2491L | Missense Mutation | Stomach Adenocarcinoma | Unknown |
| R2790K | Missense Mutation | Stomach Adenocarcinoma | Unknown |
| A244V | Missense Mutation | Stomach Adenocarcinoma | Unknown |
| W1461C | Missense Mutation | Stomach Adenocarcinoma | Unknown |
| R1095G | Missense Mutation | Stomach Adenocarcinoma | Unknown |
| F168L | Missense Mutation | Tubular Stomach Adenocarcinoma | Unknown |
| E871K | Missense Mutation | Tubular Stomach Adenocarcinoma | Unknown |
| F2410L | Missense Mutation | Stomach Adenocarcinoma | Unknown |
| N2567S | Missense Mutation | Stomach Adenocarcinoma | Unknown |
| L413I | Missense Mutation | Tubular Stomach Adenocarcinoma | Unknown |
| L2307F | Missense Mutation | Lung Adenocarcinoma | Unknown |
| W2845C | Missense Mutation | Follicular Thyroid Cancer | Unknown |
| W412C | Missense Mutation | Head and Neck Squamous Cell Carcinoma | Unknown |
| N859I | Missense Mutation | Lung Adenocarcinoma | Unknown |
| I2432S | Missense Mutation | Lung Adenocarcinoma | Unknown |
| E1182D | Missense Mutation | Lung Adenocarcinoma | Unknown |
| W412L | Missense Mutation | Lung Adenocarcinoma | Unknown |
| F1381C | Missense Mutation | Lung Adenocarcinoma | Unknown |
| F1381C | Missense Mutation | Lung Adenocarcinoma | Unknown |
| L898V | Missense Mutation | Lung Adenocarcinoma | Unknown |
| L898V | Missense Mutation | Lung Adenocarcinoma | Unknown |
| D841Y | Missense Mutation | Lung Adenocarcinoma | Unknown |
| D841Y | Missense Mutation | Lung Adenocarcinoma | Unknown |
| D2959H | Missense Mutation | Lung Adenocarcinoma | Unknown |
| D2959H | Missense Mutation | Lung Adenocarcinoma | Unknown |
| W3052C | Missense Mutation | Lung Adenocarcinoma | Unknown |
| W3052C | Missense Mutation | Lung Adenocarcinoma | Unknown |
| E2139D | Missense Mutation | Uterine Endometrioid Carcinoma | Unknown |
| I2683M | Missense Mutation | Uterine Endometrioid Carcinoma | Unknown |
| S367F | Missense Mutation | Uterine Endometrioid Carcinoma | Unknown |
| K1289N | Missense Mutation | Uterine Endometrioid Carcinoma | Unknown |
| P80S | Missense Mutation | Uterine Endometrioid Carcinoma | Unknown |
| L100W | Missense Mutation | Uterine Endometrioid Carcinoma | Unknown |
| L1046R | Missense Mutation | Uterine Endometrioid Carcinoma | Unknown |
| D351Y | Missense Mutation | Uterine Endometrioid Carcinoma | Unknown |
| D360Y | Missense Mutation | Uterine Endometrioid Carcinoma | Unknown |
| I487M | Missense Mutation | Uterine Endometrioid Carcinoma | Unknown |
| D317G | Missense Mutation | Uterine Endometrioid Carcinoma | Unknown |
| K2363N | Missense Mutation | Uterine Endometrioid Carcinoma | Unknown |
| P631S | Missense Mutation | Uterine Endometrioid Carcinoma | Unknown |
| L2561M | Missense Mutation | Uterine Endometrioid Carcinoma | Unknown |
| N2106K | Missense Mutation | Uterine Endometrioid Carcinoma | Unknown |
| L2866I | Missense Mutation | Uterine Endometrioid Carcinoma | Unknown |
| F1683V | Missense Mutation | Uterine Endometrioid Carcinoma | Unknown |
| K482Q | Missense Mutation | Uterine Endometrioid Carcinoma | Unknown |
| M1644I | Missense Mutation | Uterine Serous Carcinoma/Uterine Papillary Serous Carcinoma | Unknown |
| L2345F | Missense Mutation | Uterine Endometrioid Carcinoma | Unknown |
| G1456D | Missense Mutation | Bladder Urothelial Carcinoma | Unknown |
| E158Q | Missense Mutation | Bladder Urothelial Carcinoma | Unknown |
| E2815K | Missense Mutation | Bladder Urothelial Carcinoma | Unknown |
| Q3000H | Missense Mutation | Bladder Urothelial Carcinoma | Unknown |
| E3007K | Missense Mutation | Bladder Urothelial Carcinoma | Unknown |
| E2094K | Missense Mutation | Bladder Urothelial Carcinoma | Unknown |
| A749T | Missense Mutation | Renal Clear Cell Carcinoma | Unknown |
| T2264I | Missense Mutation | Renal Clear Cell Carcinoma | Unknown |
| K3004E | Missense Mutation | Renal Clear Cell Carcinoma | Unknown |
| G2718R | Missense Mutation | Renal Clear Cell Carcinoma | Unknown |
| V1678G | Missense Mutation | Renal Clear Cell Carcinoma | Unknown |
| N2494Y | Missense Mutation | Renal Clear Cell Carcinoma | Unknown |
| I1999V | Missense Mutation | Glioblastoma Multiforme | Unknown |
| T1871I | Missense Mutation | Lung Squamous Cell Carcinoma | Unknown |
| T1871I | Missense Mutation | Lung Squamous Cell Carcinoma | Unknown |
| L1675F | Missense Mutation | Lung Squamous Cell Carcinoma | Unknown |
| L1675F | Missense Mutation | Lung Squamous Cell Carcinoma | Unknown |
| D2987Y | Missense Mutation | Lung Squamous Cell Carcinoma | Unknown |
| D2987Y | Missense Mutation | Lung Squamous Cell Carcinoma | Unknown |
| G2897S | Missense Mutation | Lung Squamous Cell Carcinoma | Unknown |
| G2897S | Missense Mutation | Lung Squamous Cell Carcinoma | Unknown |
| K1323E | Missense Mutation | Lung Squamous Cell Carcinoma | Unknown |
| K1323E | Missense Mutation | Lung Squamous Cell Carcinoma | Unknown |
| D406N | Missense Mutation | Lung Squamous Cell Carcinoma | Unknown |
| D406N | Missense Mutation | Lung Squamous Cell Carcinoma | Unknown |
| K2413Q | Missense Mutation | Lung Adenocarcinoma | Unknown |
| M563T | Missense Mutation | Lung Adenocarcinoma | Unknown |
| A295P | Missense Mutation | Lung Adenocarcinoma | Unknown |
| G2043V | Missense Mutation | Lung Adenocarcinoma | Unknown |
| E2444K | Missense Mutation | Lung Adenocarcinoma | Unknown |
| D2650E | Missense Mutation | Lung Squamous Cell Carcinoma | Unknown |
| L2715R | Missense Mutation | Lung Adenocarcinoma | Unknown |
| E2814K | Missense Mutation | Breast Invasive Lobular Carcinoma | Unknown |
| E642K | Missense Mutation | Breast Invasive Ductal Carcinoma | Unknown |
| L2132V | Missense Mutation | Papillary Thyroid Cancer | Unknown |
| T2228I | Missense Mutation | Papillary Thyroid Cancer | Unknown |
| G2694R | Missense Mutation | Prostate Adenocarcinoma | Unknown |
| M1644I | Missense Mutation | Esophageal Adenocarcinoma | Unknown |
| D2016H | Missense Mutation | Esophageal Squamous Cell Carcinoma | Unknown |
| G335E | Missense Mutation | Signet Ring Cell Carcinoma of the Stomach | Unknown |
| T1880R | Missense Mutation | Stomach Adenocarcinoma | Unknown |
| S378R | Missense Mutation | Breast Invasive Ductal Carcinoma | Unknown |
| T2771I | Missense Mutation | Mucinous Adenocarcinoma of the Colon and Rectum | Unknown |
| S614N | Missense Mutation | Colon Adenocarcinoma | Unknown |
| Q1117P | Missense Mutation | Colon Adenocarcinoma | Unknown |
| R1489H | Missense Mutation | Colon Adenocarcinoma | Unknown |
| Q499E | Missense Mutation | Colon Adenocarcinoma | Unknown |
| A920V | Missense Mutation | Colon Adenocarcinoma | Unknown |
| N2435K | Missense Mutation | Colon Adenocarcinoma | Unknown |
| R2060H | Missense Mutation | Mucinous Adenocarcinoma of the Colon and Rectum | Unknown |
| L822V | Missense Mutation | Colon Adenocarcinoma | Unknown |
| R2598Q | Missense Mutation | Colon Adenocarcinoma | Unknown |
| K477N | Missense Mutation | Mucinous Adenocarcinoma of the Colon and Rectum | Unknown |
| L2077I | Missense Mutation | Mucinous Adenocarcinoma of the Colon and Rectum | Unknown |
| P2353T | Missense Mutation | Colon Adenocarcinoma | Unknown |
| A1024T | Missense Mutation | Colon Adenocarcinoma | Unknown |
| L2557V | Missense Mutation | Rectal Adenocarcinoma | Unknown |
| L942I | Missense Mutation | Rectal Adenocarcinoma | Unknown |
| R1150I | Missense Mutation | Rectal Adenocarcinoma | Unknown |
| L1217M | Missense Mutation | Rectal Adenocarcinoma | Unknown |
| L2147I | Missense Mutation | Rectal Adenocarcinoma | Unknown |
| L2251I | Missense Mutation | Rectal Adenocarcinoma | Unknown |
| D2507N | Missense Mutation | Serous Ovarian Cancer | Unknown |
| L243S | Missense Mutation | Serous Ovarian Cancer | Unknown |
| V716L | Missense Mutation | Breast Invasive Ductal Carcinoma | Unknown |
| A823T | Missense Mutation | Esophageal Squamous Cell Carcinoma | Unknown |
| L2623F | Missense Mutation | Cutaneous Melanoma | Unknown |
| P2759S | Missense Mutation | Cutaneous Melanoma | Unknown |
| S2168L | Missense Mutation | Cutaneous Melanoma | Unknown |
| H290Y | Missense Mutation | Cutaneous Melanoma | Unknown |
| C430S | Missense Mutation | Cutaneous Melanoma | Unknown |
| R1312K | Missense Mutation | Cutaneous Melanoma | Unknown |
| L2952I | Missense Mutation | Uterine Carcinosarcoma/Uterine Malignant Mixed Mullerian Tumor | Unknown |
| Q1331R | Missense Mutation | Esophageal Adenocarcinoma | Unknown |
| V462A | Missense Mutation | Cutaneous Melanoma | Unknown |
| D2650A | Missense Mutation | Breast Invasive Lobular Carcinoma | Unknown |
| X2032_splice | Splice Site | Breast Mixed Ductal and Lobular Carcinoma | Likely Oncogenic |
| X1331_splice | Splice Site | Papillary Renal Cell Carcinoma | Likely Oncogenic |
| N1094Dfs*14 | Frame Shift Deletion | Glioblastoma Multiforme | Likely Oncogenic |
| R23* | Nonsense Mutation | Adrenocortical Carcinoma | Likely Oncogenic |
| W484* | Frame Shift Deletion | Prostate Adenocarcinoma | Likely Oncogenic |
| Y1938Ifs*2 | Frame Shift Deletion | Prostate Adenocarcinoma | Likely Oncogenic |
| I1581Nfs*5 | Frame Shift Insertion | Stomach Adenocarcinoma | Likely Oncogenic |
| Q441Afs*45 | Frame Shift Deletion | Stomach Adenocarcinoma | Likely Oncogenic |
| S1455Vfs*3 | Frame Shift Deletion | Stomach Adenocarcinoma | Likely Oncogenic |
| K1904Nfs*13 | Frame Shift Deletion | Stomach Adenocarcinoma | Likely Oncogenic |
| X2151_splice | Splice Site | Mucinous Stomach Adenocarcinoma | Likely Oncogenic |
| T1745Dfs*4 | Frame Shift Insertion | Tubular Stomach Adenocarcinoma | Likely Oncogenic |
| K2749N | Missense Mutation | Head and Neck Squamous Cell Carcinoma | Likely Oncogenic |
| X2671_splice | Splice Site | Lung Adenocarcinoma | Likely Oncogenic |
| Q1225* | Nonsense Mutation | Lung Adenocarcinoma | Likely Oncogenic |
| Q1225* | Nonsense Mutation | Lung Adenocarcinoma | Likely Oncogenic |
| S751Ffs*14 | Frame Shift Insertion | Lung Adenocarcinoma | Likely Oncogenic |
| S751Ffs*14 | Frame Shift Insertion | Lung Adenocarcinoma | Likely Oncogenic |
| X355_splice | Splice Site | Lung Adenocarcinoma | Likely Oncogenic |
| X355_splice | Splice Site | Lung Adenocarcinoma | Likely Oncogenic |
| L2006* | Frame Shift Deletion | Lung Adenocarcinoma | Likely Oncogenic |
| L2006* | Frame Shift Deletion | Lung Adenocarcinoma | Likely Oncogenic |
| T1029Qfs*10 | Frame Shift Deletion | Lung Adenocarcinoma | Likely Oncogenic |
| T1029Qfs*10 | Frame Shift Deletion | Lung Adenocarcinoma | Likely Oncogenic |
| E2272* | Nonsense Mutation | Bladder Urothelial Carcinoma | Likely Oncogenic |
| K750Sfs*3 | Frame Shift Deletion | Renal Clear Cell Carcinoma | Likely Oncogenic |
| E2409Nfs*9 | Frame Shift Deletion | Glioblastoma Multiforme | Likely Oncogenic |
| X25_splice | Splice Site | Lung Squamous Cell Carcinoma | Likely Oncogenic |
| X25_splice | Splice Site | Lung Squamous Cell Carcinoma | Likely Oncogenic |
| L1347* | Nonsense Mutation | Pancreatic Adenocarcinoma | Likely Oncogenic |
| S2882* | Nonsense Mutation | Lung Squamous Cell Carcinoma | Likely Oncogenic |
| I1525Nfs*6 | Frame Shift Insertion | Lung Squamous Cell Carcinoma | Likely Oncogenic |
| X1052_splice | Splice Site | Lung Adenocarcinoma | Likely Oncogenic |
| C819Lfs*4 | Frame Shift Deletion | Lung Adenocarcinoma | Likely Oncogenic |
| Q65* | Nonsense Mutation | Lung Squamous Cell Carcinoma | Likely Oncogenic |
| L210Wfs*20 | Frame Shift Deletion | Lung Adenocarcinoma | Likely Oncogenic |
| Q1116Sfs*10 | Frame Shift Deletion | Lung Squamous Cell Carcinoma | Likely Oncogenic |
| Q2277* | Nonsense Mutation | Lung Squamous Cell Carcinoma | Likely Oncogenic |
| L663Ffs*42 | Frame Shift Insertion | Breast Invasive Ductal Carcinoma | Likely Oncogenic |
| T1908Kfs*9 | Frame Shift Deletion | Papillary Thyroid Cancer | Likely Oncogenic |
| S1403Afs*3 | Frame Shift Deletion | Leiomyosarcoma | Likely Oncogenic |
| S1403Afs*3 | Frame Shift Deletion | Stomach Adenocarcinoma | Likely Oncogenic |
| E1277* | Nonsense Mutation | Esophageal Adenocarcinoma | Likely Oncogenic |
| S698* | Frame Shift Deletion | Prostate Adenocarcinoma | Likely Oncogenic |
| C1396* | Frame Shift Deletion | Prostate Adenocarcinoma | Likely Oncogenic |
| E343Rfs*5 | Frame Shift Insertion | Stomach Adenocarcinoma | Likely Oncogenic |
| Q499* | Nonsense Mutation | Mucinous Stomach Adenocarcinoma | Likely Oncogenic |
| T1399Pfs*3 | Frame Shift Deletion | Diffuse Type Stomach Adenocarcinoma | Likely Oncogenic |
| R329Kfs*8 | Frame Shift Deletion | Stomach Adenocarcinoma | Likely Oncogenic |
| T1011Kfs*7 | Frame Shift Deletion | Adrenocortical Carcinoma | Likely Oncogenic |
| X2003_splice | Splice Site | Uterine Endometrioid Carcinoma | Likely Oncogenic |
| X1833_splice | Splice Site | Uterine Endometrioid Carcinoma | Likely Oncogenic |
| X1051_splice | Splice Site | Papillary Renal Cell Carcinoma | Likely Oncogenic |
| G2765S | Missense Mutation | Papillary Renal Cell Carcinoma | Likely Oncogenic |
| X2929_splice | Splice Site | Cutaneous Melanoma | Likely Oncogenic |
| X2671_splice | Splice Site | Hepatocellular Carcinoma | Likely Oncogenic |
| S629Tfs*20 | Frame Shift Deletion | Adrenocortical Carcinoma | Likely Oncogenic |
| X2325_splice | Splice Site | Cutaneous Melanoma | Likely Oncogenic |
| W164fs | Frame Shift Insertion | Cutaneous Melanoma | Likely Oncogenic |
| X633_splice | Splice Site | Adrenocortical Carcinoma | Likely Oncogenic |
| E1776Kfs*17 | Frame Shift Deletion | Renal Clear Cell Carcinoma | Likely Oncogenic |
| E166* | Nonsense Mutation | Esophageal Adenocarcinoma | Likely Oncogenic |
| K300= | Splice Region | Intrahepatic Cholangiocarcinoma | Likely Oncogenic |
| L2890= | Splice Region | Intrahepatic Cholangiocarcinoma | Likely Oncogenic |
| I923* | Frame Shift Insertion | Lung Adenocarcinoma | Likely Oncogenic |
| R2461C | Missense Mutation | Chromophobe Renal Cell Carcinoma | Unknown |
| G724V | Missense Mutation | Chromophobe Renal Cell Carcinoma | Unknown |
| I2899M | Missense Mutation | Head and Neck Squamous Cell Carcinoma | Unknown |
| I1846N | Missense Mutation | Prostate Adenocarcinoma | Unknown |
| G2919S | Missense Mutation | Prostate Adenocarcinoma | Unknown |
| E347K | Missense Mutation | Esophageal Squamous Cell Carcinoma | Unknown |
| R2461P | Missense Mutation | Bladder Urothelial Carcinoma | Unknown |
| Y1248C | Missense Mutation | Head and Neck Squamous Cell Carcinoma | Unknown |
| D1053N | Missense Mutation | Head and Neck Squamous Cell Carcinoma | Unknown |
| I238V | Missense Mutation | Head and Neck Squamous Cell Carcinoma | Unknown |
| I1035V | Missense Mutation | Head and Neck Squamous Cell Carcinoma | Unknown |
| D2997N | Missense Mutation | Head and Neck Squamous Cell Carcinoma | Unknown |
| Q284P | Missense Mutation | Bladder Urothelial Carcinoma | Unknown |
| E2884Q | Missense Mutation | Bladder Urothelial Carcinoma | Unknown |
| I124V | Missense Mutation | Diffuse Large B-Cell Lymphoma | Unknown |
| N230I | Missense Mutation | Lung Squamous Cell Carcinoma | Unknown |
| C2931W | Missense Mutation | Lung Squamous Cell Carcinoma | Unknown |
| D2913H | Missense Mutation | Lung Squamous Cell Carcinoma | Unknown |
| D1373N | Missense Mutation | Lung Squamous Cell Carcinoma | Unknown |
| D1548H | Missense Mutation | Lung Squamous Cell Carcinoma | Unknown |
| K1440N | Missense Mutation | Lung Adenocarcinoma | Unknown |
| L1028V | Missense Mutation | Lung Squamous Cell Carcinoma | Unknown |
| K659N | Missense Mutation | Lung Adenocarcinoma | Unknown |
| I1022T | Missense Mutation | Lung Squamous Cell Carcinoma | Unknown |
| V2758L | Missense Mutation | Lung Squamous Cell Carcinoma | Unknown |
| E1707K | Missense Mutation | Lung Squamous Cell Carcinoma | Unknown |
| K2421N | Missense Mutation | Lung Adenocarcinoma | Unknown |
| N1431S | Missense Mutation | Lung Squamous Cell Carcinoma | Unknown |
| T2142A | Missense Mutation | Lung Squamous Cell Carcinoma | Unknown |
| S1684C | Missense Mutation | Lung Adenocarcinoma | Unknown |
| R2138T | Missense Mutation | Lung Squamous Cell Carcinoma | Unknown |
| Y2627C | Missense Mutation | Lung Adenocarcinoma | Unknown |
| S592N | Missense Mutation | Lung Squamous Cell Carcinoma | Unknown |
| D256H | Missense Mutation | Lung Adenocarcinoma | Unknown |
| E649D | Missense Mutation | Lung Adenocarcinoma | Unknown |
| G956E | Missense Mutation | Lung Adenocarcinoma | Unknown |
| G2772E | Missense Mutation | Lung Adenocarcinoma | Unknown |
| E1325Q | Missense Mutation | Lung Adenocarcinoma | Unknown |
| R981C | Missense Mutation | Mucinous Stomach Adenocarcinoma | Unknown |
| P829S | Missense Mutation | Esophageal Adenocarcinoma | Unknown |
| S1673R | Missense Mutation | Esophageal Adenocarcinoma | Unknown |
| I2278V | Missense Mutation | Esophageal Squamous Cell Carcinoma | Unknown |
| P1680H | Missense Mutation | Esophageal Squamous Cell Carcinoma | Unknown |
| E2768Q | Missense Mutation | Esophageal Adenocarcinoma | Unknown |
| L1078R | Missense Mutation | Esophageal Adenocarcinoma | Unknown |
| R2849Q | Missense Mutation | Esophageal Squamous Cell Carcinoma | Unknown |
| L1115P | Missense Mutation | Esophageal Adenocarcinoma | Unknown |
| T1558M | Missense Mutation | Esophageal Adenocarcinoma | Unknown |
| S496I | Missense Mutation | Esophageal Adenocarcinoma | Unknown |
| A502S | Missense Mutation | Esophageal Squamous Cell Carcinoma | Unknown |
| R1768G | Missense Mutation | Hepatocellular Carcinoma | Unknown |
| L1078V | Missense Mutation | Prostate Adenocarcinoma | Unknown |
| V916M | Missense Mutation | Prostate Adenocarcinoma | Unknown |
| F1036_V1038del | In Frame Deletion | Prostate Adenocarcinoma | Unknown |
| E22G | Missense Mutation | Diffuse Type Stomach Adenocarcinoma | Unknown |
| V1190L | Missense Mutation | Mucinous Stomach Adenocarcinoma | Unknown |
| R1882Q | Missense Mutation | Diffuse Type Stomach Adenocarcinoma | Unknown |
| D2090E | Missense Mutation | Mucinous Stomach Adenocarcinoma | Unknown |
| E365D | Missense Mutation | Stomach Adenocarcinoma | Unknown |
| S1589L | Missense Mutation | Undifferentiated Pleomorphic Sarcoma/Malignant Fibrous Histiocytoma/High-Grade Spindle Cell Sarcoma | Unknown |
| L400I | Missense Mutation | Myxofibrosarcoma | Unknown |
| L1617R | Missense Mutation | Uterine Carcinosarcoma/Uterine Malignant Mixed Mullerian Tumor | Unknown |
| I882V | Missense Mutation | Embryonal Carcinoma | Unknown |
| Q501K | Missense Mutation | Intrahepatic Cholangiocarcinoma | Unknown |
| S2581T | Missense Mutation | Diffuse Large B-Cell Lymphoma | Unknown |
| E2932K | Missense Mutation | Head and Neck Squamous Cell Carcinoma | Unknown |
| D2988N | Missense Mutation | Head and Neck Squamous Cell Carcinoma | Unknown |
| V1506M | Missense Mutation | Head and Neck Squamous Cell Carcinoma | Unknown |
| E2238Q | Missense Mutation | Head and Neck Squamous Cell Carcinoma | Unknown |
| T2333I | Missense Mutation | Head and Neck Squamous Cell Carcinoma | Unknown |
| T2754A | Missense Mutation | Head and Neck Squamous Cell Carcinoma | Unknown |
| S681C | Missense Mutation | Hepatocellular Carcinoma | Unknown |
| L174R | Missense Mutation | Hepatocellular Carcinoma | Unknown |
| C219S | Missense Mutation | Hepatocellular Carcinoma | Unknown |
| S867T | Missense Mutation | Hepatocellular Carcinoma | Unknown |
| L2885H | Missense Mutation | Hepatocellular Carcinoma | Unknown |
| Q2729H | Missense Mutation | Hepatocellular Carcinoma | Unknown |
| T2934I | Missense Mutation | Pancreatic Adenocarcinoma | Unknown |
| R1898Q | Missense Mutation | Pancreatic Adenocarcinoma | Unknown |
| T2983S | Missense Mutation | Cutaneous Melanoma | Unknown |
| A1812G | Missense Mutation | Pleural Mesothelioma, Epithelioid Type | Unknown |
| S3027I | Missense Mutation | Adrenocortical Carcinoma | Unknown |
| H2099Y | Missense Mutation | Melanoma | Unknown |
| S1403I | Missense Mutation | Leiomyosarcoma | Unknown |
| L167F | Missense Mutation | Pleural Mesothelioma, Biphasic Type | Unknown |
| R250P | Missense Mutation | Cutaneous Melanoma | Unknown |
| K1192R | Missense Mutation | Intrahepatic Cholangiocarcinoma | Unknown |
| W484C | Missense Mutation | Lung Adenocarcinoma | Unknown |
| M855L | Missense Mutation | Lung Squamous Cell Carcinoma | Unknown |
| E1514Q | Missense Mutation | Lung Squamous Cell Carcinoma | Unknown |
|  |  |  |  |
| **BARD1 Mutation** | **Mutation Type** | **Cancer Type** | **OncoKB Analysis** |
| R21L | Missense Mutation | Cutaneous Melanoma | Unknown |
| R43Afs*15 | Frame Shift Deletion | Head and Neck Squamous Cell Carcinoma | Likely Oncogenic |
| E67K | Missense Mutation | Lung Adenocarcinoma | Unknown |
| R99K | Missense Mutation | Diffuse Large B-Cell Lymphoma | Likely Oncogenic |
| M104I | Missense Mutation | Renal Clear Cell Carcinoma | Unknown |
| S131Ffs*3 | Frame Shift Deletion | Esophageal Squamous Cell Carcinoma | Likely Oncogenic |
| S131Vfs*28 | Frame Shift Deletion | Head and Neck Squamous Cell Carcinoma | Likely Oncogenic |
| K140N | Missense Mutation | Uterine Endometrioid Carcinoma | Unknown |
| K140N | Missense Mutation | Rectal Adenocarcinoma | Unknown |
| S142* | Nonsense Mutation | Uterine Endometrioid Carcinoma | Likely Oncogenic |
| S142* | Nonsense Mutation | Esophageal Squamous Cell Carcinoma | Likely Oncogenic |
| Q164* | Nonsense Mutation | Uterine Endometrioid Carcinoma | Likely Oncogenic |
| T165S | Missense Mutation | Lung Adenocarcinoma | Unknown |
| P167T | Missense Mutation | Esophageal Adenocarcinoma | Unknown |
| D172Mfs*40 | Frame Shift Deletion | Stomach Adenocarcinoma | Likely Oncogenic |
| D172Mfs*40 | Frame Shift Deletion | Stomach Adenocarcinoma | Likely Oncogenic |
| S174I | Missense Mutation | Lung Adenocarcinoma | Unknown |
| V183L | Missense Mutation | Colon Adenocarcinoma | Unknown |
| S186N | Missense Mutation | Glioblastoma Multiforme | Unknown |
| D190Y | Missense Mutation | Uterine Endometrioid Carcinoma | Unknown |
| S192F | Missense Mutation | Lung Adenocarcinoma | Unknown |
| S192F | Missense Mutation | Lung Adenocarcinoma | Unknown |
| K197Rfs*15 | Frame Shift Deletion | Stomach Adenocarcinoma | Likely Oncogenic |
| A198V | Missense Mutation | Pancreatic Adenocarcinoma | Unknown |
| S202C | Missense Mutation | Bladder Urothelial Carcinoma | Unknown |
| K208Rfs*4 | Frame Shift Deletion | Stomach Adenocarcinoma | Likely Oncogenic |
| K208Rfs*4 | Frame Shift Deletion | Stomach Adenocarcinoma | Likely Oncogenic |
| K209Efs*5 | Frame Shift Insertion | Stomach Adenocarcinoma | Likely Oncogenic |
| E221D | Missense Mutation | Lung Adenocarcinoma | Unknown |
| E234V | Missense Mutation | Head and Neck Squamous Cell Carcinoma | Unknown |
| L239R | Missense Mutation | Uterine Endometrioid Carcinoma | Unknown |
| I254L | Missense Mutation | Lung Adenocarcinoma | Unknown |
| I254L | Missense Mutation | Lung Adenocarcinoma | Unknown |
| T267K | Missense Mutation | Esophageal Adenocarcinoma | Unknown |
| E268Q | Missense Mutation | Lung Squamous Cell Carcinoma | Unknown |
| P281S | Missense Mutation | Prostate Adenocarcinoma | Unknown |
| E287* | Nonsense Mutation | Rectal Adenocarcinoma | Likely Oncogenic |
| V298A | Missense Mutation | Uterine Endometrioid Carcinoma | Unknown |
| P300T | Missense Mutation | Head and Neck Squamous Cell Carcinoma | Unknown |
| S310C | Missense Mutation | Head and Neck Squamous Cell Carcinoma | Unknown |
| P315S | Missense Mutation | Prostate Adenocarcinoma | Unknown |
| K321Nfs*21 | Frame Shift Deletion | Stomach Adenocarcinoma | Likely Oncogenic |
| R322H | Missense Mutation | Lung Adenocarcinoma | Unknown |
| L341V | Missense Mutation | Lung Adenocarcinoma | Unknown |
| S344R | Missense Mutation | Lung Squamous Cell Carcinoma | Unknown |
| S344R | Missense Mutation | Lung Squamous Cell Carcinoma | Unknown |
| L359_P365del | In Frame Deletion | Renal Clear Cell Carcinoma | Unknown |
| P360L | Missense Mutation | Diffuse Type Stomach Adenocarcinoma | Unknown |
| T375A | Missense Mutation | Stomach Adenocarcinoma | Unknown |
| N380Tfs*20 | Frame Shift Deletion | Lung Adenocarcinoma | Likely Oncogenic |
| I388V | Missense Mutation | Head and Neck Squamous Cell Carcinoma | Unknown |
| T394A | Missense Mutation | Pancreatic Adenocarcinoma | Unknown |
| R406Q | Missense Mutation | Uterine Endometrioid Carcinoma | Unknown |
| P411H | Missense Mutation | Lung Squamous Cell Carcinoma | Unknown |
| P411S | Missense Mutation | Lentigo Maligna Melanoma | Unknown |
| A413T | Missense Mutation | Lung Squamous Cell Carcinoma | Unknown |
| A413T | Missense Mutation | Lung Squamous Cell Carcinoma | Unknown |
| A435V | Missense Mutation | Cutaneous Melanoma | Unknown |
| G439= | Splice Region | Uterine Endometrioid Carcinoma | Likely Oncogenic |
| X439_splice | Splice Site | Lung Adenocarcinoma | Likely Oncogenic |
| I441M | Missense Mutation | Colon Adenocarcinoma | Unknown |
| P454S | Missense Mutation | Lentigo Maligna Melanoma | Unknown |
| A460T | Missense Mutation | Colon Adenocarcinoma | Unknown |
| N470del | In Frame Deletion | Lung Adenocarcinoma | Unknown |
| L480S | Missense Mutation | Stomach Adenocarcinoma | Unknown |
| H483R | Missense Mutation | Lung Adenocarcinoma | Unknown |
| K484E | Missense Mutation | Head and Neck Squamous Cell Carcinoma | Unknown |
| T490P | Missense Mutation | Lung Adenocarcinoma | Unknown |
| V510A | Missense Mutation | Uterine Carcinosarcoma/Uterine Malignant Mixed Mullerian Tumor | Unknown |
| V523I | Missense Mutation | Renal Clear Cell Carcinoma | Unknown |
| P530L | Missense Mutation | Hepatocellular Carcinoma | Unknown |
| S538I | Missense Mutation | Head and Neck Squamous Cell Carcinoma | Unknown |
| S551L | Missense Mutation | Prostate Adenocarcinoma | Unknown |
| G574D | Missense Mutation | Oligoastrocytoma | Unknown |
| G576V | Missense Mutation | Lung Adenocarcinoma | Unknown |
| L585I | Missense Mutation | Pancreatic Adenocarcinoma | Unknown |
| K596Nfs*9 | Frame Shift Deletion | Diffuse Type Stomach Adenocarcinoma | Likely Oncogenic |
| X604_splice | Splice Site | Lung Squamous Cell Carcinoma | Likely Oncogenic |
| H606D | Missense Mutation | Bladder Urothelial Carcinoma | Unknown |
| H606N | Missense Mutation | Esophageal Adenocarcinoma | Unknown |
| A613Qfs*6 | Frame Shift Deletion | Glioblastoma Multiforme | Likely Oncogenic |
| L625I | Missense Mutation | Uterine Endometrioid Carcinoma | Unknown |
| R641Q | Missense Mutation | Uterine Endometrioid Carcinoma | Unknown |
| E648* | Nonsense Mutation | Lung Adenocarcinoma | Likely Oncogenic |
| E648* | Nonsense Mutation | Lung Adenocarcinoma | Likely Oncogenic |
| K650T | Missense Mutation | Renal Clear Cell Carcinoma | Unknown |
| S660C | Missense Mutation | Lung Squamous Cell Carcinoma | Unknown |
| S660R | Missense Mutation | Lung Squamous Cell Carcinoma | Unknown |
| S660R | Missense Mutation | Lung Squamous Cell Carcinoma | Unknown |
| L679F | Missense Mutation | Rectal Adenocarcinoma | Unknown |
| W680* | Nonsense Mutation | Colon Adenocarcinoma | Likely Oncogenic |
| G698D | Missense Mutation | Head and Neck Squamous Cell Carcinoma | Unknown |
| S704I | Missense Mutation | Lung Squamous Cell Carcinoma | Unknown |
| S704R | Missense Mutation | Papillary Renal Cell Carcinoma | Unknown |
| P707S | Missense Mutation | Tubular Stomach Adenocarcinoma | Unknown |
| A724V | Missense Mutation | Pheochromocytoma | Unknown |
| N744D | Missense Mutation | Tubular Stomach Adenocarcinoma | Unknown |
| G753D | Missense Mutation | Stomach Adenocarcinoma | Unknown |

| **BRCA1 Mutation** | **Mutation Type** | **Cancer Type** | **OncoKB Analysis** |
| --- | --- | --- | --- |
| A622V | Missense Mutation | Uterine Endometrioid Carcinoma | Unknown |
| A622V | Missense Mutation | Glioblastoma Multiforme | Unknown |
| P1749S | Missense Mutation | Cutaneous Melanoma | Likely Oncogenic |
| P1749S | Missense Mutation | Lung Adenocarcinoma | Likely Oncogenic |
| P1749S | Missense Mutation | Lung Adenocarcinoma | Likely Oncogenic |
| D1344H | Missense Mutation | Breast Invasive Ductal Carcinoma | Inconclusive |
| E1258G | Missense Mutation | Stomach Adenocarcinoma | Unknown |
| E1258D | Missense Mutation | Uterine Endometrioid Carcinoma | Unknown |
| L30F | Missense Mutation | Renal Clear Cell Carcinoma | Unknown |
| N1309I | Missense Mutation | Renal Clear Cell Carcinoma | Unknown |
| N1309H | Missense Mutation | Renal Clear Cell Carcinoma | Unknown |
| D1344N | Missense Mutation | Lung Squamous Cell Carcinoma | Unknown |
| D1344N | Missense Mutation | Lung Squamous Cell Carcinoma | Unknown |
| P871S | Missense Mutation | Lung Squamous Cell Carcinoma | Unknown |
| R1835* | Nonsense Mutation | Serous Ovarian Cancer | Likely Oncogenic |
| C47W | Missense Mutation | Serous Ovarian Cancer | Likely Oncogenic |
| P982L | Missense Mutation | Glioblastoma Multiforme | Unknown |
| E272K | Missense Mutation | Cutaneous Melanoma | Unknown |
| E143Q | Missense Mutation | Lung Adenocarcinoma | Unknown |
| R1649I | Missense Mutation | Lung Adenocarcinoma | Unknown |
| W321C | Missense Mutation | Lung Squamous Cell Carcinoma | Unknown |
| W321C | Missense Mutation | Lung Squamous Cell Carcinoma | Unknown |
| G778D | Missense Mutation | Lung Squamous Cell Carcinoma | Unknown |
| G778D | Missense Mutation | Lung Squamous Cell Carcinoma | Unknown |
| R1649T | Missense Mutation | Lung Adenocarcinoma | Unknown |
| K1667T | Missense Mutation | Intrahepatic Cholangiocarcinoma | Unknown |
| N1265Kfs*4 | Frame Shift Insertion | Serous Ovarian Cancer | Likely Oncogenic |
| S1217Rfs*21 | Frame Shift Insertion | Serous Ovarian Cancer | Likely Oncogenic |
| G1788V | Missense Mutation | Breast Invasive Ductal Carcinoma | Likely Oncogenic |
| D1692N | Missense Mutation | Stomach Adenocarcinoma | Likely Oncogenic |
| R1751* | Nonsense Mutation | Tubular Stomach Adenocarcinoma | Likely Oncogenic |
| E673* | Nonsense Mutation | Uterine Endometrioid Carcinoma | Likely Oncogenic |
| R1443* | Nonsense Mutation | Uterine Endometrioid Carcinoma | Likely Oncogenic |
| L1764I | Missense Mutation | Bladder Urothelial Carcinoma | Likely Oncogenic |
| Q94* | Nonsense Mutation | Lung Squamous Cell Carcinoma | Likely Oncogenic |
| Q94* | Nonsense Mutation | Lung Squamous Cell Carcinoma | Likely Oncogenic |
| K1160* | Nonsense Mutation | Lung Squamous Cell Carcinoma | Likely Oncogenic |
| K1160* | Nonsense Mutation | Lung Squamous Cell Carcinoma | Likely Oncogenic |
| G535* | Nonsense Mutation | Lung Adenocarcinoma | Likely Oncogenic |
| E720* | Nonsense Mutation | Breast Invasive Ductal Carcinoma | Likely Oncogenic |
| Q1538* | Nonsense Mutation | Serous Ovarian Cancer | Likely Oncogenic |
| G813Dfs*2 | Frame Shift Deletion | Serous Ovarian Cancer | Likely Oncogenic |
| G1710Efs*4 | Frame Shift Deletion | Serous Ovarian Cancer | Likely Oncogenic |
| E116* | Nonsense Mutation | Serous Ovarian Cancer | Likely Oncogenic |
| A521Qfs*11 | Frame Shift Deletion | Serous Ovarian Cancer | Likely Oncogenic |
| L431* | Nonsense Mutation | Serous Ovarian Cancer | Likely Oncogenic |
| Q202* | Nonsense Mutation | Cutaneous Melanoma | Likely Oncogenic |
| S1503* | Nonsense Mutation | Colon Adenocarcinoma | Likely Oncogenic |
| S988* | Nonsense Mutation | Lung Squamous Cell Carcinoma | Likely Oncogenic |
| G1422V | Missense Mutation | Lung Adenocarcinoma | Unknown |
| G1422V | Missense Mutation | Lung Adenocarcinoma | Unknown |
| P985L | Missense Mutation | Cutaneous Melanoma | Unknown |
| R1203Q | Missense Mutation | Glioblastoma Multiforme | Unknown |
| R979H | Missense Mutation | Glioblastoma Multiforme | Unknown |
| R1589C | Missense Mutation | Cutaneous Melanoma | Unknown |
| P832S | Missense Mutation | Melanoma | Unknown |
| R610M | Missense Mutation | Stomach Adenocarcinoma | Unknown |
| S1499F | Missense Mutation | Stomach Adenocarcinoma | Unknown |
| K893N | Missense Mutation | Tubular Stomach Adenocarcinoma | Unknown |
| R1028C | Missense Mutation | Stomach Adenocarcinoma | Unknown |
| R1397M | Missense Mutation | Stomach Adenocarcinoma | Unknown |
| K991Q | Missense Mutation | Stomach Adenocarcinoma | Unknown |
| P529S | Missense Mutation | Mucinous Stomach Adenocarcinoma | Unknown |
| E368G | Missense Mutation | Stomach Adenocarcinoma | Unknown |
| A887T | Missense Mutation | Tubular Stomach Adenocarcinoma | Unknown |
| E1250K | Missense Mutation | Diffuse Type Stomach Adenocarcinoma | Likely Neutral |
| A524T | Missense Mutation | Lung Adenocarcinoma | Unknown |
| V412L | Missense Mutation | Lung Adenocarcinoma | Unknown |
| V412L | Missense Mutation | Lung Adenocarcinoma | Unknown |
| G1350A | Missense Mutation | Lung Adenocarcinoma | Unknown |
| G1350A | Missense Mutation | Lung Adenocarcinoma | Unknown |
| K175N | Missense Mutation | Lung Adenocarcinoma | Unknown |
| K175N | Missense Mutation | Lung Adenocarcinoma | Unknown |
| T1548M | Missense Mutation | Lung Adenocarcinoma | Unknown |
| T1548M | Missense Mutation | Lung Adenocarcinoma | Unknown |
| L574I | Missense Mutation | Uterine Endometrioid Carcinoma | Unknown |
| L49M | Missense Mutation | Uterine Endometrioid Carcinoma | Unknown |
| K996Q | Missense Mutation | Uterine Endometrioid Carcinoma | Unknown |
| F461C | Missense Mutation | Uterine Endometrioid Carcinoma | Unknown |
| A942V | Missense Mutation | Uterine Endometrioid Carcinoma | Unknown |
| E1033K | Missense Mutation | Uterine Endometrioid Carcinoma | Unknown |
| C644W | Missense Mutation | Uterine Endometrioid Carcinoma | Unknown |
| G543C | Missense Mutation | Uterine Endometrioid Carcinoma | Unknown |
| K1207N | Missense Mutation | Uterine Endometrioid Carcinoma | Unknown |
| N1403S | Missense Mutation | Uterine Endometrioid Carcinoma | Unknown |
| E962A | Missense Mutation | Uterine Endometrioid Carcinoma | Unknown |
| S561Y | Missense Mutation | Uterine Endometrioid Carcinoma | Unknown |
| C903S | Missense Mutation | Bladder Urothelial Carcinoma | Unknown |
| E572K | Missense Mutation | Bladder Urothelial Carcinoma | Unknown |
| R446T | Missense Mutation | Bladder Urothelial Carcinoma | Unknown |
| P1831L | Missense Mutation | Renal Clear Cell Carcinoma | Unknown |
| T1399S | Missense Mutation | Renal Clear Cell Carcinoma | Unknown |
| V525A | Missense Mutation | Lung Squamous Cell Carcinoma | Unknown |
| V525A | Missense Mutation | Lung Squamous Cell Carcinoma | Unknown |
| R320T | Missense Mutation | Lung Squamous Cell Carcinoma | Unknown |
| R320T | Missense Mutation | Lung Squamous Cell Carcinoma | Unknown |
| V1665L | Missense Mutation | Lung Squamous Cell Carcinoma | Unknown |
| V1665L | Missense Mutation | Lung Squamous Cell Carcinoma | Unknown |
| E1115Q | Missense Mutation | Lung Squamous Cell Carcinoma | Unknown |
| E1115Q | Missense Mutation | Lung Squamous Cell Carcinoma | Unknown |
| E902Q | Missense Mutation | Lung Squamous Cell Carcinoma | Unknown |
| E902Q | Missense Mutation | Lung Squamous Cell Carcinoma | Unknown |
| E515V | Missense Mutation | Lung Squamous Cell Carcinoma | Unknown |
| E515V | Missense Mutation | Lung Squamous Cell Carcinoma | Unknown |
| E902K | Missense Mutation | Lung Squamous Cell Carcinoma | Unknown |
| E1527D | Missense Mutation | Lung Adenocarcinoma | Unknown |
| E418D | Missense Mutation | Lung Adenocarcinoma | Unknown |
| E9Q | Missense Mutation | Breast Invasive Ductal Carcinoma | Unknown |
| K50T | Missense Mutation | Breast Invasive Mixed Mucinous Carcinoma | Unknown |
| C644del | In Frame Deletion | Breast Invasive Ductal Carcinoma | Unknown |
| S1027I | Missense Mutation | Breast Invasive Ductal Carcinoma | Unknown |
| D366N | Missense Mutation | Breast Invasive Ductal Carcinoma | Unknown |
| V412I | Missense Mutation | Papillary Thyroid Cancer | Unknown |
| A524E | Missense Mutation | Esophageal Adenocarcinoma | Unknown |
| E489G | Missense Mutation | Colon Adenocarcinoma | Unknown |
| E907K | Missense Mutation | Colon Adenocarcinoma | Unknown |
| R170Q | Missense Mutation | Colon Adenocarcinoma | Likely Neutral |
| D821Y | Missense Mutation | Colon Adenocarcinoma | Unknown |
| E453D | Missense Mutation | Rectal Adenocarcinoma | Unknown |
| K223T | Missense Mutation | Esophageal Squamous Cell Carcinoma | Unknown |
| K175N | Missense Mutation | Head and Neck Squamous Cell Carcinoma | Unknown |
| L1098I | Missense Mutation | Cutaneous Melanoma | Unknown |
| G911R | Missense Mutation | Papillary Renal Cell Carcinoma | Unknown |
| E402K | Missense Mutation | Cutaneous Melanoma | Unknown |
| P875S | Missense Mutation | Cutaneous Melanoma | Unknown |
| H513Y | Missense Mutation | Cutaneous Melanoma | Unknown |
| P606L | Missense Mutation | Desmoplastic Melanoma | Unknown |
| E1214D | Missense Mutation | Melanoma | Unknown |
| S1423R | Missense Mutation | Embryonal Carcinoma | Unknown |
| X1559_splice | Splice Site | Metaplastic Breast Cancer | Likely Oncogenic |
| K339Rfs*2 | Frame Shift Deletion | Stomach Adenocarcinoma | Likely Oncogenic |
| K339Rfs*2 | Frame Shift Deletion | Stomach Adenocarcinoma | Likely Oncogenic |
| X1778_splice | Splice Site | Uterine Endometrioid Carcinoma | Likely Oncogenic |
| X1718_splice | Splice Site | Lung Squamous Cell Carcinoma | Likely Oncogenic |
| X1718_splice | Splice Site | Lung Squamous Cell Carcinoma | Likely Oncogenic |
| E1214* | Nonsense Mutation | Lung Adenocarcinoma | Likely Oncogenic |
| R1028Vfs*20 | Frame Shift Deletion | Lung Squamous Cell Carcinoma | Likely Oncogenic |
| E1257Gfs*9 | Frame Shift Deletion | Lung Squamous Cell Carcinoma | Likely Oncogenic |
| P1614Qfs*19 | Frame Shift Deletion | Breast Invasive Ductal Carcinoma | Likely Oncogenic |
| L1564Wfs*37 | Frame Shift Deletion | Esophageal Adenocarcinoma | Likely Oncogenic |
| T1691= | Splice Region | Diffuse Type Stomach Adenocarcinoma | Likely Oncogenic |
| Q139* | Nonsense Mutation | Breast Mixed Ductal and Lobular Carcinoma | Likely Oncogenic |
| X101_splice | Splice Site | Head and Neck Squamous Cell Carcinoma | Likely Oncogenic |
| V1234* | Frame Shift Deletion | Papillary Renal Cell Carcinoma | Likely Oncogenic |
| F1003* | Frame Shift Deletion | Cutaneous Melanoma | Likely Oncogenic |
| R1495G | Missense Mutation | Hepatocellular Carcinoma | Likely Oncogenic |
| K1671Nfs*6 | Frame Shift Deletion | Hepatocellular Carcinoma | Likely Oncogenic |
| K168Sfs*66 | Frame Shift Deletion | Hepatocellular Carcinoma | Likely Oncogenic |
| E1683* | Nonsense Mutation | Lung Adenocarcinoma | Likely Oncogenic |
| L1439F | Missense Mutation | Lung Squamous Cell Carcinoma | Unknown |
| E181D | Missense Mutation | Prostate Adenocarcinoma | Unknown |
| I456T | Missense Mutation | Lung Adenocarcinoma | Unknown |
| D853N | Missense Mutation | Head and Neck Squamous Cell Carcinoma | Unknown |
| S1241F | Missense Mutation | Head and Neck Squamous Cell Carcinoma | Unknown |
| H816Y | Missense Mutation | Head and Neck Squamous Cell Carcinoma | Unknown |
| G1492R | Missense Mutation | Head and Neck Squamous Cell Carcinoma | Unknown |
| E230Q | Missense Mutation | Head and Neck Squamous Cell Carcinoma | Unknown |
| D1505N | Missense Mutation | Head and Neck Squamous Cell Carcinoma | Unknown |
| R664G | Missense Mutation | Head and Neck Squamous Cell Carcinoma | Unknown |
| R1645M | Missense Mutation | Head and Neck Squamous Cell Carcinoma | Unknown |
| E1000Q | Missense Mutation | Lung Adenocarcinoma | Unknown |
| E1000Q | Missense Mutation | Lung Adenocarcinoma | Unknown |
| S1651F | Missense Mutation | Lung Squamous Cell Carcinoma | Likely Neutral |
| S889C | Missense Mutation | Lung Squamous Cell Carcinoma | Unknown |
| G1371V | Missense Mutation | Lung Adenocarcinoma | Unknown |
| E1682K | Missense Mutation | Lung Adenocarcinoma | Likely Neutral |
| Q1135L | Missense Mutation | Lung Squamous Cell Carcinoma | Unknown |
| V1654E | Missense Mutation | Lung Squamous Cell Carcinoma | Unknown |
| H1244N | Missense Mutation | Lung Squamous Cell Carcinoma | Unknown |
| S1466C | Missense Mutation | Lung Squamous Cell Carcinoma | Unknown |
| A224S | Missense Mutation | Lung Squamous Cell Carcinoma | Unknown |
| T1310I | Missense Mutation | Lung Adenocarcinoma | Unknown |
| R1645S | Missense Mutation | Lung Adenocarcinoma | Unknown |
| G707R | Missense Mutation | Lung Adenocarcinoma | Unknown |
| M48I | Missense Mutation | Lung Adenocarcinoma | Unknown |
| D96H | Missense Mutation | Breast Invasive Ductal Carcinoma | Unknown |
| R1737T | Missense Mutation | Head and Neck Squamous Cell Carcinoma | Unknown |
| S1434N | Missense Mutation | Diffuse Large B-Cell Lymphoma | Unknown |
| H1421N | Missense Mutation | Esophageal Squamous Cell Carcinoma | Unknown |
| E1013Q | Missense Mutation | Esophageal Adenocarcinoma | Unknown |
| A1669D | Missense Mutation | Esophageal Squamous Cell Carcinoma | Unknown |
| A1608T | Missense Mutation | Hepatocellular Carcinoma | Unknown |
| E1419K | Missense Mutation | Breast Invasive Lobular Carcinoma | Unknown |
| E673K | Missense Mutation | Diffuse Large B-Cell Lymphoma | Unknown |
| S184C | Missense Mutation | Head and Neck Squamous Cell Carcinoma | Unknown |
| M658V | Missense Mutation | Hepatocellular Carcinoma | Unknown |
| A675G | Missense Mutation | Hepatocellular Carcinoma | Unknown |
| A314T | Missense Mutation | Pancreatic Adenocarcinoma | Unknown |
| S646G | Missense Mutation | Pancreatic Adenocarcinoma | Unknown |
| T236S | Missense Mutation | Papillary Renal Cell Carcinoma | Unknown |
| N113K | Missense Mutation | Cutaneous Melanoma | Unknown |
| S1211F | Missense Mutation | Cutaneous Melanoma | Unknown |
| Q1299L | Missense Mutation | Cutaneous Melanoma | Unknown |
| L269V | Missense Mutation | Cutaneous Melanoma | Unknown |
| P811S | Missense Mutation | Cutaneous Melanoma | Unknown |
| P747L | Missense Mutation | Melanoma | Unknown |
| G1471R | Missense Mutation | Hepatocellular Carcinoma | Unknown |
| C1768Y | Missense Mutation | Hepatocellular Carcinoma | Unknown |
| V1590A | Missense Mutation | Pancreatic Adenocarcinoma | Unknown |
| R331G | Missense Mutation | Hepatocellular Carcinoma | Unknown |
| L6P | Missense Mutation | Hepatocellular Carcinoma | Unknown |
| S157C | Missense Mutation | Lung Adenocarcinoma | Unknown |
| S956C | Missense Mutation | Uveal Melanoma | Unknown |
| L1570P | Missense Mutation | Cutaneous Melanoma | Unknown |
| N306K | Missense Mutation | Renal Clear Cell Carcinoma | Unknown |
| T77S | Missense Mutation | Lung Adenocarcinoma | Unknown |
| E1440Q | Missense Mutation | Lung Squamous Cell Carcinoma | Unknown |
| L1800V | Missense Mutation | Lung Squamous Cell Carcinoma | Unknown |
| T1677Ifs*2 | Frame Shift Deletion | Serous Ovarian Cancer | Likely Oncogenic |
| C24Sfs*16 | Frame Shift Deletion | Serous Ovarian Cancer | Likely Oncogenic |

| **BRCA2 Mutation** | **Mutation Type** | **Cancer Type** | **OncoKB Analysis** |
| --- | --- | --- | --- |
| T1354M | Missense Mutation | Serous Ovarian Cancer | Inconclusive |
| R2842C | Missense Mutation | Uterine Endometrioid Carcinoma | Likely Oncogenic |
| R2842C | Missense Mutation | Uterine Endometrioid Carcinoma | Likely Oncogenic |
| K1638E | Missense Mutation | Serous Ovarian Cancer | Unknown |
| I1017F | Missense Mutation | Pancreatic Adenocarcinoma | Unknown |
| T3033Nfs*11 | Frame Shift Insertion | Stomach Adenocarcinoma | Likely Oncogenic |
| T3033Lfs*29 | Frame Shift Deletion | Diffuse Type Stomach Adenocarcinoma | Likely Oncogenic |
| E3342K | Missense Mutation | Tubular Stomach Adenocarcinoma | Unknown |
| E3342K | Missense Mutation | Breast Invasive Ductal Carcinoma | Unknown |
| E3342K | Missense Mutation | Head and Neck Squamous Cell Carcinoma | Unknown |
| G1338D | Missense Mutation | Mucinous Stomach Adenocarcinoma | Unknown |
| S2243C | Missense Mutation | Lung Adenocarcinoma | Unknown |
| S2243Y | Missense Mutation | Rectal Adenocarcinoma | Unknown |
| R2336C | Missense Mutation | Thymoma | Likely Oncogenic |
| R2336C | Missense Mutation | Uterine Endometrioid Carcinoma | Likely Oncogenic |
| R2336C | Missense Mutation | Breast Invasive Ductal Carcinoma | Likely Oncogenic |
| N1784Kfs*3 | Frame Shift Insertion | Stomach Adenocarcinoma | Likely Oncogenic |
| N1784Kfs*3 | Frame Shift Insertion | Tubular Stomach Adenocarcinoma | Likely Oncogenic |
| N1784Kfs*3 | Frame Shift Insertion | Stomach Adenocarcinoma | Likely Oncogenic |
| N1784Kfs*3 | Frame Shift Insertion | Esophageal Squamous Cell Carcinoma | Likely Oncogenic |
| I605Yfs*9 | Frame Shift Deletion | Stomach Adenocarcinoma | Likely Oncogenic |
| I605Yfs*9 | Frame Shift Deletion | Stomach Adenocarcinoma | Likely Oncogenic |
| I605Yfs*9 | Frame Shift Deletion | Head and Neck Squamous Cell Carcinoma | Likely Oncogenic |
| E510* | Nonsense Mutation | Uterine Endometrioid Carcinoma | Likely Oncogenic |
| S1064* | Nonsense Mutation | Uterine Endometrioid Carcinoma | Likely Oncogenic |
| R3052Q | Missense Mutation | Uterine Endometrioid Carcinoma | Likely Oncogenic |
| Q2870* | Nonsense Mutation | Cutaneous Melanoma | Likely Oncogenic |
| Q2870* | Nonsense Mutation | Bladder Urothelial Carcinoma | Likely Oncogenic |
| E187* | Nonsense Mutation | Lung Squamous Cell Carcinoma | Likely Oncogenic |
| R3052W | Missense Mutation | Colon Adenocarcinoma | Likely Oncogenic |
| S1882* | Nonsense Mutation | Serous Ovarian Cancer | Likely Oncogenic |
| T1887M | Missense Mutation | Uterine Endometrioid Carcinoma | Unknown |
| S3332Y | Missense Mutation | Uterine Endometrioid Carcinoma | Unknown |
| N1435T | Missense Mutation | Prostate Adenocarcinoma | Unknown |
| D281H | Missense Mutation | Prostate Adenocarcinoma | Unknown |
| P288S | Missense Mutation | Uterine Endometrioid Carcinoma | Unknown |
| S1172L | Missense Mutation | Uterine Endometrioid Carcinoma | Likely Neutral |
| I332F | Missense Mutation | Stomach Adenocarcinoma | Unknown |
| C1159S | Missense Mutation | Stomach Adenocarcinoma | Unknown |
| V348A | Missense Mutation | Stomach Adenocarcinoma | Unknown |
| F1192C | Missense Mutation | Uterine Endometrioid Carcinoma | Unknown |
| F1192C | Missense Mutation | Colon Adenocarcinoma | Unknown |
| P606L | Missense Mutation | Cutaneous Melanoma | Unknown |
| P606L | Missense Mutation | Head and Neck Squamous Cell Carcinoma | Unknown |
| R1512H | Missense Mutation | Lung Adenocarcinoma | Unknown |
| R1512H | Missense Mutation | Lung Adenocarcinoma | Unknown |
| K2316Q | Missense Mutation | Uterine Endometrioid Carcinoma | Unknown |
| E2020K | Missense Mutation | Uterine Endometrioid Carcinoma | Unknown |
| E2020K | Missense Mutation | Uterine Endometrioid Carcinoma | Unknown |
| C1159Y | Missense Mutation | Uterine Endometrioid Carcinoma | Unknown |
| R2888H | Missense Mutation | Uterine Endometrioid Carcinoma | Unknown |
| R155I | Missense Mutation | Uterine Endometrioid Carcinoma | Unknown |
| R2494Q | Missense Mutation | Uterine Endometrioid Carcinoma | Likely Neutral |
| R1512C | Missense Mutation | Uterine Endometrioid Carcinoma | Unknown |
| D1420N | Missense Mutation | Uterine Endometrioid Carcinoma | Unknown |
| S1597Y | Missense Mutation | Uterine Endometrioid Carcinoma | Unknown |
| R2268I | Missense Mutation | Uterine Endometrioid Carcinoma | Unknown |
| R2418I | Missense Mutation | Uterine Endometrioid Carcinoma | Unknown |
| E2258K | Missense Mutation | Bladder Urothelial Carcinoma | Unknown |
| E832K | Missense Mutation | Renal Clear Cell Carcinoma | Unknown |
| K2316R | Missense Mutation | Lung Squamous Cell Carcinoma | Unknown |
| K2316R | Missense Mutation | Lung Squamous Cell Carcinoma | Unknown |
| E764K | Missense Mutation | Lung Adenocarcinoma | Unknown |
| E832G | Missense Mutation | Colon Adenocarcinoma | Unknown |
| I332M | Missense Mutation | Mucinous Adenocarcinoma of the Colon and Rectum | Unknown |
| D479Y | Missense Mutation | Colon Adenocarcinoma | Unknown |
| P606S | Missense Mutation | Cutaneous Melanoma | Unknown |
| K3416Nfs*11 | Frame Shift Deletion | Stomach Adenocarcinoma | Likely Oncogenic |
| L2926* | Nonsense Mutation | Breast Invasive Ductal Carcinoma | Likely Oncogenic |
| Y2884* | Nonsense Mutation | Head and Neck Squamous Cell Carcinoma | Likely Oncogenic |
| N986Ifs*5 | Frame Shift Deletion | Stomach Adenocarcinoma | Likely Oncogenic |
| T2515Hfs*9 | Frame Shift Deletion | Stomach Adenocarcinoma | Likely Oncogenic |
| S3376* | Nonsense Mutation | Lung Adenocarcinoma | Likely Oncogenic |
| S2695* | Nonsense Mutation | Stomach Adenocarcinoma | Likely Oncogenic |
| S2695* | Nonsense Mutation | Lung Adenocarcinoma | Likely Oncogenic |
| S2695* | Nonsense Mutation | Lung Adenocarcinoma | Likely Oncogenic |
| K604* | Nonsense Mutation | Uterine Endometrioid Carcinoma | Likely Oncogenic |
| S196I | Missense Mutation | Uterine Endometrioid Carcinoma | Likely Oncogenic |
| E897* | Nonsense Mutation | Uterine Endometrioid Carcinoma | Likely Oncogenic |
| E2476* | Nonsense Mutation | Uterine Endometrioid Carcinoma | Likely Oncogenic |
| E1441* | Nonsense Mutation | Uterine Endometrioid Carcinoma | Likely Oncogenic |
| Q2157* | Nonsense Mutation | Bladder Urothelial Carcinoma | Likely Oncogenic |
| S1341* | Nonsense Mutation | Bladder Urothelial Carcinoma | Likely Oncogenic |
| Q499* | Nonsense Mutation | Lung Squamous Cell Carcinoma | Likely Oncogenic |
| Q499* | Nonsense Mutation | Lung Squamous Cell Carcinoma | Likely Oncogenic |
| Y2660C | Missense Mutation | Lung Squamous Cell Carcinoma | Likely Oncogenic |
| S1650Vfs*20 | Frame Shift Deletion | Colon Adenocarcinoma | Likely Oncogenic |
| Y2997* | Nonsense Mutation | Colon Adenocarcinoma | Likely Oncogenic |
| E49* | Nonsense Mutation | Rectal Adenocarcinoma | Likely Oncogenic |
| C711* | Nonsense Mutation | Serous Ovarian Cancer | Likely Oncogenic |
| K1406Nfs*3 | Frame Shift Deletion | Serous Ovarian Cancer | Likely Oncogenic |
| S1230Lfs*9 | Frame Shift Deletion | Serous Ovarian Cancer | Likely Oncogenic |
| S2697Kfs*31 | Frame Shift Deletion | Serous Ovarian Cancer | Likely Oncogenic |
| P3278Lfs*35 | Frame Shift Deletion | Serous Ovarian Cancer | Likely Oncogenic |
| P2608Qfs*40 | Frame Shift Deletion | Serous Ovarian Cancer | Likely Oncogenic |
| Q3026* | Nonsense Mutation | Cutaneous Melanoma | Likely Oncogenic |
| D1355Y | Missense Mutation | Breast Invasive Ductal Carcinoma | Unknown |
| V1270del | In Frame Deletion | Breast Invasive Ductal Carcinoma | Unknown |
| E2650Q | Missense Mutation | Breast Invasive Ductal Carcinoma | Unknown |
| E3177Q | Missense Mutation | Breast Invasive Ductal Carcinoma | Unknown |
| E2175Q | Missense Mutation | Breast Invasive Lobular Carcinoma | Unknown |
| E2175Q | Missense Mutation | Head and Neck Squamous Cell Carcinoma | Unknown |
| E2175Q | Missense Mutation | Lung Squamous Cell Carcinoma | Unknown |
| H1752N | Missense Mutation | Glioblastoma Multiforme | Unknown |
| H1966R | Missense Mutation | Glioblastoma Multiforme | Unknown |
| L1091F | Missense Mutation | Oligoastrocytoma | Unknown |
| A1393V | Missense Mutation | Tubular Stomach Adenocarcinoma | Unknown |
| K2741N | Missense Mutation | Uterine Endometrioid Carcinoma | Unknown |
| D1911N | Missense Mutation | Bladder Urothelial Carcinoma | Unknown |
| A2351T | Missense Mutation | Stomach Adenocarcinoma | Unknown |
| A1439T | Missense Mutation | Stomach Adenocarcinoma | Unknown |
| A2951V | Missense Mutation | Stomach Adenocarcinoma | Unknown |
| N1619D | Missense Mutation | Stomach Adenocarcinoma | Unknown |
| V2545I | Missense Mutation | Stomach Adenocarcinoma | Unknown |
| Q3262P | Missense Mutation | Stomach Adenocarcinoma | Unknown |
| K3313Q | Missense Mutation | Stomach Adenocarcinoma | Unknown |
| S1121T | Missense Mutation | Stomach Adenocarcinoma | Unknown |
| R468I | Missense Mutation | Tubular Stomach Adenocarcinoma | Unknown |
| N2452T | Missense Mutation | Tubular Stomach Adenocarcinoma | Unknown |
| P2734H | Missense Mutation | Stomach Adenocarcinoma | Unknown |
| P2352S | Missense Mutation | Stomach Adenocarcinoma | Unknown |
| V1810I | Missense Mutation | Stomach Adenocarcinoma | Unknown |
| S2022L | Missense Mutation | Stomach Adenocarcinoma | Unknown |
| I3418S | Missense Mutation | Stomach Adenocarcinoma | Unknown |
| N852K | Missense Mutation | Tubular Stomach Adenocarcinoma | Unknown |
| V494A | Missense Mutation | Diffuse Type Stomach Adenocarcinoma | Unknown |
| S3375G | Missense Mutation | Tubular Stomach Adenocarcinoma | Unknown |
| Q2539H | Missense Mutation | Diffuse Type Stomach Adenocarcinoma | Unknown |
| T874I | Missense Mutation | Lung Adenocarcinoma | Unknown |
| D1425H | Missense Mutation | Lung Adenocarcinoma | Unknown |
| V741L | Missense Mutation | Lung Adenocarcinoma | Unknown |
| I2718V | Missense Mutation | Lung Adenocarcinoma | Unknown |
| I2718V | Missense Mutation | Lung Adenocarcinoma | Unknown |
| P2608S | Missense Mutation | Lung Adenocarcinoma | Unknown |
| P2608S | Missense Mutation | Lung Adenocarcinoma | Unknown |
| E3403Q | Missense Mutation | Lung Adenocarcinoma | Unknown |
| C554F | Missense Mutation | Lung Adenocarcinoma | Unknown |
| C554F | Missense Mutation | Lung Adenocarcinoma | Unknown |
| E1646Q | Missense Mutation | Lung Adenocarcinoma | Unknown |
| H3117N | Missense Mutation | Lung Adenocarcinoma | Unknown |
| H3117N | Missense Mutation | Lung Adenocarcinoma | Unknown |
| I2149V | Missense Mutation | Lung Adenocarcinoma | Unknown |
| I2149V | Missense Mutation | Lung Adenocarcinoma | Unknown |
| A577S | Missense Mutation | Lung Adenocarcinoma | Unknown |
| A577S | Missense Mutation | Lung Adenocarcinoma | Unknown |
| T1067A | Missense Mutation | Uterine Endometrioid Carcinoma | Unknown |
| V1078I | Missense Mutation | Uterine Endometrioid Carcinoma | Unknown |
| T2880I | Missense Mutation | Uterine Endometrioid Carcinoma | Unknown |
| T868I | Missense Mutation | Uterine Endometrioid Carcinoma | Unknown |
| I1772S | Missense Mutation | Uterine Endometrioid Carcinoma | Unknown |
| S3239Y | Missense Mutation | Uterine Endometrioid Carcinoma | Unknown |
| P143S | Missense Mutation | Uterine Endometrioid Carcinoma | Unknown |
| A1725S | Missense Mutation | Uterine Endometrioid Carcinoma | Unknown |
| G1761V | Missense Mutation | Uterine Endometrioid Carcinoma | Unknown |
| A1981S | Missense Mutation | Uterine Endometrioid Carcinoma | Unknown |
| K2206N | Missense Mutation | Uterine Endometrioid Carcinoma | Unknown |
| S2216F | Missense Mutation | Uterine Endometrioid Carcinoma | Unknown |
| L759I | Missense Mutation | Uterine Endometrioid Carcinoma | Unknown |
| G2584C | Missense Mutation | Uterine Endometrioid Carcinoma | Unknown |
| S1680Y | Missense Mutation | Uterine Endometrioid Carcinoma | Unknown |
| S3396R | Missense Mutation | Uterine Endometrioid Carcinoma | Unknown |
| T2542M | Missense Mutation | Uterine Endometrioid Carcinoma | Unknown |
| L482I | Missense Mutation | Uterine Endometrioid Carcinoma | Unknown |
| Y1569C | Missense Mutation | Uterine Endometrioid Carcinoma | Unknown |
| N1747S | Missense Mutation | Uterine Endometrioid Carcinoma | Unknown |
| A2711T | Missense Mutation | Uterine Endometrioid Carcinoma | Unknown |
| Q3247H | Missense Mutation | Uterine Endometrioid Carcinoma | Unknown |
| D988N | Missense Mutation | Uterine Endometrioid Carcinoma | Unknown |
| R2488T | Missense Mutation | Uterine Endometrioid Carcinoma | Unknown |
| A843V | Missense Mutation | Uterine Endometrioid Carcinoma | Unknown |
| K1139N | Missense Mutation | Uterine Endometrioid Carcinoma | Unknown |
| I1607S | Missense Mutation | Uterine Endometrioid Carcinoma | Unknown |
| C2473R | Missense Mutation | Uterine Endometrioid Carcinoma | Unknown |
| H1350N | Missense Mutation | Uterine Endometrioid Carcinoma | Unknown |
| E1382D | Missense Mutation | Uterine Endometrioid Carcinoma | Unknown |
| T1785I | Missense Mutation | Uterine Endometrioid Carcinoma | Unknown |
| T2097M | Missense Mutation | Bladder Urothelial Carcinoma | Unknown |
| E2081Q | Missense Mutation | Bladder Urothelial Carcinoma | Unknown |
| E732Q | Missense Mutation | Bladder Urothelial Carcinoma | Unknown |
| S708Y | Missense Mutation | Bladder Urothelial Carcinoma | Unknown |
| M192T | Missense Mutation | Bladder Urothelial Carcinoma | Unknown |
| E1126Q | Missense Mutation | Bladder Urothelial Carcinoma | Unknown |
| D1386Y | Missense Mutation | Bladder Urothelial Carcinoma | Unknown |
| R1570T | Missense Mutation | Bladder Urothelial Carcinoma | Unknown |
| E1895K | Missense Mutation | Bladder Urothelial Carcinoma | Unknown |
| I2177V | Missense Mutation | Renal Clear Cell Carcinoma | Unknown |
| D125G | Missense Mutation | Renal Clear Cell Carcinoma | Unknown |
| D189H | Missense Mutation | Renal Clear Cell Carcinoma | Unknown |
| S313Y | Missense Mutation | Renal Clear Cell Carcinoma | Unknown |
| F2841I | Missense Mutation | Renal Clear Cell Carcinoma | Unknown |
| A2698T | Missense Mutation | Glioblastoma Multiforme | Unknown |
| S2201F | Missense Mutation | Lung Squamous Cell Carcinoma | Unknown |
| S2201F | Missense Mutation | Lung Squamous Cell Carcinoma | Unknown |
| V2820I | Missense Mutation | Lung Squamous Cell Carcinoma | Unknown |
| V2820I | Missense Mutation | Lung Squamous Cell Carcinoma | Unknown |
| S2098T | Missense Mutation | Lung Squamous Cell Carcinoma | Unknown |
| S2098T | Missense Mutation | Lung Squamous Cell Carcinoma | Unknown |
| I2822T | Missense Mutation | Lung Squamous Cell Carcinoma | Unknown |
| I2822T | Missense Mutation | Lung Squamous Cell Carcinoma | Unknown |
| E1036K | Missense Mutation | Lung Squamous Cell Carcinoma | Unknown |
| E1036K | Missense Mutation | Lung Squamous Cell Carcinoma | Unknown |
| R2034H | Missense Mutation | Lung Squamous Cell Carcinoma | Unknown |
| R2034H | Missense Mutation | Lung Squamous Cell Carcinoma | Unknown |
| R2034H | Missense Mutation | Lung Squamous Cell Carcinoma | Unknown |
| W993C | Missense Mutation | Lung Squamous Cell Carcinoma | Unknown |
| W993C | Missense Mutation | Lung Squamous Cell Carcinoma | Unknown |
| G2063R | Missense Mutation | Lung Adenocarcinoma | Unknown |
| V2503F | Missense Mutation | Lung Adenocarcinoma | Unknown |
| V2503L | Missense Mutation | Lung Squamous Cell Carcinoma | Unknown |
| K1541E | Missense Mutation | Lung Adenocarcinoma | Unknown |
| L2917F | Missense Mutation | Cutaneous Melanoma | Unknown |
| P2107S | Missense Mutation | Cutaneous Melanoma | Unknown |
| H334Y | Missense Mutation | Cutaneous Melanoma | Unknown |
| H334Y | Missense Mutation | Cutaneous Melanoma | Unknown |
| S1792F | Missense Mutation | Cutaneous Melanoma | Unknown |
| Y3049S | Missense Mutation | Breast Invasive Ductal Carcinoma | Unknown |
| D687H | Missense Mutation | Breast Invasive Ductal Carcinoma | Unknown |
| D1033H | Missense Mutation | Breast Invasive Ductal Carcinoma | Unknown |
| E1158Q | Missense Mutation | Invasive Breast Carcinoma | Unknown |
| K1191N | Missense Mutation | Invasive Breast Carcinoma | Unknown |
| H2932Y | Missense Mutation | Papillary Thyroid Cancer | Unknown |
| A2738S | Missense Mutation | Papillary Thyroid Cancer | Unknown |
| C1820R | Missense Mutation | Papillary Thyroid Cancer | Unknown |
| C3304S | Missense Mutation | Breast Invasive Ductal Carcinoma | Unknown |
| F2568C | Missense Mutation | Colon Adenocarcinoma | Unknown |
| F2158C | Missense Mutation | Mucinous Adenocarcinoma of the Colon and Rectum | Unknown |
| T3085A | Missense Mutation | Mucinous Adenocarcinoma of the Colon and Rectum | Unknown |
| S3319Y | Missense Mutation | Colon Adenocarcinoma | Unknown |
| L2155H | Missense Mutation | Rectal Adenocarcinoma | Unknown |
| Q321H | Missense Mutation | Rectal Adenocarcinoma | Unknown |
| R3384Q | Missense Mutation | Rectal Adenocarcinoma | Unknown |
| E456D | Missense Mutation | Rectal Adenocarcinoma | Unknown |
| E1879D | Missense Mutation | Rectal Adenocarcinoma | Unknown |
| D2218Y | Missense Mutation | Rectal Adenocarcinoma | Unknown |
| K2017T | Missense Mutation | Rectal Adenocarcinoma | Unknown |
| S3016Y | Missense Mutation | Rectal Adenocarcinoma | Unknown |
| F2349C | Missense Mutation | Rectal Adenocarcinoma | Unknown |
| D1386N | Missense Mutation | Head and Neck Squamous Cell Carcinoma | Unknown |
| T1346N | Missense Mutation | Pancreatic Adenocarcinoma | Unknown |
| T3401M | Missense Mutation | Pheochromocytoma | Unknown |
| K3313T | Missense Mutation | Papillary Renal Cell Carcinoma | Unknown |
| I580L | Missense Mutation | Papillary Renal Cell Carcinoma | Unknown |
| K1264E | Missense Mutation | Cutaneous Melanoma | Unknown |
| L1598F | Missense Mutation | Cutaneous Melanoma | Unknown |
| L35F | Missense Mutation | Cutaneous Melanoma | Unknown |
| Q3398H | Missense Mutation | Cutaneous Melanoma | Unknown |
| S3252F | Missense Mutation | Cutaneous Melanoma | Unknown |
| H223Y | Missense Mutation | Cutaneous Melanoma | Unknown |
| E626K | Missense Mutation | Cutaneous Melanoma | Unknown |
| P3324L | Missense Mutation | Cutaneous Melanoma | Unknown |
| H1752Y | Missense Mutation | Cutaneous Melanoma | Unknown |
| R316K | Missense Mutation | Cutaneous Melanoma | Unknown |
| S2307L | Missense Mutation | Cutaneous Melanoma | Unknown |
| P555L | Missense Mutation | Cutaneous Melanoma | Unknown |
| S2704F | Missense Mutation | Cutaneous Melanoma | Unknown |
| L3180F | Missense Mutation | Uterine Carcinosarcoma/Uterine Malignant Mixed Mullerian Tumor | Unknown |
| Q3398R | Missense Mutation | Uterine Carcinosarcoma/Uterine Malignant Mixed Mullerian Tumor | Unknown |
| S879F | Missense Mutation | Breast Invasive Ductal Carcinoma | Unknown |
| D224V | Missense Mutation | Glioblastoma Multiforme | Unknown |
| S2067F | Missense Mutation | Cutaneous Melanoma | Unknown |
| Q1987E | Missense Mutation | Cutaneous Melanoma | Unknown |
| X2602_splice | Splice Site | Breast Invasive Ductal Carcinoma | Likely Oncogenic |
| T3085Nfs*26 | Frame Shift Insertion | Stomach Adenocarcinoma | Likely Oncogenic |
| N433Tfs*27 | Frame Shift Deletion | Prostate Adenocarcinoma | Likely Oncogenic |
| S3364Ifs*4 | Frame Shift Insertion | Prostate Adenocarcinoma | Likely Oncogenic |
| C916Vfs*2 | Frame Shift Deletion | Prostate Adenocarcinoma | Likely Oncogenic |
| E2981Rfs*37 | Frame Shift Insertion | Stomach Adenocarcinoma | Likely Oncogenic |
| L759Ffs*3 | Frame Shift Deletion | Stomach Adenocarcinoma | Likely Oncogenic |
| P1088Lfs*16 | Frame Shift Deletion | Mucinous Stomach Adenocarcinoma | Likely Oncogenic |
| D946Ifs*14 | Frame Shift Deletion | Diffuse Type Stomach Adenocarcinoma | Likely Oncogenic |
| R2625* | Nonsense Mutation | Head and Neck Squamous Cell Carcinoma | Likely Oncogenic |
| X211_splice | Splice Site | Head and Neck Squamous Cell Carcinoma | Likely Oncogenic |
| Q2655* | Nonsense Mutation | Bladder Urothelial Carcinoma | Likely Oncogenic |
| X2659_splice | Splice Site | Lung Squamous Cell Carcinoma | Likely Oncogenic |
| X2659_splice | Splice Site | Lung Squamous Cell Carcinoma | Likely Oncogenic |
| E294* | Nonsense Mutation | Esophageal Squamous Cell Carcinoma | Likely Oncogenic |
| R2500Sfs*24 | Frame Shift Deletion | Lung Squamous Cell Carcinoma | Likely Oncogenic |
| S1099* | Nonsense Mutation | Lung Squamous Cell Carcinoma | Likely Oncogenic |
| L1390Ffs*13 | Frame Shift Insertion | Lung Squamous Cell Carcinoma | Likely Oncogenic |
| K3015* | Nonsense Mutation | Lung Adenocarcinoma | Likely Oncogenic |
| X159_splice | Splice Site | Lung Squamous Cell Carcinoma | Likely Oncogenic |
| E1120* | Nonsense Mutation | Lung Adenocarcinoma | Likely Oncogenic |
| L1965Ffs*39 | Frame Shift Deletion | Breast Invasive Ductal Carcinoma | Likely Oncogenic |
| T3085Qfs*19 | Frame Shift Deletion | Stomach Adenocarcinoma | Likely Oncogenic |
| V2716Wfs*17 | Frame Shift Deletion | Pancreatic Adenocarcinoma | Likely Oncogenic |
| S206C | Missense Mutation | Esophageal Adenocarcinoma | Likely Oncogenic |
| V726Sfs*25 | Frame Shift Insertion | Prostate Adenocarcinoma | Likely Oncogenic |
| N213Mfs*17 | Frame Shift Deletion | Prostate Adenocarcinoma | Likely Oncogenic |
| N1344Mfs*30 | Frame Shift Deletion | Mucinous Stomach Adenocarcinoma | Likely Oncogenic |
| D946Rfs*13 | Frame Shift Insertion | Diffuse Type Stomach Adenocarcinoma | Likely Oncogenic |
| T219Lfs*11 | Frame Shift Deletion | Leiomyosarcoma | Likely Oncogenic |
| T2367Lfs*9 | Frame Shift Deletion | Uterine Carcinosarcoma/Uterine Malignant Mixed Mullerian Tumor | Likely Oncogenic |
| F2841Kfs*24 | Frame Shift Deletion | Uveal Melanoma | Likely Oncogenic |
| Q2749* | Nonsense Mutation | Head and Neck Squamous Cell Carcinoma | Likely Oncogenic |
| Y839Ifs*42 | Frame Shift Insertion | Head and Neck Squamous Cell Carcinoma | Likely Oncogenic |
| Y2726C | Missense Mutation | Hepatocellular Carcinoma | Likely Oncogenic |
| N863Kfs*18 | Frame Shift Insertion | Stomach Adenocarcinoma | Likely Oncogenic |
| E1407Vfs*5 | Frame Shift Deletion | Serous Ovarian Cancer | Likely Oncogenic |
| G500V | Missense Mutation | Head and Neck Squamous Cell Carcinoma | Unknown |
| V1605D | Missense Mutation | Head and Neck Squamous Cell Carcinoma | Unknown |
| E1571K | Missense Mutation | Head and Neck Squamous Cell Carcinoma | Unknown |
| I2105V | Missense Mutation | Head and Neck Squamous Cell Carcinoma | Unknown |
| E2903K | Missense Mutation | Head and Neck Squamous Cell Carcinoma | Unknown |
| K3315N | Missense Mutation | Head and Neck Squamous Cell Carcinoma | Unknown |
| L2654F | Missense Mutation | Bladder Urothelial Carcinoma | Unknown |
| L2753F | Missense Mutation | Bladder Urothelial Carcinoma | Unknown |
| P2800R | Missense Mutation | Renal Clear Cell Carcinoma | Likely Neutral |
| S2695L | Missense Mutation | Hepatocellular Carcinoma | Unknown |
| S2695L | Missense Mutation | Cutaneous Melanoma | Unknown |
| G1771C | Missense Mutation | Lung Adenocarcinoma | Unknown |
| R212I | Missense Mutation | Lung Squamous Cell Carcinoma | Unknown |
| K1274N | Missense Mutation | Lung Squamous Cell Carcinoma | Unknown |
| P1133L | Missense Mutation | Lung Squamous Cell Carcinoma | Unknown |
| K2673E | Missense Mutation | Lung Adenocarcinoma | Unknown |
| K2673E | Missense Mutation | Lung Adenocarcinoma | Unknown |
| N978S | Missense Mutation | Lung Adenocarcinoma | Unknown |
| E2391K | Missense Mutation | Lung Adenocarcinoma | Unknown |
| V290I | Missense Mutation | Lung Adenocarcinoma | Unknown |
| A186S | Missense Mutation | Lung Squamous Cell Carcinoma | Unknown |
| L2972W | Missense Mutation | Lung Squamous Cell Carcinoma | Inconclusive |
| S44C | Missense Mutation | Lung Adenocarcinoma | Unknown |
| S1685L | Missense Mutation | Lung Adenocarcinoma | Unknown |
| S396C | Missense Mutation | Lung Squamous Cell Carcinoma | Unknown |
| M2235L | Missense Mutation | Lung Squamous Cell Carcinoma | Unknown |
| V1306F | Missense Mutation | Lung Adenocarcinoma | Unknown |
| D1699G | Missense Mutation | Lung Squamous Cell Carcinoma | Unknown |
| N2101K | Missense Mutation | Lung Squamous Cell Carcinoma | Unknown |
| M2262T | Missense Mutation | Lung Adenocarcinoma | Unknown |
| P3067S | Missense Mutation | Lung Adenocarcinoma | Unknown |
| Y1716C | Missense Mutation | Lung Squamous Cell Carcinoma | Unknown |
| Y1749H | Missense Mutation | Lung Adenocarcinoma | Unknown |
| L833R | Missense Mutation | Lung Adenocarcinoma | Unknown |
| H1223Y | Missense Mutation | Lung Squamous Cell Carcinoma | Unknown |
| S497L | Missense Mutation | Lung Squamous Cell Carcinoma | Unknown |
| N1878K | Missense Mutation | Diffuse Large B-Cell Lymphoma | Inconclusive |
| D913E | Missense Mutation | Esophageal Adenocarcinoma | Unknown |
| P3054S | Missense Mutation | Esophageal Squamous Cell Carcinoma | Unknown |
| M124V | Missense Mutation | Esophageal Adenocarcinoma | Unknown |
| S1259R | Missense Mutation | Esophageal Adenocarcinoma | Unknown |
| S270L | Missense Mutation | Esophageal Adenocarcinoma | Unknown |
| P3063S | Missense Mutation | Esophageal Squamous Cell Carcinoma | Unknown |
| V2190I | Missense Mutation | Prostate Adenocarcinoma | Unknown |
| E2275D | Missense Mutation | Prostate Adenocarcinoma | Unknown |
| N1935D | Missense Mutation | Stomach Adenocarcinoma | Unknown |
| Q3047H | Missense Mutation | Stomach Adenocarcinoma | Unknown |
| V2118L | Missense Mutation | Stomach Adenocarcinoma | Unknown |
| V1804I | Missense Mutation | Leiomyosarcoma | Unknown |
| N1805D | Missense Mutation | Uterine Carcinosarcoma/Uterine Malignant Mixed Mullerian Tumor | Unknown |
| M3118V | Missense Mutation | Thymoma | Unknown |
| E1514Q | Missense Mutation | Intrahepatic Cholangiocarcinoma | Unknown |
| S3218F | Missense Mutation | Head and Neck Squamous Cell Carcinoma | Unknown |
| R2861T | Missense Mutation | Head and Neck Squamous Cell Carcinoma | Unknown |
| F312C | Missense Mutation | Head and Neck Squamous Cell Carcinoma | Unknown |
| S3231L | Missense Mutation | Head and Neck Squamous Cell Carcinoma | Unknown |
| E3393Q | Missense Mutation | Head and Neck Squamous Cell Carcinoma | Unknown |
| S3123G | Missense Mutation | Head and Neck Squamous Cell Carcinoma | Likely Neutral |
| Y2624N | Missense Mutation | Hepatocellular Carcinoma | Unknown |
| D2995_Y2997del | In Frame Deletion | Hepatocellular Carcinoma | Unknown |
| H2074D | Missense Mutation | Hepatocellular Carcinoma | Unknown |
| A1996G | Missense Mutation | Hepatocellular Carcinoma | Unknown |
| H1332Y | Missense Mutation | Hepatocellular Carcinoma | Unknown |
| S278N | Missense Mutation | Pancreatic Adenocarcinoma | Unknown |
| N1642T | Missense Mutation | Pancreatic Adenocarcinoma | Unknown |
| V2079M | Missense Mutation | Pancreatic Adenocarcinoma | Unknown |
| C3097R | Missense Mutation | Pheochromocytoma | Unknown |
| N556S | Missense Mutation | Papillary Renal Cell Carcinoma | Unknown |
| E816G | Missense Mutation | Papillary Renal Cell Carcinoma | Unknown |
| G2529D | Missense Mutation | Cutaneous Melanoma | Unknown |
| G2529S | Missense Mutation | Cutaneous Melanoma | Unknown |
| M1090L | Missense Mutation | Uterine Carcinosarcoma/Uterine Malignant Mixed Mullerian Tumor | Unknown |
| N517S | Missense Mutation | Oligodendroglioma | Inconclusive |
| P740S | Missense Mutation | Cutaneous Melanoma | Unknown |
| S1115F | Missense Mutation | Stomach Adenocarcinoma | Unknown |
| F3298C | Missense Mutation | Hepatocellular Carcinoma | Unknown |
| E1245G | Missense Mutation | Hepatocellular Carcinoma | Unknown |
| K2111E | Missense Mutation | Hepatocellular Carcinoma | Unknown |
| S2887L | Missense Mutation | Hepatocellular Carcinoma | Unknown |
| F892L | Missense Mutation | Hepatocellular Carcinoma | Unknown |
| G2313V | Missense Mutation | Non-Seminomatous Germ Cell Tumor | Unknown |
| L2838F | Missense Mutation | Uveal Melanoma | Unknown |
| P2827L | Missense Mutation | Lentigo Maligna Melanoma | Unknown |
| V733_L734InsFFF | In Frame Insertion | Adrenocortical Carcinoma | Unknown |
| V3091I | Missense Mutation | Diffuse Large B-Cell Lymphoma | Unknown |
| Q3299R | Missense Mutation | Head and Neck Squamous Cell Carcinoma | Unknown |
| G1963W | Missense Mutation | Perihilar Cholangiocarcinoma | Unknown |
| V1663I | Missense Mutation | Intrahepatic Cholangiocarcinoma | Unknown |
| S131T | Missense Mutation | Lung Adenocarcinoma | Unknown |
| T2399A | Missense Mutation | Lung Squamous Cell Carcinoma | Unknown |
| F1372Y | Missense Mutation | Lung Squamous Cell Carcinoma | Unknown |
| R2394* | Nonsense Mutation | Serous Ovarian Cancer | Likely Oncogenic |
| K3326* | Nonsense Mutation | Serous Ovarian Cancer | Likely Neutral |

| **BRIP1 Mutation** | **Mutation Type** | **Cancer Type** | **OncoKB Analysis** |
| --- | --- | --- | --- |
| A745T | Missense Mutation | Uterine Endometrioid Carcinoma | Unknown |
| A745T | Missense Mutation | Head and Neck Squamous Cell Carcinoma | Unknown |
| A745V | Missense Mutation | Uterine Endometrioid Carcinoma | Unknown |
| M620V | Missense Mutation | Lung Adenocarcinoma | Unknown |
| M620V | Missense Mutation | Lung Adenocarcinoma | Unknown |
| A551V | Missense Mutation | Uterine Endometrioid Carcinoma | Unknown |
| A551V | Missense Mutation | Uterine Endometrioid Carcinoma | Unknown |
| M620I | Missense Mutation | Uterine Endometrioid Carcinoma | Unknown |
| S624L | Missense Mutation | Mucinous Adenocarcinoma of the Colon and Rectum | Unknown |
| R865W | Missense Mutation | Uterine Endometrioid Carcinoma | Likely Oncogenic |
| R865W | Missense Mutation | Tubular Stomach Adenocarcinoma | Likely Oncogenic |
| A453T | Missense Mutation | Uterine Endometrioid Carcinoma | Unknown |
| A453T | Missense Mutation | Breast Invasive Ductal Carcinoma | Unknown |
| L358P | Missense Mutation | Stomach Adenocarcinoma | Unknown |
| P1246L | Missense Mutation | Cutaneous Melanoma | Unknown |
| G1024R | Missense Mutation | Uterine Endometrioid Carcinoma | Unknown |
| P1246T | Missense Mutation | Uterine Endometrioid Carcinoma | Unknown |
| P1246T | Missense Mutation | Esophageal Adenocarcinoma | Unknown |
| E671Q | Missense Mutation | Bladder Urothelial Carcinoma | Unknown |
| E671Q | Missense Mutation | Lung Adenocarcinoma | Unknown |
| F934V | Missense Mutation | Breast Invasive Ductal Carcinoma | Unknown |
| G1024V | Missense Mutation | Diffuse Large B-Cell Lymphoma | Unknown |
| I504Sfs*22 | Frame Shift Deletion | Stomach Adenocarcinoma | Likely Oncogenic |
| Y1131Lfs*18 | Frame Shift Deletion | Lung Adenocarcinoma | Likely Oncogenic |
| E795* | Nonsense Mutation | Lung Adenocarcinoma | Likely Oncogenic |
| R251H | Missense Mutation | Uterine Endometrioid Carcinoma | Likely Oncogenic |
| E1097* | Nonsense Mutation | Uterine Endometrioid Carcinoma | Likely Oncogenic |
| S1117* | Nonsense Mutation | Colon Adenocarcinoma | Likely Oncogenic |
| Q685* | Nonsense Mutation | Cutaneous Melanoma | Likely Oncogenic |
| P47L | Missense Mutation | Cutaneous Melanoma | Likely Oncogenic |
| S913F | Missense Mutation | Breast Invasive Ductal Carcinoma | Unknown |
| L717V | Missense Mutation | Breast Invasive Lobular Carcinoma | Unknown |
| G290S | Missense Mutation | Mucinous Stomach Adenocarcinoma | Unknown |
| G290S | Missense Mutation | Esophageal Adenocarcinoma | Unknown |
| R798Q | Missense Mutation | Colon Adenocarcinoma | Unknown |
| R762H | Missense Mutation | Renal Clear Cell Carcinoma | Unknown |
| A267T | Missense Mutation | Lung Squamous Cell Carcinoma | Unknown |
| D898N | Missense Mutation | Tubular Stomach Adenocarcinoma | Unknown |
| V588L | Missense Mutation | Lung Adenocarcinoma | Unknown |
| E718V | Missense Mutation | Lung Adenocarcinoma | Unknown |
| A776S | Missense Mutation | Lung Adenocarcinoma | Unknown |
| T1193A | Missense Mutation | Lung Adenocarcinoma | Unknown |
| S1237F | Missense Mutation | Lung Adenocarcinoma | Unknown |
| S1237F | Missense Mutation | Lung Adenocarcinoma | Unknown |
| S38I | Missense Mutation | Lung Adenocarcinoma | Unknown |
| S38I | Missense Mutation | Lung Adenocarcinoma | Unknown |
| I1201V | Missense Mutation | Lung Adenocarcinoma | Unknown |
| I1201V | Missense Mutation | Lung Adenocarcinoma | Unknown |
| I1176L | Missense Mutation | Lung Adenocarcinoma | Unknown |
| I1176L | Missense Mutation | Lung Adenocarcinoma | Unknown |
| E404K | Missense Mutation | Lung Adenocarcinoma | Unknown |
| E404K | Missense Mutation | Lung Adenocarcinoma | Unknown |
| D1120A | Missense Mutation | Uterine Endometrioid Carcinoma | Unknown |
| F108S | Missense Mutation | Uterine Endometrioid Carcinoma | Unknown |
| K1086N | Missense Mutation | Uterine Endometrioid Carcinoma | Unknown |
| C88del | In Frame Deletion | Uterine Endometrioid Carcinoma | Unknown |
| S320T | Missense Mutation | Uterine Endometrioid Carcinoma | Unknown |
| I507M | Missense Mutation | Uterine Endometrioid Carcinoma | Unknown |
| S601P | Missense Mutation | Uterine Endometrioid Carcinoma | Unknown |
| V779I | Missense Mutation | Uterine Endometrioid Carcinoma | Unknown |
| K1205R | Missense Mutation | Uterine Endometrioid Carcinoma | Unknown |
| L84M | Missense Mutation | Bladder Urothelial Carcinoma | Unknown |
| L842V | Missense Mutation | Bladder Urothelial Carcinoma | Unknown |
| D363N | Missense Mutation | Bladder Urothelial Carcinoma | Unknown |
| K159E | Missense Mutation | Bladder Urothelial Carcinoma | Unknown |
| E910Q | Missense Mutation | Bladder Urothelial Carcinoma | Unknown |
| T238I | Missense Mutation | Renal Clear Cell Carcinoma | Unknown |
| D674Y | Missense Mutation | Renal Clear Cell Carcinoma | Unknown |
| G270R | Missense Mutation | Lung Squamous Cell Carcinoma | Unknown |
| G270R | Missense Mutation | Lung Squamous Cell Carcinoma | Unknown |
| P727Q | Missense Mutation | Lung Squamous Cell Carcinoma | Unknown |
| T722A | Missense Mutation | Lung Squamous Cell Carcinoma | Unknown |
| T722A | Missense Mutation | Lung Squamous Cell Carcinoma | Unknown |
| F694L | Missense Mutation | Lung Adenocarcinoma | Unknown |
| P1238S | Missense Mutation | Cutaneous Melanoma | Unknown |
| S206L | Missense Mutation | Cutaneous Melanoma | Unknown |
| E296D | Missense Mutation | Breast Invasive Lobular Carcinoma | Unknown |
| L134V | Missense Mutation | Breast Invasive Ductal Carcinoma | Unknown |
| E421Q | Missense Mutation | Breast Invasive Ductal Carcinoma | Unknown |
| S876N | Missense Mutation | Mucinous Adenocarcinoma of the Colon and Rectum | Unknown |
| S1129Y | Missense Mutation | Colon Adenocarcinoma | Unknown |
| Y353C | Missense Mutation | Colon Adenocarcinoma | Unknown |
| K950T | Missense Mutation | Rectal Adenocarcinoma | Unknown |
| T650I | Missense Mutation | Serous Ovarian Cancer | Unknown |
| S913A | Missense Mutation | Hepatocellular Carcinoma | Unknown |
| L679F | Missense Mutation | Cutaneous Melanoma | Unknown |
| G190E | Missense Mutation | Cutaneous Melanoma | Unknown |
| S956L | Missense Mutation | Cutaneous Melanoma | Unknown |
| S644L | Missense Mutation | Cutaneous Melanoma | Unknown |
| P1101L | Missense Mutation | Cutaneous Melanoma | Unknown |
| H317Y | Missense Mutation | Cutaneous Melanoma | Unknown |
| I983S | Missense Mutation | Cutaneous Melanoma | Unknown |
| G304E | Missense Mutation | Cutaneous Melanoma | Unknown |
| S895F | Missense Mutation | Cutaneous Melanoma | Unknown |
| X700_splice | Splice Site | Breast Invasive Ductal Carcinoma | Likely Oncogenic |
| M1? | Frame Shift Deletion | Mucinous Adenocarcinoma of the Colon and Rectum | Likely Oncogenic |
| X753_splice | Splice Site | Lung Adenocarcinoma | Likely Oncogenic |
| X753_splice | Splice Site | Lung Adenocarcinoma | Likely Oncogenic |
| N1087Kfs*4 | Frame Shift Insertion | Glioblastoma Multiforme | Likely Oncogenic |
| Q561* | Nonsense Mutation | Lung Adenocarcinoma | Likely Oncogenic |
| T1062Ifs*18 | Frame Shift Deletion | Lung Squamous Cell Carcinoma | Likely Oncogenic |
| Q126* | Nonsense Mutation | Esophageal Adenocarcinoma | Likely Oncogenic |
| R581* | Nonsense Mutation | Prostate Adenocarcinoma | Likely Oncogenic |
| A349V | Missense Mutation | Prostate Adenocarcinoma | Likely Oncogenic |
| V891Ffs*6 | Frame Shift Deletion | Mucinous Stomach Adenocarcinoma | Likely Oncogenic |
| S618* | Nonsense Mutation | Head and Neck Squamous Cell Carcinoma | Likely Oncogenic |
| X831_splice | Splice Site | Head and Neck Squamous Cell Carcinoma | Likely Oncogenic |
| K1040Dfs*4 | Frame Shift Deletion | Cutaneous Melanoma | Likely Oncogenic |
| X831_splice | Splice Site | Cutaneous Melanoma | Likely Oncogenic |
| X794_splice | Splice Site | Hepatocellular Carcinoma | Likely Oncogenic |
| A599= | Splice Region | Cutaneous Melanoma | Likely Oncogenic |
| G509F | Missense Mutation | Papillary Thyroid Cancer | Unknown |
| S1115F | Missense Mutation | Head and Neck Squamous Cell Carcinoma | Unknown |
| S123L | Missense Mutation | Head and Neck Squamous Cell Carcinoma | Unknown |
| L1158F | Missense Mutation | Prostate Adenocarcinoma | Unknown |
| T411A | Missense Mutation | Head and Neck Squamous Cell Carcinoma | Unknown |
| D280N | Missense Mutation | Head and Neck Squamous Cell Carcinoma | Unknown |
| G750V | Missense Mutation | Head and Neck Squamous Cell Carcinoma | Unknown |
| Q867L | Missense Mutation | Head and Neck Squamous Cell Carcinoma | Unknown |
| W863L | Missense Mutation | Head and Neck Squamous Cell Carcinoma | Unknown |
| I640M | Missense Mutation | Bladder Urothelial Carcinoma | Unknown |
| W708C | Missense Mutation | Lung Adenocarcinoma | Unknown |
| A89S | Missense Mutation | Lung Adenocarcinoma | Unknown |
| M331I | Missense Mutation | Lung Adenocarcinoma | Unknown |
| R378S | Missense Mutation | Lung Adenocarcinoma | Unknown |
| D1180H | Missense Mutation | Lung Adenocarcinoma | Unknown |
| E1220V | Missense Mutation | Lung Adenocarcinoma | Unknown |
| W1217G | Missense Mutation | Lung Adenocarcinoma | Unknown |
| G754V | Missense Mutation | Lung Squamous Cell Carcinoma | Unknown |
| R264W | Missense Mutation | Head and Neck Squamous Cell Carcinoma | Unknown |
| G12E | Missense Mutation | Esophageal Squamous Cell Carcinoma | Unknown |
| C332S | Missense Mutation | Esophageal Squamous Cell Carcinoma | Unknown |
| N1061S | Missense Mutation | Mucinous Stomach Adenocarcinoma | Unknown |
| K1078E | Missense Mutation | Leiomyosarcoma | Unknown |
| S309Y | Missense Mutation | Head and Neck Squamous Cell Carcinoma | Unknown |
| E150K | Missense Mutation | Head and Neck Squamous Cell Carcinoma | Unknown |
| S1063W | Missense Mutation | Head and Neck Squamous Cell Carcinoma | Unknown |
| N604S | Missense Mutation | Hepatocellular Carcinoma | Unknown |
| A1167V | Missense Mutation | Pancreatic Adenocarcinoma | Unknown |
| P272S | Missense Mutation | Pleural Mesothelioma, Biphasic Type | Unknown |
| A464G | Missense Mutation | Cutaneous Melanoma | Unknown |
| K1242T | Missense Mutation | Diffuse Large B-Cell Lymphoma | Unknown |
| V341D | Missense Mutation | Lung Adenocarcinoma | Unknown |
| M480I | Missense Mutation | Lung Adenocarcinoma | Unknown |
| D1148Y | Missense Mutation | Lung Squamous Cell Carcinoma | Unknown |

| **CDK12 Mutation** | **Mutation Type** | **Cancer Type** | **OncoKB Analysis** |
| --- | --- | --- | --- |
| S343Efs*8 | Frame Shift Insertion | Prostate Adenocarcinoma | Likely Oncogenic |
| Q598* | Nonsense Mutation | Prostate Adenocarcinoma | Likely Oncogenic |
| L1003Rfs*3 | Frame Shift Deletion | Prostate Adenocarcinoma | Likely Oncogenic |
| G909E | Missense Mutation | Prostate Adenocarcinoma | Likely Oncogenic |
| K217* | Nonsense Mutation | Prostate Adenocarcinoma | Likely Oncogenic |
| X349_splice | Splice Site | Prostate Adenocarcinoma | Likely Oncogenic |
| T588Ifs*48 | Frame Shift Deletion | Prostate Adenocarcinoma | Likely Oncogenic |
| Q696* | Nonsense Mutation | Prostate Adenocarcinoma | Likely Oncogenic |
| T592Dfs*49 | Frame Shift Insertion | Prostate Adenocarcinoma | Likely Oncogenic |
| R890H | Missense Mutation | Colon Adenocarcinoma | Predicted Oncogenic |
| T1463Nfs*50 | Frame Shift Insertion | Stomach Adenocarcinoma | Likely Oncogenic |
| T1463Nfs*50 | Frame Shift Insertion | Breast Invasive Ductal Carcinoma | Likely Oncogenic |
| T1463Nfs*50 | Frame Shift Insertion | Breast Invasive Ductal Carcinoma | Likely Oncogenic |
| T1463Nfs*50 | Frame Shift Insertion | Breast Invasive Ductal Carcinoma | Likely Oncogenic |
| T1463Nfs*50 | Frame Shift Insertion | Renal Clear Cell Carcinoma | Likely Oncogenic |
| T1463Nfs*50 | Frame Shift Insertion | Renal Clear Cell Carcinoma | Likely Oncogenic |
| T1463Nfs*50 | Frame Shift Insertion | Renal Clear Cell Carcinoma | Likely Oncogenic |
| E488* | Nonsense Mutation | Breast Invasive Ductal Carcinoma | Likely Oncogenic |
| S133Kfs*24 | Frame Shift Insertion | Glioblastoma Multiforme | Likely Oncogenic |
| Q1088* | Nonsense Mutation | Renal Clear Cell Carcinoma | Likely Oncogenic |
| G1461Afs*38 | Frame Shift Deletion | Stomach Adenocarcinoma | Likely Oncogenic |
| G1461Afs*38 | Frame Shift Deletion | Stomach Adenocarcinoma | Likely Oncogenic |
| G1461Afs*38 | Frame Shift Deletion | Head and Neck Squamous Cell Carcinoma | Likely Oncogenic |
| P604Lfs*6 | Frame Shift Deletion | Stomach Adenocarcinoma | Likely Oncogenic |
| R882Q | Missense Mutation | Stomach Adenocarcinoma | Likely Oncogenic |
| P683Qfs*70 | Frame Shift Deletion | Signet Ring Cell Carcinoma of the Stomach | Likely Oncogenic |
| G1446* | Nonsense Mutation | Tubular Stomach Adenocarcinoma | Likely Oncogenic |
| N868Ifs*8 | Frame Shift Deletion | Head and Neck Squamous Cell Carcinoma | Likely Oncogenic |
| K46* | Frame Shift Insertion | Lung Adenocarcinoma | Likely Oncogenic |
| R1067* | Nonsense Mutation | Uterine Endometrioid Carcinoma | Likely Oncogenic |
| L1210Gfs*23 | Frame Shift Deletion | Uterine Endometrioid Carcinoma | Likely Oncogenic |
| E95Rfs*29 | Frame Shift Deletion | Bladder Urothelial Carcinoma | Likely Oncogenic |
| I1188Tfs*27 | Frame Shift Deletion | Renal Clear Cell Carcinoma | Likely Oncogenic |
| N31Sfs*23 | Frame Shift Deletion | Lung Adenocarcinoma | Likely Oncogenic |
| L551Ffs*24 | Frame Shift Insertion | Lung Adenocarcinoma | Likely Oncogenic |
| L636* | Nonsense Mutation | Lung Squamous Cell Carcinoma | Likely Oncogenic |
| Y279* | Nonsense Mutation | Lung Squamous Cell Carcinoma | Likely Oncogenic |
| R882W | Missense Mutation | Cutaneous Melanoma | Likely Oncogenic |
| R93* | Nonsense Mutation | Cutaneous Melanoma | Likely Oncogenic |
| X889_splice | Splice Site | Metaplastic Breast Cancer | Likely Oncogenic |
| L21Afs*10 | Frame Shift Deletion | Breast Invasive Ductal Carcinoma | Likely Oncogenic |
| Y246* | Nonsense Mutation | Breast Invasive Ductal Carcinoma | Likely Oncogenic |
| G909V | Missense Mutation | Esophageal Adenocarcinoma | Likely Oncogenic |
| N894Kfs*14 | Frame Shift Deletion | Esophageal Squamous Cell Carcinoma | Likely Oncogenic |
| P277Sfs*21 | Frame Shift Insertion | Stomach Adenocarcinoma | Likely Oncogenic |
| R1331* | Nonsense Mutation | Colon Adenocarcinoma | Likely Oncogenic |
| Q872* | Nonsense Mutation | Rectal Adenocarcinoma | Likely Oncogenic |
| L122Tfs*4 | Frame Shift Deletion | Serous Ovarian Cancer | Likely Oncogenic |
| Q602* | Nonsense Mutation | Serous Ovarian Cancer | Likely Oncogenic |
| E928Gfs*27 | Frame Shift Insertion | Serous Ovarian Cancer | Likely Oncogenic |
| L996F | Missense Mutation | Serous Ovarian Cancer | Likely Oncogenic |
| Y901C | Missense Mutation | Serous Ovarian Cancer | Likely Oncogenic |
| R882L | Missense Mutation | Serous Ovarian Cancer | Likely Oncogenic |
| W719* | Nonsense Mutation | Serous Ovarian Cancer | Likely Oncogenic |
| H589Qfs*20 | Frame Shift Deletion | Seminoma | Likely Oncogenic |
| S587Ffs*54 | Frame Shift Insertion | Renal Clear Cell Carcinoma | Likely Oncogenic |
| X349_splice | Splice Site | Renal Clear Cell Carcinoma | Likely Oncogenic |
| Q944* | Nonsense Mutation | Cutaneous Melanoma | Likely Oncogenic |
| X807_splice | Splice Site | Chromophobe Renal Cell Carcinoma | Likely Oncogenic |
| S1066Lfs*14 | Frame Shift Deletion | Hepatocellular Carcinoma | Likely Oncogenic |
| R773C | Missense Mutation | Esophageal Adenocarcinoma | Unknown |
| V1094L | Missense Mutation | Breast Invasive Ductal Carcinoma | Unknown |
| P1144S | Missense Mutation | Breast Invasive Ductal Carcinoma | Unknown |
| R1008Q | Missense Mutation | Oligodendroglioma | Unknown |
| R858W | Missense Mutation | Prostate Adenocarcinoma | Unknown |
| G1446E | Missense Mutation | Prostate Adenocarcinoma | Unknown |
| S1066P | Missense Mutation | Prostate Adenocarcinoma | Unknown |
| E774K | Missense Mutation | Prostate Adenocarcinoma | Unknown |
| S148L | Missense Mutation | Bladder Urothelial Carcinoma | Unknown |
| P526H | Missense Mutation | Pancreatic Adenocarcinoma | Unknown |
| P585L | Missense Mutation | Cutaneous Melanoma | Unknown |
| R1008W | Missense Mutation | Lung Squamous Cell Carcinoma | Unknown |
| A1117E | Missense Mutation | Diffuse Type Stomach Adenocarcinoma | Unknown |
| R981H | Missense Mutation | Stomach Adenocarcinoma | Unknown |
| S376G | Missense Mutation | Stomach Adenocarcinoma | Unknown |
| R779C | Missense Mutation | Tubular Stomach Adenocarcinoma | Unknown |
| G923V | Missense Mutation | Stomach Adenocarcinoma | Unknown |
| R378C | Missense Mutation | Stomach Adenocarcinoma | Unknown |
| R779H | Missense Mutation | Diffuse Type Stomach Adenocarcinoma | Unknown |
| R779H | Missense Mutation | Head and Neck Squamous Cell Carcinoma | Unknown |
| M840V | Missense Mutation | Stomach Adenocarcinoma | Unknown |
| D121H | Missense Mutation | Tubular Stomach Adenocarcinoma | Unknown |
| P915S | Missense Mutation | Tubular Stomach Adenocarcinoma | Unknown |
| F986S | Missense Mutation | Head and Neck Squamous Cell Carcinoma | Unknown |
| F1348L | Missense Mutation | Head and Neck Squamous Cell Carcinoma | Unknown |
| S503C | Missense Mutation | Head and Neck Squamous Cell Carcinoma | Unknown |
| R980G | Missense Mutation | Head and Neck Squamous Cell Carcinoma | Unknown |
| K489E | Missense Mutation | Lung Adenocarcinoma | Unknown |
| A993T | Missense Mutation | Lung Adenocarcinoma | Unknown |
| S870C | Missense Mutation | Lung Adenocarcinoma | Unknown |
| V425A | Missense Mutation | Lung Adenocarcinoma | Unknown |
| V425A | Missense Mutation | Lung Adenocarcinoma | Unknown |
| V1367L | Missense Mutation | Lung Adenocarcinoma | Unknown |
| V1367L | Missense Mutation | Lung Adenocarcinoma | Unknown |
| Y327C | Missense Mutation | Lung Adenocarcinoma | Unknown |
| Y327C | Missense Mutation | Lung Adenocarcinoma | Unknown |
| S283L | Missense Mutation | Lung Adenocarcinoma | Unknown |
| S283L | Missense Mutation | Lung Adenocarcinoma | Unknown |
| S265R | Missense Mutation | Lung Adenocarcinoma | Unknown |
| S265R | Missense Mutation | Lung Adenocarcinoma | Unknown |
| K208T | Missense Mutation | Uterine Endometrioid Carcinoma | Unknown |
| P653H | Missense Mutation | Uterine Endometrioid Carcinoma | Unknown |
| P683T | Missense Mutation | Uterine Endometrioid Carcinoma | Unknown |
| G1483R | Missense Mutation | Uterine Serous Carcinoma/Uterine Papillary Serous Carcinoma | Unknown |
| P535S | Missense Mutation | Uterine Endometrioid Carcinoma | Unknown |
| L554_P556del | In Frame Deletion | Uterine Serous Carcinoma/Uterine Papillary Serous Carcinoma | Unknown |
| K853N | Missense Mutation | Uterine Endometrioid Carcinoma | Unknown |
| G677D | Missense Mutation | Uterine Endometrioid Carcinoma | Unknown |
| S475Y | Missense Mutation | Uterine Endometrioid Carcinoma | Unknown |
| F986L | Missense Mutation | Uterine Endometrioid Carcinoma | Unknown |
| R344W | Missense Mutation | Bladder Urothelial Carcinoma | Unknown |
| E189K | Missense Mutation | Bladder Urothelial Carcinoma | Unknown |
| S1329F | Missense Mutation | Bladder Urothelial Carcinoma | Unknown |
| L1323F | Missense Mutation | Bladder Urothelial Carcinoma | Unknown |
| D1100N | Missense Mutation | Renal Clear Cell Carcinoma | Unknown |
| N96K | Missense Mutation | Renal Clear Cell Carcinoma | Unknown |
| L649R | Missense Mutation | Renal Clear Cell Carcinoma | Unknown |
| R449T | Missense Mutation | Lung Squamous Cell Carcinoma | Unknown |
| R449T | Missense Mutation | Lung Squamous Cell Carcinoma | Unknown |
| E471Q | Missense Mutation | Lung Squamous Cell Carcinoma | Unknown |
| E471Q | Missense Mutation | Lung Squamous Cell Carcinoma | Unknown |
| E845D | Missense Mutation | Lung Adenocarcinoma | Unknown |
| P390S | Missense Mutation | Lung Squamous Cell Carcinoma | Unknown |
| G1073A | Missense Mutation | Lung Squamous Cell Carcinoma | Unknown |
| V1367I | Missense Mutation | Lung Adenocarcinoma | Unknown |
| P904L | Missense Mutation | Lung Adenocarcinoma | Unknown |
| K853Q | Missense Mutation | Lung Adenocarcinoma | Unknown |
| K438R | Missense Mutation | Lung Adenocarcinoma | Unknown |
| S323L | Missense Mutation | Lung Adenocarcinoma | Unknown |
| L1457R | Missense Mutation | Lung Squamous Cell Carcinoma | Unknown |
| S593Y | Missense Mutation | Lung Squamous Cell Carcinoma | Unknown |
| R950G | Missense Mutation | Lung Squamous Cell Carcinoma | Unknown |
| S29C | Missense Mutation | Lung Squamous Cell Carcinoma | Unknown |
| R221K | Missense Mutation | Lung Adenocarcinoma | Unknown |
| S889C | Missense Mutation | Lung Squamous Cell Carcinoma | Unknown |
| P428R | Missense Mutation | Lung Squamous Cell Carcinoma | Unknown |
| L1362F | Missense Mutation | Lung Squamous Cell Carcinoma | Unknown |
| R981C | Missense Mutation | Lung Adenocarcinoma | Unknown |
| T893R | Missense Mutation | Lung Adenocarcinoma | Unknown |
| E751K | Missense Mutation | Lung Squamous Cell Carcinoma | Unknown |
| P586S | Missense Mutation | Cutaneous Melanoma | Unknown |
| K41T | Missense Mutation | Breast Invasive Ductal Carcinoma | Unknown |
| D86N | Missense Mutation | Breast Invasive Ductal Carcinoma | Unknown |
| P1091L | Missense Mutation | Tubular Stomach Adenocarcinoma | Unknown |
| R902P | Missense Mutation | Breast Invasive Lobular Carcinoma | Unknown |
| N939S | Missense Mutation | Breast Invasive Ductal Carcinoma | Unknown |
| E1162K | Missense Mutation | Follicular Thyroid Cancer | Unknown |
| L926I | Missense Mutation | Stomach Adenocarcinoma | Unknown |
| L926I | Missense Mutation | Breast Invasive Ductal Carcinoma | Unknown |
| T1353I | Missense Mutation | Intrahepatic Cholangiocarcinoma | Unknown |
| L820I | Missense Mutation | Esophageal Adenocarcinoma | Unknown |
| E1041K | Missense Mutation | Esophageal Squamous Cell Carcinoma | Unknown |
| R1404C | Missense Mutation | Hepatocellular Carcinoma | Unknown |
| D795E | Missense Mutation | Prostate Adenocarcinoma | Unknown |
| P561T | Missense Mutation | Stomach Adenocarcinoma | Unknown |
| A572E | Missense Mutation | Stomach Adenocarcinoma | Unknown |
| Q575E | Missense Mutation | Stomach Adenocarcinoma | Unknown |
| D639V | Missense Mutation | Myxofibrosarcoma | Unknown |
| P515L | Missense Mutation | Undifferentiated Pleomorphic Sarcoma/Malignant Fibrous Histiocytoma/High-Grade Spindle Cell Sarcoma | Unknown |
| K1422N | Missense Mutation | Colon Adenocarcinoma | Unknown |
| E1171K | Missense Mutation | Colon Adenocarcinoma | Unknown |
| F802V | Missense Mutation | Colon Adenocarcinoma | Unknown |
| D502Y | Missense Mutation | Colon Adenocarcinoma | Unknown |
| R722C | Missense Mutation | Colon Adenocarcinoma | Unknown |
| P974L | Missense Mutation | Rectal Adenocarcinoma | Unknown |
| T1014_Q1016del | In Frame Deletion | Serous Ovarian Cancer | Unknown |
| K975E | Missense Mutation | Serous Ovarian Cancer | Likely Neutral |
| T581I | Missense Mutation | Anaplastic Astrocytoma | Unknown |
| P553L | Missense Mutation | Diffuse Large B-Cell Lymphoma | Unknown |
| A618V | Missense Mutation | Head and Neck Squamous Cell Carcinoma | Unknown |
| A284V | Missense Mutation | Hepatocellular Carcinoma | Unknown |
| K211T | Missense Mutation | Pancreatic Adenocarcinoma | Unknown |
| L468Q | Missense Mutation | Papillary Renal Cell Carcinoma | Unknown |
| R1067Q | Missense Mutation | Cutaneous Melanoma | Unknown |
| S1306F | Missense Mutation | Cutaneous Melanoma | Unknown |
| D92A | Missense Mutation | Cutaneous Melanoma | Unknown |
| V793F | Missense Mutation | Cutaneous Melanoma | Unknown |
| P1060L | Missense Mutation | Cutaneous Melanoma | Unknown |
| P961S | Missense Mutation | Cutaneous Melanoma | Unknown |
| P629S | Missense Mutation | Desmoplastic Melanoma | Unknown |
| P1313S | Missense Mutation | Melanoma | Unknown |
| D877Y | Missense Mutation | Intrahepatic Cholangiocarcinoma | Unknown |
| I1131V | Missense Mutation | Diffuse Large B-Cell Lymphoma | Unknown |
| L940V | Missense Mutation | Leiomyosarcoma | Unknown |
| K414N | Missense Mutation | Hepatocellular Carcinoma | Unknown |
| H1413D | Missense Mutation | Pancreatic Adenocarcinoma | Unknown |
| E182del | In Frame Deletion | Lung Squamous Cell Carcinoma | Unknown |
| P1084Q | Missense Mutation | Cutaneous Melanoma | Unknown |
| G677Y | Missense Mutation | Lung Adenocarcinoma | Unknown |
| K852E | Missense Mutation | Lung Adenocarcinoma | Unknown |
| E1263K | Missense Mutation | Lung Squamous Cell Carcinoma | Unknown |

| **CHEK2 Mutation** | **Mutation Type** | **Cancer Type** | **OncoKB Analysis** |
| --- | --- | --- | --- |
| K373E | Missense Mutation | Prostate Adenocarcinoma | Oncogenic |
| K373E | Missense Mutation | Prostate Adenocarcinoma | Oncogenic |
| K373E | Missense Mutation | Prostate Adenocarcinoma | Oncogenic |
| K373E | Missense Mutation | Prostate Adenocarcinoma | Oncogenic |
| K373E | Missense Mutation | Prostate Adenocarcinoma | Oncogenic |
| K373E | Missense Mutation | Prostate Adenocarcinoma | Oncogenic |
| K373E | Missense Mutation | Prostate Adenocarcinoma | Oncogenic |
| K373E | Missense Mutation | Prostate Adenocarcinoma | Oncogenic |
| K373E | Missense Mutation | Prostate Adenocarcinoma | Oncogenic |
| K373E | Missense Mutation | Prostate Adenocarcinoma | Oncogenic |
| K373E | Missense Mutation | Prostate Adenocarcinoma | Oncogenic |
| K373E | Missense Mutation | Prostate Adenocarcinoma | Oncogenic |
| K373E | Missense Mutation | Prostate Adenocarcinoma | Oncogenic |
| K373E | Missense Mutation | Prostate Adenocarcinoma | Oncogenic |
| K373E | Missense Mutation | Prostate Adenocarcinoma | Oncogenic |
| K373E | Missense Mutation | Prostate Adenocarcinoma | Oncogenic |
| K373E | Missense Mutation | Prostate Adenocarcinoma | Oncogenic |
| K373E | Missense Mutation | Prostate Adenocarcinoma | Oncogenic |
| K373E | Missense Mutation | Prostate Adenocarcinoma | Oncogenic |
| K373E | Missense Mutation | Prostate Adenocarcinoma | Oncogenic |
| K373E | Missense Mutation | Prostate Adenocarcinoma | Oncogenic |
| R346C | Missense Mutation | Breast Invasive Ductal Carcinoma | Predicted Oncogenic |
| R346H | Missense Mutation | Serous Ovarian Cancer | Predicted Oncogenic |
| R346G | Missense Mutation | Lung Squamous Cell Carcinoma | Predicted Oncogenic |
| R346G | Missense Mutation | Lung Squamous Cell Carcinoma | Predicted Oncogenic |
| K373E | Missense Mutation | Non-Seminomatous Germ Cell Tumor | Oncogenic |
| K373E | Missense Mutation | Lung Adenocarcinoma | Oncogenic |
| K373E | Missense Mutation | Lung Adenocarcinoma | Oncogenic |
| K373E | Missense Mutation | Bladder Urothelial Carcinoma | Oncogenic |
| K373E | Missense Mutation | Chromophobe Renal Cell Carcinoma | Oncogenic |
| K373E | Missense Mutation | Glioblastoma Multiforme | Oncogenic |
| K373E | Missense Mutation | Glioblastoma Multiforme | Oncogenic |
| K373E | Missense Mutation | Glioblastoma Multiforme | Oncogenic |
| K373E | Missense Mutation | Intrahepatic Cholangiocarcinoma | Oncogenic |
| K373E | Missense Mutation | Pheochromocytoma | Oncogenic |
| K373E | Missense Mutation | Pheochromocytoma | Oncogenic |
| K373E | Missense Mutation | Pheochromocytoma | Oncogenic |
| K373E | Missense Mutation | Pheochromocytoma | Oncogenic |
| K373E | Missense Mutation | Pheochromocytoma | Oncogenic |
| K373E | Missense Mutation | Pheochromocytoma | Oncogenic |
| K373E | Missense Mutation | Pheochromocytoma | Oncogenic |
| K373E | Missense Mutation | Pheochromocytoma | Oncogenic |
| K373E | Missense Mutation | Uterine Carcinosarcoma/Uterine Malignant Mixed Mullerian Tumor | Oncogenic |
| K373E | Missense Mutation | Uterine Carcinosarcoma/Uterine Malignant Mixed Mullerian Tumor | Oncogenic |
| K373E | Missense Mutation | Tubular Stomach Adenocarcinoma | Oncogenic |
| K373E | Missense Mutation | Cutaneous Melanoma | Oncogenic |
| K373E | Missense Mutation | Acral Melanoma | Oncogenic |
| K373E | Missense Mutation | Melanoma | Oncogenic |
| K373E | Missense Mutation | Melanoma | Oncogenic |
| K373E | Missense Mutation | Cutaneous Melanoma | Oncogenic |
| K373E | Missense Mutation | Cutaneous Melanoma | Oncogenic |
| K373E | Missense Mutation | Melanoma | Oncogenic |
| K373E | Missense Mutation | Cutaneous Melanoma | Oncogenic |
| K373E | Missense Mutation | Cutaneous Melanoma | Oncogenic |
| K373E | Missense Mutation | Cutaneous Melanoma | Oncogenic |
| K373E | Missense Mutation | Adrenocortical Carcinoma | Oncogenic |
| K373E | Missense Mutation | Adrenocortical Carcinoma | Oncogenic |
| K373E | Missense Mutation | Renal Clear Cell Carcinoma | Oncogenic |
| K373E | Missense Mutation | Renal Clear Cell Carcinoma | Oncogenic |
| K373E | Missense Mutation | Renal Clear Cell Carcinoma | Oncogenic |
| K373E | Missense Mutation | Renal Clear Cell Carcinoma | Oncogenic |
| K373E | Missense Mutation | Renal Clear Cell Carcinoma | Oncogenic |
| K373E | Missense Mutation | Esophageal Adenocarcinoma | Oncogenic |
| K373E | Missense Mutation | Esophageal Squamous Cell Carcinoma | Oncogenic |
| K373E | Missense Mutation | Head and Neck Squamous Cell Carcinoma | Oncogenic |
| K373E | Missense Mutation | Head and Neck Squamous Cell Carcinoma | Oncogenic |
| K373E | Missense Mutation | Head and Neck Squamous Cell Carcinoma | Oncogenic |
| K373E | Missense Mutation | Head and Neck Squamous Cell Carcinoma | Oncogenic |
| K373E | Missense Mutation | Head and Neck Squamous Cell Carcinoma | Oncogenic |
| K373E | Missense Mutation | Head and Neck Squamous Cell Carcinoma | Oncogenic |
| K373E | Missense Mutation | Head and Neck Squamous Cell Carcinoma | Oncogenic |
| K373E | Missense Mutation | Head and Neck Squamous Cell Carcinoma | Oncogenic |
| K373E | Missense Mutation | Head and Neck Squamous Cell Carcinoma | Oncogenic |
| K373E | Missense Mutation | Head and Neck Squamous Cell Carcinoma | Oncogenic |
| K373E | Missense Mutation | Head and Neck Squamous Cell Carcinoma | Oncogenic |
| K373E | Missense Mutation | Head and Neck Squamous Cell Carcinoma | Oncogenic |
| K373E | Missense Mutation | Head and Neck Squamous Cell Carcinoma | Oncogenic |
| K373E | Missense Mutation | Head and Neck Squamous Cell Carcinoma | Oncogenic |
| K373E | Missense Mutation | Head and Neck Squamous Cell Carcinoma | Oncogenic |
| K373E | Missense Mutation | Head and Neck Squamous Cell Carcinoma | Oncogenic |
| E321* | Nonsense Mutation | Uterine Endometrioid Carcinoma | Likely Oncogenic |
| E321* | Nonsense Mutation | Uterine Endometrioid Carcinoma | Likely Oncogenic |
| X198_splice | Splice Site | Stomach Adenocarcinoma | Likely Oncogenic |
| E275* | Nonsense Mutation | Uterine Endometrioid Carcinoma | Likely Oncogenic |
| E394Kfs*20 | Frame Shift Deletion | Stomach Adenocarcinoma | Likely Oncogenic |
| F202Lfs*3 | Frame Shift Deletion | Stomach Adenocarcinoma | Likely Oncogenic |
| T323Lfs*14 | Frame Shift Deletion | Head and Neck Squamous Cell Carcinoma | Likely Oncogenic |
| L466Ffs*3 | Frame Shift Deletion | Lung Adenocarcinoma | Likely Oncogenic |
| L466Ffs*3 | Frame Shift Deletion | Lung Adenocarcinoma | Likely Oncogenic |
| X514_splice | Splice Site | Uterine Endometrioid Carcinoma | Likely Oncogenic |
| S210* | Nonsense Mutation | Uterine Endometrioid Carcinoma | Likely Oncogenic |
| S356* | Nonsense Mutation | Bladder Urothelial Carcinoma | Likely Oncogenic |
| X365_splice | Splice Site | Lung Squamous Cell Carcinoma | Likely Oncogenic |
| X365_splice | Splice Site | Lung Squamous Cell Carcinoma | Likely Oncogenic |
| R95* | Nonsense Mutation | Adrenocortical Carcinoma | Likely Oncogenic |
| Y390* | Nonsense Mutation | Papillary Thyroid Cancer | Likely Oncogenic |
| S428F | Missense Mutation | Bladder Urothelial Carcinoma | Likely Oncogenic |
| X366_splice | Splice Site | Tubular Stomach Adenocarcinoma | Likely Oncogenic |
| E478* | Nonsense Mutation | Thymoma | Likely Oncogenic |
| X2_splice | Splice Region | Uterine Endometrioid Carcinoma | Likely Oncogenic |
| X420_splice | Splice Site | Uterine Carcinosarcoma/Uterine Malignant Mixed Mullerian Tumor | Likely Oncogenic |
| N290Tfs*14 | Frame Shift Deletion | Hepatocellular Carcinoma | Likely Oncogenic |
| R117G | Missense Mutation | Lung Squamous Cell Carcinoma | Likely Oncogenic |
| D208Ifs*9 | Frame Shift Deletion | Lung Adenocarcinoma | Likely Oncogenic |
| Q100* | Nonsense Mutation | Diffuse Large B-Cell Lymphoma | Likely Oncogenic |
| A98Mfs*13 | Frame Shift Insertion | Diffuse Large B-Cell Lymphoma | Likely Oncogenic |
| L396V | Missense Mutation | Breast Invasive Ductal Carcinoma | Unknown |
| L396V | Missense Mutation | Lung Squamous Cell Carcinoma | Unknown |
| D203E | Missense Mutation | Breast Invasive Ductal Carcinoma | Unknown |
| A480T | Missense Mutation | Oligodendroglioma | Unknown |
| A392V | Missense Mutation | Lung Adenocarcinoma | Unknown |
| A392V | Missense Mutation | Lung Adenocarcinoma | Unknown |
| L226F | Missense Mutation | Undifferentiated Pleomorphic Sarcoma/Malignant Fibrous Histiocytoma/High-Grade Spindle Cell Sarcoma | Unknown |
| L543S | Missense Mutation | Stomach Adenocarcinoma | Unknown |
| A541G | Missense Mutation | Stomach Adenocarcinoma | Unknown |
| Q487R | Missense Mutation | Stomach Adenocarcinoma | Unknown |
| N154S | Missense Mutation | Stomach Adenocarcinoma | Unknown |
| R474H | Missense Mutation | Signet Ring Cell Carcinoma of the Stomach | Unknown |
| R519G | Missense Mutation | Glioblastoma Multiforme | Unknown |
| R519G | Missense Mutation | Head and Neck Squamous Cell Carcinoma | Unknown |
| N166S | Missense Mutation | Head and Neck Squamous Cell Carcinoma | Unknown |
| T532I | Missense Mutation | Head and Neck Squamous Cell Carcinoma | Unknown |
| W93L | Missense Mutation | Lung Adenocarcinoma | Unknown |
| A392S | Missense Mutation | Lung Adenocarcinoma | Unknown |
| N185Y | Missense Mutation | Lung Adenocarcinoma | Unknown |
| C18S | Missense Mutation | Lung Adenocarcinoma | Unknown |
| C18S | Missense Mutation | Lung Adenocarcinoma | Unknown |
| T138A | Missense Mutation | Lung Adenocarcinoma | Unknown |
| T138A | Missense Mutation | Lung Adenocarcinoma | Unknown |
| I276S | Missense Mutation | Uterine Endometrioid Carcinoma | Unknown |
| D134Y | Missense Mutation | Uterine Endometrioid Carcinoma | Unknown |
| K135T | Missense Mutation | Uterine Endometrioid Carcinoma | Unknown |
| L354V | Missense Mutation | Uterine Endometrioid Carcinoma | Unknown |
| G102V | Missense Mutation | Uterine Endometrioid Carcinoma | Unknown |
| E351D | Missense Mutation | Uterine Endometrioid Carcinoma | Unknown |
| K279T | Missense Mutation | Uterine Endometrioid Carcinoma | Unknown |
| A247T | Missense Mutation | Uterine Serous Carcinoma/Uterine Papillary Serous Carcinoma | Unknown |
| E377K | Missense Mutation | Bladder Urothelial Carcinoma | Unknown |
| H339Y | Missense Mutation | Bladder Urothelial Carcinoma | Unknown |
| V109G | Missense Mutation | Renal Clear Cell Carcinoma | Unknown |
| Y404C | Missense Mutation | Lung Squamous Cell Carcinoma | Unknown |
| Y404C | Missense Mutation | Lung Squamous Cell Carcinoma | Unknown |
| Q433E | Missense Mutation | Bladder Urothelial Carcinoma | Unknown |
| L380V | Missense Mutation | Lung Adenocarcinoma | Unknown |
| A257S | Missense Mutation | Lung Squamous Cell Carcinoma | Unknown |
| H282D | Missense Mutation | Lung Squamous Cell Carcinoma | Unknown |
| L363F | Missense Mutation | Lung Adenocarcinoma | Unknown |
| G386V | Missense Mutation | Lung Adenocarcinoma | Unknown |
| D207H | Missense Mutation | Lung Squamous Cell Carcinoma | Unknown |
| S16R | Missense Mutation | Lung Adenocarcinoma | Unknown |
| Y337D | Missense Mutation | Lung Squamous Cell Carcinoma | Unknown |
| R195K | Missense Mutation | Lung Adenocarcinoma | Unknown |
| E308Q | Missense Mutation | Breast Invasive Lobular Carcinoma | Unknown |
| D77H | Missense Mutation | Breast Invasive Lobular Carcinoma | Unknown |
| R535H | Missense Mutation | Thymoma | Unknown |
| R535H | Missense Mutation | Esophageal Adenocarcinoma | Unknown |
| R535H | Missense Mutation | Intrahepatic Cholangiocarcinoma | Unknown |
| F103L | Missense Mutation | Esophageal Adenocarcinoma | Unknown |
| H345L | Missense Mutation | Esophageal Squamous Cell Carcinoma | Unknown |
| R3Q | Missense Mutation | Hepatocellular Carcinoma | Unknown |
| D111G | Missense Mutation | Mucinous Stomach Adenocarcinoma | Unknown |
| N186S | Missense Mutation | Papillary Thyroid Cancer | Unknown |
| L391F | Missense Mutation | Follicular Thyroid Cancer | Unknown |
| L462I | Missense Mutation | Papillary Thyroid Cancer | Unknown |
| H143Q | Missense Mutation | Papillary Thyroid Cancer | Unknown |
| E70G | Missense Mutation | Intrahepatic Cholangiocarcinoma | Unknown |
| S24C | Missense Mutation | Hepatocellular Carcinoma | Unknown |
| E81A | Missense Mutation | Hepatocellular Carcinoma | Unknown |
| S56R | Missense Mutation | Pancreatic Adenocarcinoma | Unknown |
| P90S | Missense Mutation | Cutaneous Melanoma | Unknown |
| N184I | Missense Mutation | Adrenocortical Carcinoma | Unknown |
| R406H | Missense Mutation | Chromophobe Renal Cell Carcinoma | Unknown |
| F144L | Missense Mutation | Colon Adenocarcinoma | Unknown |
| S372F | Missense Mutation | Head and Neck Squamous Cell Carcinoma | Unknown |
| A527V | Missense Mutation | Cutaneous Melanoma | Unknown |
| F495I | Missense Mutation | Lentigo Maligna Melanoma | Unknown |
| H143N | Missense Mutation | Papillary Renal Cell Carcinoma | Unknown |
| A407T | Missense Mutation | Undifferentiated Pleomorphic Sarcoma/Malignant Fibrous Histiocytoma/High-Grade Spindle Cell Sarcoma | Unknown |
| L338R | Missense Mutation | Uterine Carcinosarcoma/Uterine Malignant Mixed Mullerian Tumor | Unknown |
| S55P | Missense Mutation | Hepatocellular Carcinoma | Unknown |
| E263K | Missense Mutation | Diffuse Large B-Cell Lymphoma | Unknown |

| **DMC1 Mutation** | **Mutation Type** | **Cancer Type** | **OncoKB Analysis** |
| --- | --- | --- | --- |
| T277I | Missense Mutation | Breast Invasive Ductal Carcinoma | Unknown |
| R166H | Missense Mutation | Anaplastic Oligoastrocytoma | Unknown |
| H211Y | Missense Mutation | Cutaneous Melanoma | Unknown |
| R230* | Nonsense Mutation | Cutaneous Melanoma | Unknown |
| R176C | Missense Mutation | Stomach Adenocarcinoma | Unknown |
| R176C | Missense Mutation | Rectal Adenocarcinoma | Unknown |
| K245Nfs*22 | Frame Shift Deletion | Stomach Adenocarcinoma | Unknown |
| K245Nfs*22 | Frame Shift Deletion | Stomach Adenocarcinoma | Unknown |
| G64R | Missense Mutation | Stomach Adenocarcinoma | Unknown |
| T47A | Missense Mutation | Stomach Adenocarcinoma | Unknown |
| V34M | Missense Mutation | Lung Adenocarcinoma | Unknown |
| V34M | Missense Mutation | Lung Adenocarcinoma | Unknown |
| V34M | Missense Mutation | Colon Adenocarcinoma | Unknown |
| F85L | Missense Mutation | Uterine Endometrioid Carcinoma | Unknown |
| F85L | Missense Mutation | Colon Adenocarcinoma | Unknown |
| D160H | Missense Mutation | Head and Neck Squamous Cell Carcinoma | Unknown |
| D204Y | Missense Mutation | Lung Adenocarcinoma | Unknown |
| R169C | Missense Mutation | Uterine Endometrioid Carcinoma | Unknown |
| R304* | Nonsense Mutation | Uterine Endometrioid Carcinoma | Unknown |
| T130I | Missense Mutation | Uterine Endometrioid Carcinoma | Unknown |
| A247V | Missense Mutation | Uterine Endometrioid Carcinoma | Unknown |
| E325K | Missense Mutation | Bladder Urothelial Carcinoma | Unknown |
| M200I | Missense Mutation | Lung Squamous Cell Carcinoma | Unknown |
| R129C | Missense Mutation | Cutaneous Melanoma | Unknown |
| R129C | Missense Mutation | Cutaneous Melanoma | Unknown |
| R129C | Missense Mutation | Cutaneous Melanoma | Unknown |
| R129C | Missense Mutation | Cutaneous Melanoma | Unknown |
| H137Y | Missense Mutation | Breast Invasive Ductal Carcinoma | Unknown |
| H211Q | Missense Mutation | Esophageal Adenocarcinoma | Unknown |
| T55I | Missense Mutation | Esophageal Adenocarcinoma | Unknown |
| R192H | Missense Mutation | Mucinous Stomach Adenocarcinoma | Unknown |
| R171* | Frame Shift Insertion | Stomach Adenocarcinoma | Unknown |
| X17_splice | Splice Site | Colon Adenocarcinoma | Unknown |
| X141_splice | Splice Site | Stomach Adenocarcinoma | Unknown |
| F89Lfs*24 | Frame Shift Deletion | Papillary Renal Cell Carcinoma | Unknown |
| F280I | Missense Mutation | Cutaneous Melanoma | Unknown |
| G235C | Missense Mutation | Cutaneous Melanoma | Unknown |
| Q254* | Nonsense Mutation | Cutaneous Melanoma | Unknown |
| F233Y | Missense Mutation | Cutaneous Melanoma | Unknown |
| L220 | Splice Region | Cutaneous Melanoma | Unknown |
| T279 | Splice Region | Cutaneous Melanoma | Unknown |

| **FAAP20 Mutation** | **Mutation Type** | **Cancer Type** | **OncoKB Analysis** |
| --- | --- | --- | --- |
| V47A | Missense Mutation | Breast Invasive Ductal Carcinoma | Unknown |
| G106R | Missense Mutation | Glioblastoma Multiforme | Unknown |
| P72Rfs*87 | Frame Shift Deletion | Stomach Adenocarcinoma | Unknown |
| R26C | Missense Mutation | Uterine Serous Carcinoma/Uterine Papillary Serous Carcinoma | Unknown |
| L44Q | Missense Mutation | Bladder Urothelial Carcinoma | Unknown |
| W180R | Missense Mutation | Bladder Urothelial Carcinoma | Unknown |
| E176A | Missense Mutation | Renal Clear Cell Carcinoma | Unknown |
| E176A | Missense Mutation | Renal Clear Cell Carcinoma | Unknown |
| A155T | Missense Mutation | Lung Squamous Cell Carcinoma | Unknown |
| V178M | Missense Mutation | Mucinous Stomach Adenocarcinoma | Unknown |
| G33Vfs*15 | Frame Shift Deletion | Diffuse Type Stomach Adenocarcinoma | Unknown |
| R121C | Missense Mutation | Myxofibrosarcoma | Unknown |
| A142E | Missense Mutation | Papillary Renal Cell Carcinoma | Unknown |
| H105L | Missense Mutation | Cutaneous Melanoma | Unknown |
| P22S | Missense Mutation | Cutaneous Melanoma | Unknown |

| **FAN1 Mutation** | **Mutation Type** | **Cancer Type** | **OncoKB Analysis** |
| --- | --- | --- | --- |
| R377L | Missense Mutation | Lung Adenocarcinoma | Unknown |
| R377L | Missense Mutation | Lung Adenocarcinoma | Unknown |
| M1? | Translation Start Site | Lung Squamous Cell Carcinoma | Unknown |
| V308Cfs*5 | Frame Shift Deletion | Breast Invasive Lobular Carcinoma | Unknown |
| C47R | Missense Mutation | Glioblastoma Multiforme | Unknown |
| S553L | Missense Mutation | Cutaneous Melanoma | Unknown |
| S553L | Missense Mutation | Mucinous Adenocarcinoma of the Colon and Rectum | Unknown |
| A801V | Missense Mutation | Desmoplastic Melanoma | Unknown |
| R581* | Nonsense Mutation | Stomach Adenocarcinoma | Unknown |
| A261V | Missense Mutation | Stomach Adenocarcinoma | Unknown |
| A261V | Missense Mutation | Lung Adenocarcinoma | Unknown |
| A261V | Missense Mutation | Lung Adenocarcinoma | Unknown |
| T609A | Missense Mutation | Stomach Adenocarcinoma | Unknown |
| F762S | Missense Mutation | Stomach Adenocarcinoma | Unknown |
| P8del | In Frame Deletion | Stomach Adenocarcinoma | Unknown |
| K313E | Missense Mutation | Uterine Endometrioid Carcinoma | Unknown |
| K313E | Missense Mutation | Stomach Adenocarcinoma | Unknown |
| C812Y | Missense Mutation | Tubular Stomach Adenocarcinoma | Unknown |
| A686V | Missense Mutation | Tubular Stomach Adenocarcinoma | Unknown |
| D857N | Missense Mutation | Cutaneous Melanoma | Unknown |
| D857N | Missense Mutation | Head and Neck Squamous Cell Carcinoma | Unknown |
| E975G | Missense Mutation | Head and Neck Squamous Cell Carcinoma | Unknown |
| R697Nfs*5 | Frame Shift Deletion | Head and Neck Squamous Cell Carcinoma | Unknown |
| D328H | Missense Mutation | Lung Adenocarcinoma | Unknown |
| D328H | Missense Mutation | Lung Adenocarcinoma | Unknown |
| T245A | Missense Mutation | Uterine Endometrioid Carcinoma | Unknown |
| S160P | Missense Mutation | Uterine Endometrioid Carcinoma | Unknown |
| G233R | Missense Mutation | Uterine Endometrioid Carcinoma | Unknown |
| R668G | Missense Mutation | Uterine Endometrioid Carcinoma | Unknown |
| E895K | Missense Mutation | Uterine Endometrioid Carcinoma | Unknown |
| V97I | Missense Mutation | Uterine Serous Carcinoma/Uterine Papillary Serous Carcinoma | Unknown |
| P541H | Missense Mutation | Uterine Endometrioid Carcinoma | Unknown |
| S220C | Missense Mutation | Bladder Urothelial Carcinoma | Unknown |
| E62G | Missense Mutation | Bladder Urothelial Carcinoma | Unknown |
| P894S | Missense Mutation | Renal Clear Cell Carcinoma | Unknown |
| S32L | Missense Mutation | Cutaneous Melanoma | Unknown |
| T511N | Missense Mutation | Lung Adenocarcinoma | Unknown |
| Q1014H | Missense Mutation | Lung Squamous Cell Carcinoma | Unknown |
| E291D | Missense Mutation | Lung Squamous Cell Carcinoma | Unknown |
| G663R | Missense Mutation | Lung Adenocarcinoma | Unknown |
| N578I | Missense Mutation | Lung Adenocarcinoma | Unknown |
| E438D | Missense Mutation | Lung Adenocarcinoma | Unknown |
| G833C | Missense Mutation | Lung Squamous Cell Carcinoma | Unknown |
| S20I | Missense Mutation | Prostate Adenocarcinoma | Unknown |
| S20I | Missense Mutation | Lung Squamous Cell Carcinoma | Unknown |
| R969S | Missense Mutation | Lung Adenocarcinoma | Unknown |
| F762L | Missense Mutation | Lung Adenocarcinoma | Unknown |
| R12Gfs*45 | Frame Shift Deletion | Stomach Adenocarcinoma | Unknown |
| R12Gfs*45 | Frame Shift Deletion | Cutaneous Melanoma | Unknown |
| A999Vfs*3 | Frame Shift Deletion | Breast Invasive Ductal Carcinoma | Unknown |
| F584I | Missense Mutation | Breast Invasive Ductal Carcinoma | Unknown |
| D329V | Missense Mutation | Breast Invasive Lobular Carcinoma | Unknown |
| Q678H | Missense Mutation | Breast Invasive Lobular Carcinoma | Unknown |
| E993K | Missense Mutation | Breast Invasive Ductal Carcinoma | Unknown |
| S586R | Missense Mutation | Breast Invasive Ductal Carcinoma | Unknown |
| L722P | Missense Mutation | Esophageal Adenocarcinoma | Unknown |
| H985R | Missense Mutation | Prostate Adenocarcinoma | Unknown |
| S354N | Missense Mutation | Tubular Stomach Adenocarcinoma | Unknown |
| V382del | In Frame Deletion | Stomach Adenocarcinoma | Unknown |
| T432A | Missense Mutation | Colon Adenocarcinoma | Unknown |
| I125V | Missense Mutation | Colon Adenocarcinoma | Unknown |
| V1006M | Missense Mutation | Mucinous Adenocarcinoma of the Colon and Rectum | Unknown |
| T905M | Missense Mutation | Mucinous Adenocarcinoma of the Colon and Rectum | Unknown |
| E476G | Missense Mutation | Astrocytoma | Unknown |
| D776Y | Missense Mutation | Adrenocortical Carcinoma | Unknown |
| P475A | Missense Mutation | Head and Neck Squamous Cell Carcinoma | Unknown |
| S466F | Missense Mutation | Head and Neck Squamous Cell Carcinoma | Unknown |
| R912G | Missense Mutation | Hepatocellular Carcinoma | Unknown |
| D68V | Missense Mutation | Hepatocellular Carcinoma | Unknown |
| P127H | Missense Mutation | Hepatocellular Carcinoma | Unknown |
| H324Q | Missense Mutation | Papillary Renal Cell Carcinoma | Unknown |
| G491R | Missense Mutation | Cutaneous Melanoma | Unknown |
| L42P | Missense Mutation | Cutaneous Melanoma | Unknown |
| E249D | Missense Mutation | Uterine Carcinosarcoma/Uterine Malignant Mixed Mullerian Tumor | Unknown |
| T609M | Missense Mutation | Uterine Carcinosarcoma/Uterine Malignant Mixed Mullerian Tumor | Unknown |
| V974G | Missense Mutation | Breast Invasive Ductal Carcinoma | Unknown |
| V780= | Splice Region | Non-Seminomatous Germ Cell Tumor | Unknown |
| K243R | Missense Mutation | Seminoma | Unknown |
| A177V | Missense Mutation | Lung Squamous Cell Carcinoma | Unknown |
| R879K | Missense Mutation | Melanoma | Unknown |
| A40T | Missense Mutation | Diffuse Large B-Cell Lymphoma | Unknown |
| E561* | Nonsense Mutation | Lung Adenocarcinoma | Unknown |
| R952Q | Missense Mutation | Lung Adenocarcinoma | Unknown |

| **FANCD2 Mutation** | **Mutation Type** | **Cancer Type** | **OncoKB Analysis** |
| --- | --- | --- | --- |
| E766* | Nonsense Mutation | Breast Invasive Ductal Carcinoma | Likely Oncogenic |
| R1273* | Nonsense Mutation | Mucinous Adenocarcinoma of the Colon and Rectum | Likely Oncogenic |
| C1130* | Nonsense Mutation | Prostate Adenocarcinoma | Likely Oncogenic |
| R408* | Nonsense Mutation | Cutaneous Melanoma | Likely Oncogenic |
| P963Lfs*18 | Frame Shift Deletion | Stomach Adenocarcinoma | Likely Oncogenic |
| F162Lfs*19 | Frame Shift Deletion | Stomach Adenocarcinoma | Likely Oncogenic |
| X869_splice | Splice Site | Stomach Adenocarcinoma | Likely Oncogenic |
| Q1395* | Nonsense Mutation | Diffuse Type Stomach Adenocarcinoma | Likely Oncogenic |
| X426_splice | Splice Site | Renal Clear Cell Carcinoma | Likely Oncogenic |
| X426_splice | Splice Site | Renal Clear Cell Carcinoma | Likely Oncogenic |
| X426_splice | Splice Site | Renal Clear Cell Carcinoma | Likely Oncogenic |
| X426_splice | Splice Site | Renal Clear Cell Carcinoma | Likely Oncogenic |
| X426_splice | Splice Site | Renal Clear Cell Carcinoma | Likely Oncogenic |
| X426_splice | Splice Site | Renal Clear Cell Carcinoma | Likely Oncogenic |
| X426_splice | Splice Site | Renal Clear Cell Carcinoma | Likely Oncogenic |
| X426_splice | Splice Site | Renal Clear Cell Carcinoma | Likely Oncogenic |
| X426_splice | Splice Site | Renal Clear Cell Carcinoma | Likely Oncogenic |
| X426_splice | Splice Site | Renal Clear Cell Carcinoma | Likely Oncogenic |
| X426_splice | Splice Site | Head and Neck Squamous Cell Carcinoma | Likely Oncogenic |
| X426_splice | Splice Site | Head and Neck Squamous Cell Carcinoma | Likely Oncogenic |
| R530* | Nonsense Mutation | Uterine Endometrioid Carcinoma | Likely Oncogenic |
| E1210* | Nonsense Mutation | Uterine Endometrioid Carcinoma | Likely Oncogenic |
| E230* | Nonsense Mutation | Uterine Endometrioid Carcinoma | Likely Oncogenic |
| Q440* | Nonsense Mutation | Breast Invasive Ductal Carcinoma | Likely Oncogenic |
| G841Efs*8 | Frame Shift Deletion | Esophageal Adenocarcinoma | Likely Oncogenic |
| E742* | Nonsense Mutation | Esophageal Adenocarcinoma | Likely Oncogenic |
| Q527* | Nonsense Mutation | Tubular Stomach Adenocarcinoma | Likely Oncogenic |
| Q146* | Nonsense Mutation | Colon Adenocarcinoma | Likely Oncogenic |
| E95* | Nonsense Mutation | Rectal Adenocarcinoma | Likely Oncogenic |
| E650* | Nonsense Mutation | Serous Ovarian Cancer | Likely Oncogenic |
| L426= | Splice Region | Stomach Adenocarcinoma | Likely Oncogenic |
| L1111= | Splice Region | Bladder Urothelial Carcinoma | Likely Oncogenic |
| Y520* | Nonsense Mutation | Diffuse Large B-Cell Lymphoma | Likely Oncogenic |
| X1428_splice | Splice Site | Cutaneous Melanoma | Likely Oncogenic |
| X1427_splice | Splice Region | Cutaneous Melanoma | Likely Oncogenic |
| R926* | Nonsense Mutation | Pancreatic Adenocarcinoma | Likely Oncogenic |
| N392Ifs*12 | Frame Shift Deletion | Lung Squamous Cell Carcinoma | Likely Oncogenic |
| D753N | Missense Mutation | Bladder Urothelial Carcinoma | Unknown |
| Q802H | Missense Mutation | Adrenocortical Carcinoma | Unknown |
| Q802H | Missense Mutation | Adrenocortical Carcinoma | Unknown |
| Q802H | Missense Mutation | Adrenocortical Carcinoma | Unknown |
| Q802H | Missense Mutation | Adrenocortical Carcinoma | Unknown |
| Q802H | Missense Mutation | Metaplastic Breast Cancer | Unknown |
| Q802H | Missense Mutation | Renal Clear Cell Carcinoma | Unknown |
| Q802H | Missense Mutation | Renal Clear Cell Carcinoma | Unknown |
| Q802H | Missense Mutation | Esophageal Squamous Cell Carcinoma | Unknown |
| Q802H | Missense Mutation | Esophageal Adenocarcinoma | Unknown |
| Q802H | Missense Mutation | Esophageal Adenocarcinoma | Unknown |
| Q802H | Missense Mutation | Intrahepatic Cholangiocarcinoma | Unknown |
| A731V | Missense Mutation | Breast Invasive Ductal Carcinoma | Unknown |
| L699F | Missense Mutation | Breast Invasive Ductal Carcinoma | Unknown |
| R735Q | Missense Mutation | Chromophobe Renal Cell Carcinoma | Unknown |
| R1299C | Missense Mutation | Mucinous Adenocarcinoma of the Colon and Rectum | Unknown |
| S780L | Missense Mutation | Glioblastoma Multiforme | Unknown |
| C801R | Missense Mutation | Glioblastoma Multiforme | Unknown |
| E369G | Missense Mutation | Oligoastrocytoma | Unknown |
| F258V | Missense Mutation | Oligoastrocytoma | Unknown |
| P672L | Missense Mutation | Pancreatic Adenocarcinoma | Unknown |
| K396N | Missense Mutation | Uterine Endometrioid Carcinoma | Unknown |
| A291T | Missense Mutation | Stomach Adenocarcinoma | Unknown |
| I1177V | Missense Mutation | Stomach Adenocarcinoma | Unknown |
| L270S | Missense Mutation | Stomach Adenocarcinoma | Unknown |
| E217K | Missense Mutation | Stomach Adenocarcinoma | Unknown |
| S1195N | Missense Mutation | Stomach Adenocarcinoma | Unknown |
| Q1010R | Missense Mutation | Head and Neck Squamous Cell Carcinoma | Unknown |
| S250T | Missense Mutation | Head and Neck Squamous Cell Carcinoma | Unknown |
| A540P | Missense Mutation | Lung Adenocarcinoma | Unknown |
| K53Q | Missense Mutation | Lung Adenocarcinoma | Unknown |
| E14D | Missense Mutation | Lung Adenocarcinoma | Unknown |
| A600G | Missense Mutation | Lung Adenocarcinoma | Unknown |
| A600G | Missense Mutation | Lung Adenocarcinoma | Unknown |
| C1309F | Missense Mutation | Lung Adenocarcinoma | Unknown |
| C1309F | Missense Mutation | Lung Adenocarcinoma | Unknown |
| L1291M | Missense Mutation | Uterine Endometrioid Carcinoma | Unknown |
| E1445A | Missense Mutation | Uterine Endometrioid Carcinoma | Unknown |
| C432R | Missense Mutation | Uterine Endometrioid Carcinoma | Unknown |
| A1373D | Missense Mutation | Uterine Endometrioid Carcinoma | Unknown |
| R870K | Missense Mutation | Uterine Endometrioid Carcinoma | Unknown |
| T1351M | Missense Mutation | Uterine Endometrioid Carcinoma | Unknown |
| R735W | Missense Mutation | Uterine Endometrioid Carcinoma | Unknown |
| S433L | Missense Mutation | Lentigo Maligna Melanoma | Unknown |
| S433L | Missense Mutation | Bladder Urothelial Carcinoma | Unknown |
| L497V | Missense Mutation | Bladder Urothelial Carcinoma | Unknown |
| E1262D | Missense Mutation | Bladder Urothelial Carcinoma | Unknown |
| F161V | Missense Mutation | Bladder Urothelial Carcinoma | Unknown |
| R5T | Missense Mutation | Bladder Urothelial Carcinoma | Unknown |
| L312W | Missense Mutation | Bladder Urothelial Carcinoma | Unknown |
| G685A | Missense Mutation | Bladder Urothelial Carcinoma | Unknown |
| N746K | Missense Mutation | Bladder Urothelial Carcinoma | Unknown |
| G685R | Missense Mutation | Colon Adenocarcinoma | Unknown |
| E1243Q | Missense Mutation | Lung Squamous Cell Carcinoma | Unknown |
| A1159V | Missense Mutation | Lung Adenocarcinoma | Unknown |
| G1043C | Missense Mutation | Lung Squamous Cell Carcinoma | Unknown |
| S1257W | Missense Mutation | Lung Adenocarcinoma | Unknown |
| I196M | Missense Mutation | Lung Squamous Cell Carcinoma | Unknown |
| F664L | Missense Mutation | Lung Squamous Cell Carcinoma | Unknown |
| P679S | Missense Mutation | Lung Squamous Cell Carcinoma | Unknown |
| D1217N | Missense Mutation | Lung Squamous Cell Carcinoma | Unknown |
| S452L | Missense Mutation | Cutaneous Melanoma | Unknown |
| S452L | Missense Mutation | Cutaneous Melanoma | Unknown |
| L972F | Missense Mutation | Breast Invasive Ductal Carcinoma | Unknown |
| R1381T | Missense Mutation | Breast Invasive Lobular Carcinoma | Unknown |
| K871N | Missense Mutation | Chromophobe Renal Cell Carcinoma | Unknown |
| R870S | Missense Mutation | Breast Invasive Ductal Carcinoma | Unknown |
| L1134V | Missense Mutation | Breast Invasive Ductal Carcinoma | Unknown |
| M507V | Missense Mutation | Papillary Thyroid Cancer | Unknown |
| G713D | Missense Mutation | Papillary Thyroid Cancer | Unknown |
| V670A | Missense Mutation | Papillary Thyroid Cancer | Unknown |
| V725M | Missense Mutation | Prostate Adenocarcinoma | Unknown |
| Q1080E | Missense Mutation | Esophageal Adenocarcinoma | Unknown |
| K828T | Missense Mutation | Esophageal Adenocarcinoma | Unknown |
| P852S | Missense Mutation | Mucinous Stomach Adenocarcinoma | Unknown |
| F1275L | Missense Mutation | Tubular Stomach Adenocarcinoma | Unknown |
| L157F | Missense Mutation | Mucinous Stomach Adenocarcinoma | Unknown |
| V988D | Missense Mutation | Diffuse Type Stomach Adenocarcinoma | Unknown |
| S1287I | Missense Mutation | Colon Adenocarcinoma | Unknown |
| R119C | Missense Mutation | Colon Adenocarcinoma | Unknown |
| P593L | Missense Mutation | Mucinous Adenocarcinoma of the Colon and Rectum | Unknown |
| D522G | Missense Mutation | Rectal Adenocarcinoma | Unknown |
| S898Y | Missense Mutation | Rectal Adenocarcinoma | Unknown |
| H1014N | Missense Mutation | Rectal Adenocarcinoma | Unknown |
| L436M | Missense Mutation | Bladder Urothelial Carcinoma | Unknown |
| F843I | Missense Mutation | Breast Invasive Ductal Carcinoma | Unknown |
| R174Q | Missense Mutation | Diffuse Large B-Cell Lymphoma | Unknown |
| R185T | Missense Mutation | Head and Neck Squamous Cell Carcinoma | Unknown |
| Q544H | Missense Mutation | Head and Neck Squamous Cell Carcinoma | Unknown |
| S437L | Missense Mutation | Head and Neck Squamous Cell Carcinoma | Unknown |
| P1311S | Missense Mutation | Pleural Mesothelioma, Biphasic Type | Unknown |
| P1311S | Missense Mutation | Head and Neck Squamous Cell Carcinoma | Unknown |
| G1343E | Missense Mutation | Hepatocellular Carcinoma | Unknown |
| L51R | Missense Mutation | Hepatocellular Carcinoma | Unknown |
| M581T | Missense Mutation | Hepatocellular Carcinoma | Unknown |
| D430N | Missense Mutation | Cutaneous Melanoma | Unknown |
| G1101R | Missense Mutation | Cutaneous Melanoma | Unknown |
| S1157F | Missense Mutation | Cutaneous Melanoma | Unknown |
| L428F | Missense Mutation | Cutaneous Melanoma | Unknown |
| M197I | Missense Mutation | Cutaneous Melanoma | Unknown |
| L456F | Missense Mutation | Cutaneous Melanoma | Unknown |
| S1404F | Missense Mutation | Cutaneous Melanoma | Unknown |
| P625S | Missense Mutation | Head and Neck Squamous Cell Carcinoma | Unknown |
| E376K | Missense Mutation | Head and Neck Squamous Cell Carcinoma | Unknown |
| R355K | Missense Mutation | Head and Neck Squamous Cell Carcinoma | Unknown |
| F1223L | Missense Mutation | Melanoma | Unknown |
| E687K | Missense Mutation | Lentigo Maligna Melanoma | Unknown |
| E642K | Missense Mutation | Cutaneous Melanoma | Unknown |
| E1438G | Missense Mutation | Uterine Carcinosarcoma/Uterine Malignant Mixed Mullerian Tumor | Unknown |
| V427A | Missense Mutation | Cutaneous Melanoma | Unknown |
| G484R | Missense Mutation | Cutaneous Melanoma | Unknown |
| E1173D | Missense Mutation | Breast | Unknown |

| **FANCE Mutation** | **Mutation Type** | **Cancer Type** | **OncoKB Analysis** |
| --- | --- | --- | --- |
| H236Y | Missense Mutation | Serous Ovarian Cancer | Unknown |
| R200C | Missense Mutation | Signet Ring Cell Carcinoma of the Stomach | Unknown |
| R141Q | Missense Mutation | Uterine Endometrioid Carcinoma | Unknown |
| R141Q | Missense Mutation | Uterine Endometrioid Carcinoma | Unknown |
| R200H | Missense Mutation | Uterine Endometrioid Carcinoma | Unknown |
| V311Sfs*2 | Frame Shift Insertion | Stomach Adenocarcinoma | Unknown |
| V311Sfs*2 | Frame Shift Insertion | Stomach Adenocarcinoma | Unknown |
| V311Sfs*2 | Frame Shift Insertion | Renal Clear Cell Carcinoma | Unknown |
| V311Sfs*2 | Frame Shift Insertion | Renal Clear Cell Carcinoma | Unknown |
| V311Sfs*2 | Frame Shift Insertion | Renal Clear Cell Carcinoma | Unknown |
| V311Sfs*2 | Frame Shift Insertion | Renal Clear Cell Carcinoma | Unknown |
| V311Sfs*2 | Frame Shift Insertion | Renal Clear Cell Carcinoma | Unknown |
| V311Sfs*2 | Frame Shift Insertion | Renal Clear Cell Carcinoma | Unknown |
| V311Sfs*2 | Frame Shift Insertion | Renal Clear Cell Carcinoma | Unknown |
| V311Sfs*2 | Frame Shift Insertion | Renal Clear Cell Carcinoma | Unknown |
| V311Sfs*2 | Frame Shift Insertion | Renal Clear Cell Carcinoma | Unknown |
| V311Sfs*2 | Frame Shift Insertion | Renal Clear Cell Carcinoma | Unknown |
| V311Sfs*2 | Frame Shift Insertion | Renal Clear Cell Carcinoma | Unknown |
| V311Sfs*2 | Frame Shift Insertion | Renal Clear Cell Carcinoma | Unknown |
| V311Sfs*2 | Frame Shift Insertion | Renal Clear Cell Carcinoma | Unknown |
| V311Sfs*2 | Frame Shift Insertion | Renal Clear Cell Carcinoma | Unknown |
| V311Sfs*2 | Frame Shift Insertion | Renal Clear Cell Carcinoma | Unknown |
| V311Sfs*2 | Frame Shift Insertion | Renal Clear Cell Carcinoma | Unknown |
| V311Sfs*2 | Frame Shift Insertion | Renal Clear Cell Carcinoma | Unknown |
| V311Sfs*2 | Frame Shift Insertion | Renal Clear Cell Carcinoma | Unknown |
| V311Sfs*2 | Frame Shift Insertion | Esophageal Adenocarcinoma | Unknown |
| P397S | Missense Mutation | Cutaneous Melanoma | Unknown |
| R233K | Missense Mutation | Breast Invasive Ductal Carcinoma | Unknown |
| R92Q | Missense Mutation | Oligoastrocytoma | Unknown |
| G246del | In Frame Deletion | Diffuse Large B-Cell Lymphoma | Unknown |
| R221Q | Missense Mutation | Stomach Adenocarcinoma | Unknown |
| R134C | Missense Mutation | Stomach Adenocarcinoma | Unknown |
| Q323H | Missense Mutation | Lung Adenocarcinoma | Unknown |
| E225K | Missense Mutation | Lung Adenocarcinoma | Unknown |
| L327M | Missense Mutation | Uterine Endometrioid Carcinoma | Unknown |
| G113W | Missense Mutation | Uterine Endometrioid Carcinoma | Unknown |
| E449V | Missense Mutation | Uterine Endometrioid Carcinoma | Unknown |
| A483T | Missense Mutation | Uterine Endometrioid Carcinoma | Unknown |
| L387M | Missense Mutation | Uterine Endometrioid Carcinoma | Unknown |
| M257V | Missense Mutation | Uterine Endometrioid Carcinoma | Unknown |
| R106W | Missense Mutation | Bladder Urothelial Carcinoma | Unknown |
| Q335* | Nonsense Mutation | Bladder Urothelial Carcinoma | Unknown |
| S356N | Missense Mutation | Renal Clear Cell Carcinoma | Unknown |
| G213R | Missense Mutation | Renal Clear Cell Carcinoma | Unknown |
| Q163E | Missense Mutation | Breast Invasive Ductal Carcinoma | Unknown |
| P217L | Missense Mutation | Cutaneous Melanoma | Unknown |
| P334S | Missense Mutation | Cutaneous Melanoma | Unknown |
| P217S | Missense Mutation | Cutaneous Melanoma | Unknown |
| S180F | Missense Mutation | Lentigo Maligna Melanoma | Unknown |
| P310Qfs*54 | Frame Shift Deletion | Hepatocellular Carcinoma | Unknown |
| P310Qfs*54 | Frame Shift Deletion | Stomach Adenocarcinoma | Unknown |
| T412Kfs*13 | Frame Shift Deletion | Stomach Adenocarcinoma | Unknown |
| G175Afs*4 | Frame Shift Deletion | Diffuse Type Stomach Adenocarcinoma | Unknown |
| A258Qfs*38 | Frame Shift Deletion | Lung Adenocarcinoma | Unknown |
| A258Qfs*38 | Frame Shift Deletion | Lung Adenocarcinoma | Unknown |
| L178F | Missense Mutation | Lung Adenocarcinoma | Unknown |
| X461_splice | Splice Site | Lung Adenocarcinoma | Unknown |
| S116F | Missense Mutation | Lung Squamous Cell Carcinoma | Unknown |
| E443G | Missense Mutation | Hepatocellular Carcinoma | Unknown |
| T507S | Missense Mutation | Hepatocellular Carcinoma | Unknown |
| R162K | Missense Mutation | Cutaneous Melanoma | Unknown |
| L316I | Missense Mutation | Uterine Carcinosarcoma/Uterine Malignant Mixed Mullerian Tumor | Unknown |
| I504? | Splice Region | Cutaneous Melanoma | Unknown |
| N503? | Splice Region | Uterine Endometrioid Carcinoma | Unknown |

| **FANCL Mutation** | **Mutation Type** | **Cancer Type** | **OncoKB Analysis** |
| --- | --- | --- | --- |
| L191Wfs*4 | Frame Shift Deletion | Diffuse Type Stomach Adenocarcinoma | Likely Oncogenic |
| X33_splice | Splice Site | Lung Squamous Cell Carcinoma | Likely Oncogenic |
| X32_splice | Splice Site | Lung Squamous Cell Carcinoma | Likely Oncogenic |
| X52_splice | Splice Site | Breast Invasive Ductal Carcinoma | Likely Oncogenic |
| X157_splice | Splice Site | Head and Neck Squamous Cell Carcinoma | Likely Oncogenic |
| Q71H | Missense Mutation | Breast Invasive Ductal Carcinoma | Unknown |
| R68Q | Missense Mutation | Uterine Endometrioid Carcinoma | Unknown |
| A51T | Missense Mutation | Stomach Adenocarcinoma | Unknown |
| H375R | Missense Mutation | Stomach Adenocarcinoma | Unknown |
| A192G | Missense Mutation | Stomach Adenocarcinoma | Unknown |
| L154S | Missense Mutation | Stomach Adenocarcinoma | Unknown |
| M305V | Missense Mutation | Head and Neck Squamous Cell Carcinoma | Unknown |
| L149V | Missense Mutation | Uterine Endometrioid Carcinoma | Unknown |
| Q99H | Missense Mutation | Uterine Endometrioid Carcinoma | Unknown |
| D35Y | Missense Mutation | Lung Squamous Cell Carcinoma | Unknown |
| D35Y | Missense Mutation | Lung Squamous Cell Carcinoma | Unknown |
| R98K | Missense Mutation | Lung Squamous Cell Carcinoma | Unknown |
| P107S | Missense Mutation | Lung Adenocarcinoma | Unknown |
| A143S | Missense Mutation | Lung Adenocarcinoma | Unknown |
| C310S | Missense Mutation | Lung Squamous Cell Carcinoma | Unknown |
| S196P | Missense Mutation | Lung Squamous Cell Carcinoma | Unknown |
| Q109H | Missense Mutation | Lung Adenocarcinoma | Unknown |
| E277V | Missense Mutation | Mucinous Stomach Adenocarcinoma | Unknown |
| F110S | Missense Mutation | Serous Ovarian Cancer | Unknown |
| P43S | Missense Mutation | Diffuse Large B-Cell Lymphoma | Unknown |
| P163L | Missense Mutation | Papillary Renal Cell Carcinoma | Unknown |
| I184T | Missense Mutation | Head and Neck Squamous Cell Carcinoma | Unknown |
| I309V | Missense Mutation | Head and Neck Squamous Cell Carcinoma | Unknown |
| Y339H | Missense Mutation | Uveal Melanoma | Unknown |
| T367I | Missense Mutation | Serous Ovarian Cancer | Unknown |
| W201L | Missense Mutation | Lung Squamous Cell Carcinoma | Unknown |

| **FANCM Mutation** | **Mutation Type** | **Cancer Type** | **OncoKB Analysis** |
| --- | --- | --- | --- |
| E735Lfs*26 | Frame Shift Insertion | Stomach Adenocarcinoma | Unknown |
| E735Lfs*26 | Frame Shift Insertion | Stomach Adenocarcinoma | Unknown |
| E1622del | In Frame Deletion | Breast Invasive Ductal Carcinoma | Unknown |
| D807* | Frame Shift Insertion | Breast Invasive Ductal Carcinoma | Unknown |
| K1567E | Missense Mutation | Glioblastoma Multiforme | Unknown |
| R6K | Missense Mutation | Glioblastoma Multiforme | Unknown |
| N344I | Missense Mutation | Glioblastoma Multiforme | Unknown |
| L1066V | Missense Mutation | Astrocytoma | Unknown |
| R569C | Missense Mutation | Cutaneous Melanoma | Unknown |
| R569C | Missense Mutation | Rectal Adenocarcinoma | Unknown |
| S734* | Frame Shift Deletion | Stomach Adenocarcinoma | Unknown |
| S734* | Frame Shift Deletion | Stomach Adenocarcinoma | Unknown |
| G535S | Missense Mutation | Acute Myeloid Leukemia | Unknown |
| V1336Sfs*8 | Frame Shift Insertion | Stomach Adenocarcinoma | Unknown |
| V1336Lfs*2 | Frame Shift Deletion | Stomach Adenocarcinoma | Unknown |
| V1336Lfs*2 | Frame Shift Deletion | Mucinous Stomach Adenocarcinoma | Unknown |
| V1336Lfs*2 | Frame Shift Deletion | Diffuse Type Stomach Adenocarcinoma | Unknown |
| V1336Lfs*2 | Frame Shift Deletion | Mucinous Stomach Adenocarcinoma | Unknown |
| V1336Lfs*2 | Frame Shift Deletion | Stomach Adenocarcinoma | Unknown |
| V1336Lfs*2 | Frame Shift Deletion | Stomach Adenocarcinoma | Unknown |
| V1336Lfs*2 | Frame Shift Deletion | Stomach Adenocarcinoma | Unknown |
| V1336Lfs*2 | Frame Shift Deletion | Esophageal Adenocarcinoma | Unknown |
| V1336Lfs*2 | Frame Shift Deletion | Head and Neck Squamous Cell Carcinoma | Unknown |
| A1942T | Missense Mutation | Stomach Adenocarcinoma | Unknown |
| E964K | Missense Mutation | Uterine Endometrioid Carcinoma | Unknown |
| E1622* | Nonsense Mutation | Lung Squamous Cell Carcinoma | Unknown |
| E1622* | Nonsense Mutation | Esophageal Adenocarcinoma | Unknown |
| E1622* | Nonsense Mutation | Lung Squamous Cell Carcinoma | Unknown |
| G1235V | Missense Mutation | Adrenocortical Carcinoma | Unknown |
| A44T | Missense Mutation | Stomach Adenocarcinoma | Unknown |
| D1146N | Missense Mutation | Stomach Adenocarcinoma | Unknown |
| K1639E | Missense Mutation | Stomach Adenocarcinoma | Unknown |
| P1096S | Missense Mutation | Stomach Adenocarcinoma | Unknown |
| R1456H | Missense Mutation | Stomach Adenocarcinoma | Unknown |
| L496R | Missense Mutation | Stomach Adenocarcinoma | Unknown |
| R756H | Missense Mutation | Stomach Adenocarcinoma | Unknown |
| S1064Vfs*31 | Frame Shift Deletion | Stomach Adenocarcinoma | Unknown |
| H644Wfs*4 | Frame Shift Deletion | Stomach Adenocarcinoma | Unknown |
| K285Q | Missense Mutation | Tubular Stomach Adenocarcinoma | Unknown |
| D54G | Missense Mutation | Stomach Adenocarcinoma | Unknown |
| M1566I | Missense Mutation | Stomach Adenocarcinoma | Unknown |
| N634D | Missense Mutation | Stomach Adenocarcinoma | Unknown |
| P743H | Missense Mutation | Diffuse Type Stomach Adenocarcinoma | Unknown |
| R658* | Nonsense Mutation | Uterine Endometrioid Carcinoma | Unknown |
| R658* | Nonsense Mutation | Colon Adenocarcinoma | Unknown |
| R185T | Missense Mutation | Head and Neck Squamous Cell Carcinoma | Unknown |
| S832C | Missense Mutation | Head and Neck Squamous Cell Carcinoma | Unknown |
| R92P | Missense Mutation | Head and Neck Squamous Cell Carcinoma | Unknown |
| E51* | Nonsense Mutation | Lung Adenocarcinoma | Unknown |
| R1424Q | Missense Mutation | Lung Adenocarcinoma | Unknown |
| G21V | Missense Mutation | Lung Adenocarcinoma | Unknown |
| T746S | Missense Mutation | Lung Adenocarcinoma | Unknown |
| R573* | Nonsense Mutation | Lung Adenocarcinoma | Unknown |
| D55E | Missense Mutation | Lung Adenocarcinoma | Unknown |
| S1005* | Nonsense Mutation | Lung Adenocarcinoma | Unknown |
| E1319* | Nonsense Mutation | Lung Adenocarcinoma | Unknown |
| D881G | Missense Mutation | Lung Adenocarcinoma | Unknown |
| L733V | Missense Mutation | Lung Adenocarcinoma | Unknown |
| V59F | Missense Mutation | Lung Adenocarcinoma | Unknown |
| R971K | Missense Mutation | Lung Adenocarcinoma | Unknown |
| R971K | Missense Mutation | Lung Adenocarcinoma | Unknown |
| D696Tfs*8 | Frame Shift Deletion | Lung Adenocarcinoma | Unknown |
| D696Tfs*8 | Frame Shift Deletion | Lung Adenocarcinoma | Unknown |
| R1204L | Missense Mutation | Lung Adenocarcinoma | Unknown |
| R1204L | Missense Mutation | Lung Adenocarcinoma | Unknown |
| I2022M | Missense Mutation | Lung Adenocarcinoma | Unknown |
| I2022M | Missense Mutation | Lung Adenocarcinoma | Unknown |
| T746I | Missense Mutation | Lung Adenocarcinoma | Unknown |
| T746I | Missense Mutation | Lung Adenocarcinoma | Unknown |
| S792* | Nonsense Mutation | Lung Adenocarcinoma | Unknown |
| S792* | Nonsense Mutation | Lung Adenocarcinoma | Unknown |
| C1624F | Missense Mutation | Lung Adenocarcinoma | Unknown |
| C1624F | Missense Mutation | Lung Adenocarcinoma | Unknown |
| M2000I | Missense Mutation | Lung Adenocarcinoma | Unknown |
| M2000I | Missense Mutation | Lung Adenocarcinoma | Unknown |
| Q531H | Missense Mutation | Lung Adenocarcinoma | Unknown |
| Q531H | Missense Mutation | Lung Adenocarcinoma | Unknown |
| R1207* | Nonsense Mutation | Lung Adenocarcinoma | Unknown |
| R1207* | Nonsense Mutation | Lung Adenocarcinoma | Unknown |
| P191H | Missense Mutation | Uterine Endometrioid Carcinoma | Unknown |
| E527D | Missense Mutation | Uterine Endometrioid Carcinoma | Unknown |
| Q1047H | Missense Mutation | Uterine Endometrioid Carcinoma | Unknown |
| E1651* | Nonsense Mutation | Uterine Endometrioid Carcinoma | Unknown |
| K1955N | Missense Mutation | Uterine Endometrioid Carcinoma | Unknown |
| Y85C | Missense Mutation | Uterine Endometrioid Carcinoma | Unknown |
| C69R | Missense Mutation | Uterine Endometrioid Carcinoma | Unknown |
| N871D | Missense Mutation | Uterine Endometrioid Carcinoma | Unknown |
| D1032Y | Missense Mutation | Uterine Endometrioid Carcinoma | Unknown |
| R1196H | Missense Mutation | Uterine Endometrioid Carcinoma | Unknown |
| R1860H | Missense Mutation | Uterine Endometrioid Carcinoma | Unknown |
| R1914K | Missense Mutation | Uterine Endometrioid Carcinoma | Unknown |
| C1478Y | Missense Mutation | Uterine Endometrioid Carcinoma | Unknown |
| S2009Y | Missense Mutation | Uterine Endometrioid Carcinoma | Unknown |
| E1832* | Nonsense Mutation | Uterine Endometrioid Carcinoma | Unknown |
| R624I | Missense Mutation | Uterine Endometrioid Carcinoma | Unknown |
| P32L | Missense Mutation | Uterine Endometrioid Carcinoma | Unknown |
| A1166V | Missense Mutation | Uterine Endometrioid Carcinoma | Unknown |
| N127S | Missense Mutation | Uterine Endometrioid Carcinoma | Unknown |
| S2004L | Missense Mutation | Uterine Endometrioid Carcinoma | Unknown |
| P1778L | Missense Mutation | Uterine Endometrioid Carcinoma | Unknown |
| S1053* | Nonsense Mutation | Uterine Endometrioid Carcinoma | Unknown |
| R1030Q | Missense Mutation | Uterine Endometrioid Carcinoma | Unknown |
| H1217N | Missense Mutation | Uterine Endometrioid Carcinoma | Unknown |
| K1287T | Missense Mutation | Uterine Endometrioid Carcinoma | Unknown |
| C1772* | Frame Shift Deletion | Uterine Endometrioid Carcinoma | Unknown |
| I1834M | Missense Mutation | Uterine Endometrioid Carcinoma | Unknown |
| P1726A | Missense Mutation | Bladder Urothelial Carcinoma | Unknown |
| N401Mfs*10 | Frame Shift Deletion | Bladder Urothelial Carcinoma | Unknown |
| W12* | Nonsense Mutation | Bladder Urothelial Carcinoma | Unknown |
| S734C | Missense Mutation | Bladder Urothelial Carcinoma | Unknown |
| E1971K | Missense Mutation | Bladder Urothelial Carcinoma | Unknown |
| V1625F | Missense Mutation | Renal Clear Cell Carcinoma | Unknown |
| L674P | Missense Mutation | Renal Clear Cell Carcinoma | Unknown |
| E1826K | Missense Mutation | Glioblastoma Multiforme | Unknown |
| M2000I | Missense Mutation | Lung Squamous Cell Carcinoma | Unknown |
| M2000I | Missense Mutation | Lung Squamous Cell Carcinoma | Unknown |
| I608M | Missense Mutation | Lung Squamous Cell Carcinoma | Unknown |
| I608M | Missense Mutation | Lung Squamous Cell Carcinoma | Unknown |
| V123L | Missense Mutation | Lung Squamous Cell Carcinoma | Unknown |
| V123L | Missense Mutation | Lung Squamous Cell Carcinoma | Unknown |
| E1952K | Missense Mutation | Lung Squamous Cell Carcinoma | Unknown |
| E1952K | Missense Mutation | Lung Squamous Cell Carcinoma | Unknown |
| Q1101E | Missense Mutation | Lung Squamous Cell Carcinoma | Unknown |
| Q1101E | Missense Mutation | Lung Squamous Cell Carcinoma | Unknown |
| E1610K | Missense Mutation | Lung Squamous Cell Carcinoma | Unknown |
| E1610K | Missense Mutation | Lung Squamous Cell Carcinoma | Unknown |
| X395_splice | Splice Site | Lung Squamous Cell Carcinoma | Unknown |
| X395_splice | Splice Site | Lung Squamous Cell Carcinoma | Unknown |
| H1103N | Missense Mutation | Lung Squamous Cell Carcinoma | Unknown |
| Q748E | Missense Mutation | Papillary Renal Cell Carcinoma | Unknown |
| D1702Y | Missense Mutation | Pancreatic Adenocarcinoma | Unknown |
| Q354K | Missense Mutation | Lung Squamous Cell Carcinoma | Unknown |
| E207* | Nonsense Mutation | Lung Squamous Cell Carcinoma | Unknown |
| R593P | Missense Mutation | Lung Adenocarcinoma | Unknown |
| Q498L | Missense Mutation | Lung Squamous Cell Carcinoma | Unknown |
| T1249A | Missense Mutation | Lung Adenocarcinoma | Unknown |
| D1672N | Missense Mutation | Lung Adenocarcinoma | Unknown |
| N319I | Missense Mutation | Lung Adenocarcinoma | Unknown |
| X596_splice | Splice Site | Lung Adenocarcinoma | Unknown |
| X721_splice | Splice Site | Lung Adenocarcinoma | Unknown |
| X721_splice | Splice Site | Lung Adenocarcinoma | Unknown |
| S488C | Missense Mutation | Lung Adenocarcinoma | Unknown |
| Q10* | Nonsense Mutation | Lung Adenocarcinoma | Unknown |
| L1182H | Missense Mutation | Lung Adenocarcinoma | Unknown |
| X596_splice | Splice Site | Lung Adenocarcinoma | Unknown |
| N1071Y | Missense Mutation | Lung Adenocarcinoma | Unknown |
| F1458C | Missense Mutation | Lung Adenocarcinoma | Unknown |
| D974H | Missense Mutation | Lung Squamous Cell Carcinoma | Unknown |
| E1533* | Nonsense Mutation | Lung Squamous Cell Carcinoma | Unknown |
| E417Q | Missense Mutation | Lung Squamous Cell Carcinoma | Unknown |
| D197H | Missense Mutation | Lung Squamous Cell Carcinoma | Unknown |
| L570V | Missense Mutation | Lung Squamous Cell Carcinoma | Unknown |
| G1261* | Nonsense Mutation | Lung Adenocarcinoma | Unknown |
| E1472Q | Missense Mutation | Lung Adenocarcinoma | Unknown |
| R18* | Nonsense Mutation | Lung Squamous Cell Carcinoma | Unknown |
| E869* | Nonsense Mutation | Lung Adenocarcinoma | Unknown |
| S1889R | Missense Mutation | Lung Squamous Cell Carcinoma | Unknown |
| K518E | Missense Mutation | Lung Adenocarcinoma | Unknown |
| Y445D | Missense Mutation | Lung Adenocarcinoma | Unknown |
| H1404Y | Missense Mutation | Lung Adenocarcinoma | Unknown |
| Q49H | Missense Mutation | Lung Adenocarcinoma | Unknown |
| L1435F | Missense Mutation | Lung Squamous Cell Carcinoma | Unknown |
| *2049Qext*20 | Nonstop Mutation | Lung Squamous Cell Carcinoma | Unknown |
| R100L | Missense Mutation | Lung Adenocarcinoma | Unknown |
| E1535Q | Missense Mutation | Lung Adenocarcinoma | Unknown |
| M1561I | Missense Mutation | Lung Adenocarcinoma | Unknown |
| Q1206* | Nonsense Mutation | Cutaneous Melanoma | Unknown |
| Q1206* | Nonsense Mutation | Esophageal Adenocarcinoma | Unknown |
| S1448F | Missense Mutation | Breast Invasive Lobular Carcinoma | Unknown |
| Y609Ifs*2 | Frame Shift Insertion | Metaplastic Breast Cancer | Unknown |
| E1677del | In Frame Deletion | Breast Invasive Ductal Carcinoma | Unknown |
| M1561T | Missense Mutation | Papillary Thyroid Cancer | Unknown |
| N1317Mfs*21 | Frame Shift Deletion | Hepatocellular Carcinoma | Unknown |
| E723K | Missense Mutation | Esophageal Adenocarcinoma | Unknown |
| R1838I | Missense Mutation | Esophageal Adenocarcinoma | Unknown |
| Q500* | Nonsense Mutation | Esophageal Adenocarcinoma | Unknown |
| M2000L | Missense Mutation | Esophageal Adenocarcinoma | Unknown |
| H812D | Missense Mutation | Esophageal Squamous Cell Carcinoma | Unknown |
| L1944F | Missense Mutation | Esophageal Squamous Cell Carcinoma | Unknown |
| R1562I | Missense Mutation | Esophageal Adenocarcinoma | Unknown |
| Y383H | Missense Mutation | Esophageal Squamous Cell Carcinoma | Unknown |
| D677V | Missense Mutation | Prostate Adenocarcinoma | Unknown |
| H1309L | Missense Mutation | Diffuse Type Stomach Adenocarcinoma | Unknown |
| E1421K | Missense Mutation | Stomach Adenocarcinoma | Unknown |
| F1763C | Missense Mutation | Diffuse Type Stomach Adenocarcinoma | Unknown |
| A1793T | Missense Mutation | Mucinous Stomach Adenocarcinoma | Unknown |
| N1317Kfs*2 | Frame Shift Insertion | Diffuse Type Stomach Adenocarcinoma | Unknown |
| R581P | Missense Mutation | Leiomyosarcoma | Unknown |
| Q1868E | Missense Mutation | Uterine Carcinosarcoma/Uterine Malignant Mixed Mullerian Tumor | Unknown |
| M768I | Missense Mutation | Uterine Carcinosarcoma/Uterine Malignant Mixed Mullerian Tumor | Unknown |
| Q600K | Missense Mutation | Uterine Carcinosarcoma/Uterine Malignant Mixed Mullerian Tumor | Unknown |
| I1296del | In Frame Deletion | Mucinous Adenocarcinoma of the Colon and Rectum | Unknown |
| D391N | Missense Mutation | Colon Adenocarcinoma | Unknown |
| S1127C | Missense Mutation | Colon Adenocarcinoma | Unknown |
| P43L | Missense Mutation | Mucinous Adenocarcinoma of the Colon and Rectum | Unknown |
| V1623G | Missense Mutation | Mucinous Adenocarcinoma of the Colon and Rectum | Unknown |
| I502F | Missense Mutation | Mucinous Adenocarcinoma of the Colon and Rectum | Unknown |
| S1276L | Missense Mutation | Mucinous Adenocarcinoma of the Colon and Rectum | Unknown |
| K854N | Missense Mutation | Colon Adenocarcinoma | Unknown |
| N1989H | Missense Mutation | Colon Adenocarcinoma | Unknown |
| N1473I | Missense Mutation | Colon Adenocarcinoma | Unknown |
| E268* | Nonsense Mutation | Rectal Adenocarcinoma | Unknown |
| F794C | Missense Mutation | Rectal Adenocarcinoma | Unknown |
| E702D | Missense Mutation | Rectal Adenocarcinoma | Unknown |
| N1058H | Missense Mutation | Rectal Adenocarcinoma | Unknown |
| R1666I | Missense Mutation | Serous Ovarian Cancer | Unknown |
| L1159V | Missense Mutation | Anaplastic Astrocytoma | Unknown |
| V1961F | Missense Mutation | Uveal Melanoma | Unknown |
| E1814V | Missense Mutation | Non-Seminomatous Germ Cell Tumor | Unknown |
| A254S | Missense Mutation | Seminoma | Unknown |
| L674I | Missense Mutation | Adrenocortical Carcinoma | Unknown |
| L211I | Missense Mutation | Breast Invasive Ductal Carcinoma | Unknown |
| S1808Y | Missense Mutation | Breast Invasive Ductal Carcinoma | Unknown |
| K462* | Nonsense Mutation | Diffuse Large B-Cell Lymphoma | Unknown |
| P1722L | Missense Mutation | Head and Neck Squamous Cell Carcinoma | Unknown |
| P1722L | Missense Mutation | Head and Neck Squamous Cell Carcinoma | Unknown |
| C921F | Missense Mutation | Head and Neck Squamous Cell Carcinoma | Unknown |
| E1685Q | Missense Mutation | Head and Neck Squamous Cell Carcinoma | Unknown |
| S944C | Missense Mutation | Head and Neck Squamous Cell Carcinoma | Unknown |
| S590F | Missense Mutation | Head and Neck Squamous Cell Carcinoma | Unknown |
| S484L | Missense Mutation | Head and Neck Squamous Cell Carcinoma | Unknown |
| R1456Afs*13 | Frame Shift Insertion | Hepatocellular Carcinoma | Unknown |
| R1810I | Missense Mutation | Hepatocellular Carcinoma | Unknown |
| N1752S | Missense Mutation | Hepatocellular Carcinoma | Unknown |
| R1457Dfs*4 | Frame Shift Deletion | Hepatocellular Carcinoma | Unknown |
| G1616S | Missense Mutation | Hepatocellular Carcinoma | Unknown |
| V1718M | Missense Mutation | Hepatocellular Carcinoma | Unknown |
| N1681I | Missense Mutation | Hepatocellular Carcinoma | Unknown |
| E1018V | Missense Mutation | Hepatocellular Carcinoma | Unknown |
| A122V | Missense Mutation | Hepatocellular Carcinoma plus Intrahepatic Cholangiocarcinoma | Unknown |
| L278F | Missense Mutation | Hepatocellular Carcinoma | Unknown |
| V618L | Missense Mutation | Pancreatic Adenocarcinoma | Unknown |
| R1099C | Missense Mutation | Pancreatic Adenocarcinoma | Unknown |
| A1516T | Missense Mutation | Papillary Renal Cell Carcinoma | Unknown |
| Y1918Mfs*5 | Frame Shift Deletion | Papillary Renal Cell Carcinoma | Unknown |
| E527G | Missense Mutation | Papillary Renal Cell Carcinoma | Unknown |
| C1065W | Missense Mutation | Papillary Renal Cell Carcinoma | Unknown |
| A1452T | Missense Mutation | Papillary Renal Cell Carcinoma | Unknown |
| S954F | Missense Mutation | Cutaneous Melanoma | Unknown |
| S884F | Missense Mutation | Cutaneous Melanoma | Unknown |
| D1205E | Missense Mutation | Cutaneous Melanoma | Unknown |
| R533C | Missense Mutation | Uterine Carcinosarcoma/Uterine Malignant Mixed Mullerian Tumor | Unknown |
| R533C | Missense Mutation | Cutaneous Melanoma | Unknown |
| R533C | Missense Mutation | Cutaneous Melanoma | Unknown |
| G583D | Missense Mutation | Cutaneous Melanoma | Unknown |
| P448S | Missense Mutation | Cutaneous Melanoma | Unknown |
| R313C | Missense Mutation | Cutaneous Melanoma | Unknown |
| D251Y | Missense Mutation | Cutaneous Melanoma | Unknown |
| S664F | Missense Mutation | Cutaneous Melanoma | Unknown |
| T87A | Missense Mutation | Cutaneous Melanoma | Unknown |
| R1865K | Missense Mutation | Cutaneous Melanoma | Unknown |
| R1225K | Missense Mutation | Cutaneous Melanoma | Unknown |
| Q1193* | Nonsense Mutation | Cutaneous Melanoma | Unknown |
| H872Y | Missense Mutation | Cutaneous Melanoma | Unknown |
| E476* | Nonsense Mutation | Cutaneous Melanoma | Unknown |
| E455K | Missense Mutation | Uterine Carcinosarcoma/Uterine Malignant Mixed Mullerian Tumor | Unknown |
| G873S | Missense Mutation | Cutaneous Melanoma | Unknown |
| G873A | Missense Mutation | Cutaneous Melanoma | Unknown |
| T848I | Missense Mutation | Tubular Stomach Adenocarcinoma | Unknown |
| F1484L | Missense Mutation | Hepatocellular Carcinoma | Unknown |
| R573L | Missense Mutation | Hepatocellular Carcinoma | Unknown |
| H844R | Missense Mutation | Hepatocellular Carcinoma | Unknown |
| T900I | Missense Mutation | Hepatocellular Carcinoma | Unknown |
| L1546* | Frame Shift Deletion | Hepatocellular Carcinoma | Unknown |
| Q1779P | Missense Mutation | Pancreatic Adenocarcinoma | Unknown |
| G170= | Splice Region | Seminoma | Unknown |
| P1124T | Missense Mutation | Stomach Adenocarcinoma | Unknown |
| A40E | Missense Mutation | Prostate Adenocarcinoma | Unknown |
| R1865M | Missense Mutation | Lung Adenocarcinoma | Unknown |
| V193F | Missense Mutation | Lung Adenocarcinoma | Unknown |
| F1328Cfs*6 | Frame Shift Deletion | Lung Squamous Cell Carcinoma | Unknown |

| **PALB2 Mutation** | **Mutation Type** | **Cancer Type** | **OncoKB Analysis** |
| --- | --- | --- | --- |
| P1009Lfs*6 | Frame Shift Deletion | Stomach Adenocarcinoma | Likely Oncogenic |
| Q370* | Nonsense Mutation | Serous Ovarian Cancer | Likely Oncogenic |
| M296* | Frame Shift Deletion | Stomach Adenocarcinoma | Likely Oncogenic |
| M296* | Frame Shift Deletion | Stomach Adenocarcinoma | Likely Oncogenic |
| M296* | Frame Shift Deletion | Tubular Stomach Adenocarcinoma | Likely Oncogenic |
| M296* | Frame Shift Deletion | Stomach Adenocarcinoma | Likely Oncogenic |
| N280Tfs*8 | Frame Shift Deletion | Stomach Adenocarcinoma | Likely Oncogenic |
| N280Tfs*8 | Frame Shift Deletion | Stomach Adenocarcinoma | Likely Oncogenic |
| M875Cfs*14 | Frame Shift Deletion | Lung Adenocarcinoma | Likely Oncogenic |
| M875Cfs*14 | Frame Shift Deletion | Lung Adenocarcinoma | Likely Oncogenic |
| E658* | Nonsense Mutation | Lung Squamous Cell Carcinoma | Likely Oncogenic |
| X1067_splice | Splice Site | Lung Squamous Cell Carcinoma | Likely Oncogenic |
| E884* | Nonsense Mutation | Lung Squamous Cell Carcinoma | Likely Oncogenic |
| K140Nfs*37 | Frame Shift Deletion | Lung Squamous Cell Carcinoma | Likely Oncogenic |
| Q1146* | Nonsense Mutation | Breast Invasive Ductal Carcinoma | Likely Oncogenic |
| E860* | Nonsense Mutation | Stomach Adenocarcinoma | Likely Oncogenic |
| W1140* | Nonsense Mutation | Mucinous Stomach Adenocarcinoma | Likely Oncogenic |
| E1010* | Frame Shift Insertion | Colon Adenocarcinoma | Likely Oncogenic |
| E181* | Nonsense Mutation | Rectal Adenocarcinoma | Likely Oncogenic |
| E263* | Nonsense Mutation | Serous Ovarian Cancer | Likely Oncogenic |
| D618Tfs*3 | Frame Shift Deletion | Papillary Renal Cell Carcinoma | Likely Oncogenic |
| Q1056* | Nonsense Mutation | Cutaneous Melanoma | Likely Oncogenic |
| L939W | Missense Mutation | Diffuse Large B-Cell Lymphoma | Likely Oncogenic |
| R753Q | Missense Mutation | Rectal Adenocarcinoma | Unknown |
| I1013V | Missense Mutation | Prostate Adenocarcinoma | Unknown |
| E837K | Missense Mutation | Prostate Adenocarcinoma | Unknown |
| T733A | Missense Mutation | Stomach Adenocarcinoma | Unknown |
| S1165L | Missense Mutation | Stomach Adenocarcinoma | Unknown |
| D586N | Missense Mutation | Head and Neck Squamous Cell Carcinoma | Unknown |
| N444S | Missense Mutation | Head and Neck Squamous Cell Carcinoma | Unknown |
| S489F | Missense Mutation | Head and Neck Squamous Cell Carcinoma | Unknown |
| C828F | Missense Mutation | Head and Neck Squamous Cell Carcinoma | Unknown |
| D986Y | Missense Mutation | Lung Adenocarcinoma | Unknown |
| D986Y | Missense Mutation | Lung Adenocarcinoma | Unknown |
| N340H | Missense Mutation | Lung Adenocarcinoma | Unknown |
| N340H | Missense Mutation | Lung Adenocarcinoma | Unknown |
| M1032I | Missense Mutation | Lung Adenocarcinoma | Unknown |
| M1032I | Missense Mutation | Lung Adenocarcinoma | Unknown |
| N851H | Missense Mutation | Uterine Endometrioid Carcinoma | Unknown |
| M1067T | Missense Mutation | Uterine Endometrioid Carcinoma | Unknown |
| A291V | Missense Mutation | Uterine Endometrioid Carcinoma | Unknown |
| A1173S | Missense Mutation | Uterine Endometrioid Carcinoma | Unknown |
| D1122N | Missense Mutation | Uterine Endometrioid Carcinoma | Unknown |
| A880V | Missense Mutation | Uterine Endometrioid Carcinoma | Unknown |
| T333A | Missense Mutation | Uterine Serous Carcinoma/Uterine Papillary Serous Carcinoma | Unknown |
| V546A | Missense Mutation | Uterine Endometrioid Carcinoma | Unknown |
| P529L | Missense Mutation | Uterine Serous Carcinoma/Uterine Papillary Serous Carcinoma | Unknown |
| E456Q | Missense Mutation | Bladder Urothelial Carcinoma | Unknown |
| V836I | Missense Mutation | Bladder Urothelial Carcinoma | Unknown |
| C949S | Missense Mutation | Renal Clear Cell Carcinoma | Unknown |
| S190L | Missense Mutation | Renal Clear Cell Carcinoma | Unknown |
| L607V | Missense Mutation | Lung Squamous Cell Carcinoma | Unknown |
| L607V | Missense Mutation | Lung Squamous Cell Carcinoma | Unknown |
| H1076D | Missense Mutation | Lung Squamous Cell Carcinoma | Unknown |
| H1076D | Missense Mutation | Lung Squamous Cell Carcinoma | Unknown |
| G780S | Missense Mutation | Lung Squamous Cell Carcinoma | Unknown |
| G780S | Missense Mutation | Lung Squamous Cell Carcinoma | Unknown |
| D1156H | Missense Mutation | Lung Squamous Cell Carcinoma | Unknown |
| D1156H | Missense Mutation | Lung Squamous Cell Carcinoma | Unknown |
| H1184N | Missense Mutation | Lung Squamous Cell Carcinoma | Unknown |
| E820Q | Missense Mutation | Lung Squamous Cell Carcinoma | Unknown |
| C653F | Missense Mutation | Lung Adenocarcinoma | Unknown |
| D2G | Missense Mutation | Lung Adenocarcinoma | Unknown |
| E669K | Missense Mutation | Lung Adenocarcinoma | Unknown |
| A846S | Missense Mutation | Lung Adenocarcinoma | Unknown |
| E943K | Missense Mutation | Lung Adenocarcinoma | Unknown |
| K662E | Missense Mutation | Lung Adenocarcinoma | Unknown |
| C1147F | Missense Mutation | Lung Squamous Cell Carcinoma | Unknown |
| Q602L | Missense Mutation | Lung Adenocarcinoma | Unknown |
| H261R | Missense Mutation | Lung Squamous Cell Carcinoma | Unknown |
| S133I | Missense Mutation | Lung Adenocarcinoma | Unknown |
| H1170L | Missense Mutation | Lung Squamous Cell Carcinoma | Unknown |
| L901M | Missense Mutation | Lung Squamous Cell Carcinoma | Unknown |
| G115E | Missense Mutation | Breast Invasive Lobular Carcinoma | Unknown |
| D234N | Missense Mutation | Breast Invasive Ductal Carcinoma | Unknown |
| G971R | Missense Mutation | Breast Invasive Ductal Carcinoma | Unknown |
| Q506K | Missense Mutation | Esophageal Adenocarcinoma | Unknown |
| A712V | Missense Mutation | Prostate Adenocarcinoma | Unknown |
| H593Y | Missense Mutation | Myxofibrosarcoma | Unknown |
| E180K | Missense Mutation | Leiomyosarcoma | Unknown |
| P687S | Missense Mutation | Mucinous Adenocarcinoma of the Colon and Rectum | Unknown |
| P1112L | Missense Mutation | Colon Adenocarcinoma | Unknown |
| V805F | Missense Mutation | Mucinous Adenocarcinoma of the Colon and Rectum | Unknown |
| D952G | Missense Mutation | Mucinous Adenocarcinoma of the Colon and Rectum | Unknown |
| E19D | Missense Mutation | Rectal Adenocarcinoma | Unknown |
| K486E | Missense Mutation | Serous Ovarian Cancer | Unknown |
| R516I | Missense Mutation | Serous Ovarian Cancer | Unknown |
| E53K | Missense Mutation | Breast Invasive Ductal Carcinoma | Unknown |
| S201N | Missense Mutation | Breast Invasive Ductal Carcinoma | Unknown |
| G1145S | Missense Mutation | Diffuse Large B-Cell Lymphoma | Unknown |
| E384K | Missense Mutation | Head and Neck Squamous Cell Carcinoma | Unknown |
| G1043C | Missense Mutation | Head and Neck Squamous Cell Carcinoma | Unknown |
| D595A | Missense Mutation | Pancreatic Adenocarcinoma | Unknown |
| A308T | Missense Mutation | Pancreatic Adenocarcinoma | Unknown |
| S500F | Missense Mutation | Papillary Renal Cell Carcinoma | Unknown |
| T413R | Missense Mutation | Papillary Renal Cell Carcinoma | Unknown |
| V487D | Missense Mutation | Papillary Renal Cell Carcinoma | Unknown |
| S835P | Missense Mutation | Cutaneous Melanoma | Unknown |
| E892K | Missense Mutation | Cutaneous Melanoma | Unknown |
| S951F | Missense Mutation | Cutaneous Melanoma | Unknown |
| P707S | Missense Mutation | Cutaneous Melanoma | Unknown |
| P1077S | Missense Mutation | Cutaneous Melanoma | Unknown |
| V917D | Missense Mutation | Cutaneous Melanoma | Unknown |
| S488I | Missense Mutation | Invasive Breast Carcinoma | Unknown |
| P845S | Missense Mutation | Lentigo Maligna Melanoma | Unknown |
| P812S | Missense Mutation | Desmoplastic Melanoma | Unknown |
| E570G | Missense Mutation | Hepatocellular Carcinoma | Unknown |
| R718S | Missense Mutation | Non-Seminomatous Germ Cell Tumor | Unknown |
| M1007K | Missense Mutation | Head and Neck Squamous Cell Carcinoma | Unknown |
| E94Q | Missense Mutation | Lung Adenocarcinoma | Unknown |
| T235K | Missense Mutation | Lung Adenocarcinoma | Unknown |

| **POLQ Mutation** | **Mutation Type** | **Cancer Type** | **OncoKB Analysis** |
| --- | --- | --- | --- |
| L1430Ffs*5 | Frame Shift Insertion | Tubular Stomach Adenocarcinoma | Unknown |
| L1430Ffs*5 | Frame Shift Insertion | Prostate Adenocarcinoma | Unknown |
| L1430Ffs*5 | Frame Shift Insertion | Cutaneous Melanoma | Unknown |
| L1430Ffs*5 | Frame Shift Insertion | Lung Squamous Cell Carcinoma | Unknown |
| H2539Y | Missense Mutation | Head and Neck Squamous Cell Carcinoma | Unknown |
| *2591Sext*4 | Nonstop Mutation | Breast Invasive Ductal Carcinoma | Unknown |
| L1490P | Missense Mutation | Breast Invasive Ductal Carcinoma | Unknown |
| G2445V | Missense Mutation | Breast Invasive Ductal Carcinoma | Unknown |
| P1588L | Missense Mutation | Undifferentiated Pleomorphic Sarcoma/Malignant Fibrous Histiocytoma/High-Grade Spindle Cell Sarcoma | Unknown |
| P1588L | Missense Mutation | Breast Invasive Ductal Carcinoma | Unknown |
| S448Y | Missense Mutation | Breast Invasive Ductal Carcinoma | Unknown |
| Q578* | Nonsense Mutation | Bladder Urothelial Carcinoma | Unknown |
| Q578* | Nonsense Mutation | Breast Invasive Ductal Carcinoma | Unknown |
| R2447H | Missense Mutation | Oligoastrocytoma | Unknown |
| R1044C | Missense Mutation | Lung Adenocarcinoma | Unknown |
| K1619T | Missense Mutation | Glioblastoma Multiforme | Unknown |
| R860Q | Missense Mutation | Uterine Endometrioid Carcinoma | Unknown |
| R860Q | Missense Mutation | Uterine Endometrioid Carcinoma | Unknown |
| R860Q | Missense Mutation | Colon Adenocarcinoma | Unknown |
| R860Q | Missense Mutation | Colon Adenocarcinoma | Unknown |
| V577A | Missense Mutation | Cutaneous Melanoma | Unknown |
| R1040* | Nonsense Mutation | Cutaneous Melanoma | Unknown |
| R1040* | Nonsense Mutation | Cutaneous Melanoma | Unknown |
| E369K | Missense Mutation | Prostate Adenocarcinoma | Unknown |
| Q1361* | Nonsense Mutation | Head and Neck Squamous Cell Carcinoma | Unknown |
| A600V | Missense Mutation | Stomach Adenocarcinoma | Unknown |
| T421Lfs*29 | Frame Shift Deletion | Stomach Adenocarcinoma | Unknown |
| R359* | Nonsense Mutation | Stomach Adenocarcinoma | Unknown |
| E82V | Missense Mutation | Stomach Adenocarcinoma | Unknown |
| P2145Qfs*5 | Frame Shift Deletion | Hepatocellular Carcinoma | Unknown |
| P2145Qfs*5 | Frame Shift Deletion | Stomach Adenocarcinoma | Unknown |
| L1430* | Frame Shift Deletion | Papillary Stomach Adenocarcinoma | Unknown |
| L1430* | Frame Shift Deletion | Stomach Adenocarcinoma | Unknown |
| L1430* | Frame Shift Deletion | Stomach Adenocarcinoma | Unknown |
| S306L | Missense Mutation | Stomach Adenocarcinoma | Unknown |
| R132W | Missense Mutation | Stomach Adenocarcinoma | Unknown |
| Q2065* | Nonsense Mutation | Stomach Adenocarcinoma | Unknown |
| K475M | Missense Mutation | Stomach Adenocarcinoma | Unknown |
| R745C | Missense Mutation | Tubular Stomach Adenocarcinoma | Unknown |
| R460Q | Missense Mutation | Tubular Stomach Adenocarcinoma | Unknown |
| S246F | Missense Mutation | Stomach Adenocarcinoma | Unknown |
| V274M | Missense Mutation | Stomach Adenocarcinoma | Unknown |
| A431P | Missense Mutation | Stomach Adenocarcinoma | Unknown |
| P1798H | Missense Mutation | Stomach Adenocarcinoma | Unknown |
| P2451S | Missense Mutation | Stomach Adenocarcinoma | Unknown |
| R1723H | Missense Mutation | Stomach Adenocarcinoma | Unknown |
| A1831T | Missense Mutation | Stomach Adenocarcinoma | Unknown |
| L1410M | Missense Mutation | Stomach Adenocarcinoma | Unknown |
| L2378P | Missense Mutation | Mucinous Stomach Adenocarcinoma | Unknown |
| M2280V | Missense Mutation | Mucinous Stomach Adenocarcinoma | Unknown |
| K640N | Missense Mutation | Stomach Adenocarcinoma | Unknown |
| E1983V | Missense Mutation | Head and Neck Squamous Cell Carcinoma | Unknown |
| C2099F | Missense Mutation | Head and Neck Squamous Cell Carcinoma | Unknown |
| P2003L | Missense Mutation | Hepatocellular Carcinoma | Unknown |
| P2003L | Missense Mutation | Head and Neck Squamous Cell Carcinoma | Unknown |
| S182C | Missense Mutation | Head and Neck Squamous Cell Carcinoma | Unknown |
| R2315* | Nonsense Mutation | Head and Neck Squamous Cell Carcinoma | Unknown |
| S176C | Missense Mutation | Head and Neck Squamous Cell Carcinoma | Unknown |
| Q709* | Nonsense Mutation | Head and Neck Squamous Cell Carcinoma | Unknown |
| L1291V | Missense Mutation | Head and Neck Squamous Cell Carcinoma | Unknown |
| S1060F | Missense Mutation | Head and Neck Squamous Cell Carcinoma | Unknown |
| Q2441E | Missense Mutation | Head and Neck Squamous Cell Carcinoma | Unknown |
| Q978R | Missense Mutation | Head and Neck Squamous Cell Carcinoma | Unknown |
| T241N | Missense Mutation | Lung Adenocarcinoma | Unknown |
| Q578H | Missense Mutation | Lung Adenocarcinoma | Unknown |
| Q578H | Missense Mutation | Lung Squamous Cell Carcinoma | Unknown |
| P269S | Missense Mutation | Lung Adenocarcinoma | Unknown |
| R843L | Missense Mutation | Lung Adenocarcinoma | Unknown |
| M1566I | Missense Mutation | Lung Adenocarcinoma | Unknown |
| R1044L | Missense Mutation | Lung Adenocarcinoma | Unknown |
| G1095V | Missense Mutation | Lung Adenocarcinoma | Unknown |
| E583D | Missense Mutation | Lung Adenocarcinoma | Unknown |
| W1631S | Missense Mutation | Lung Adenocarcinoma | Unknown |
| W1631S | Missense Mutation | Lung Adenocarcinoma | Unknown |
| D1997Y | Missense Mutation | Lung Adenocarcinoma | Unknown |
| D1997Y | Missense Mutation | Lung Adenocarcinoma | Unknown |
| E1283* | Nonsense Mutation | Lung Adenocarcinoma | Unknown |
| E1283* | Nonsense Mutation | Lung Adenocarcinoma | Unknown |
| D1543H | Missense Mutation | Lung Adenocarcinoma | Unknown |
| D1543H | Missense Mutation | Lung Adenocarcinoma | Unknown |
| L1077V | Missense Mutation | Lung Adenocarcinoma | Unknown |
| L1077V | Missense Mutation | Lung Adenocarcinoma | Unknown |
| S1036N | Missense Mutation | Lung Adenocarcinoma | Unknown |
| S1036N | Missense Mutation | Lung Adenocarcinoma | Unknown |
| M680I | Missense Mutation | Lung Adenocarcinoma | Unknown |
| M680I | Missense Mutation | Lung Adenocarcinoma | Unknown |
| C901F | Missense Mutation | Lung Adenocarcinoma | Unknown |
| C901F | Missense Mutation | Lung Adenocarcinoma | Unknown |
| N1287Y | Missense Mutation | Lung Adenocarcinoma | Unknown |
| N1287Y | Missense Mutation | Lung Adenocarcinoma | Unknown |
| Q1603E | Missense Mutation | Lung Adenocarcinoma | Unknown |
| Q1603E | Missense Mutation | Lung Adenocarcinoma | Unknown |
| D1988H | Missense Mutation | Lung Adenocarcinoma | Unknown |
| D1988H | Missense Mutation | Lung Adenocarcinoma | Unknown |
| Q2556* | Nonsense Mutation | Lung Adenocarcinoma | Unknown |
| Q2556* | Nonsense Mutation | Lung Adenocarcinoma | Unknown |
| G2281V | Missense Mutation | Lung Adenocarcinoma | Unknown |
| G2281V | Missense Mutation | Lung Adenocarcinoma | Unknown |
| R798P | Missense Mutation | Lung Adenocarcinoma | Unknown |
| R798P | Missense Mutation | Lung Adenocarcinoma | Unknown |
| E332* | Nonsense Mutation | Uterine Endometrioid Carcinoma | Unknown |
| E2300D | Missense Mutation | Uterine Endometrioid Carcinoma | Unknown |
| R467Q | Missense Mutation | Uterine Endometrioid Carcinoma | Unknown |
| R467Q | Missense Mutation | Lung Squamous Cell Carcinoma | Unknown |
| K109N | Missense Mutation | Uterine Endometrioid Carcinoma | Unknown |
| R2518Q | Missense Mutation | Uterine Endometrioid Carcinoma | Unknown |
| A2134T | Missense Mutation | Uterine Endometrioid Carcinoma | Unknown |
| D1053N | Missense Mutation | Uterine Endometrioid Carcinoma | Unknown |
| L2017I | Missense Mutation | Uterine Serous Carcinoma/Uterine Papillary Serous Carcinoma | Unknown |
| E691A | Missense Mutation | Uterine Endometrioid Carcinoma | Unknown |
| K1581N | Missense Mutation | Uterine Endometrioid Carcinoma | Unknown |
| K236N | Missense Mutation | Uterine Endometrioid Carcinoma | Unknown |
| E2549* | Nonsense Mutation | Uterine Endometrioid Carcinoma | Unknown |
| K167Q | Missense Mutation | Uterine Endometrioid Carcinoma | Unknown |
| A544G | Missense Mutation | Uterine Endometrioid Carcinoma | Unknown |
| N2353H | Missense Mutation | Uterine Endometrioid Carcinoma | Unknown |
| P1596A | Missense Mutation | Uterine Endometrioid Carcinoma | Unknown |
| P1479A | Missense Mutation | Uterine Endometrioid Carcinoma | Unknown |
| V1359I | Missense Mutation | Uterine Endometrioid Carcinoma | Unknown |
| E1530K | Missense Mutation | Uterine Endometrioid Carcinoma | Unknown |
| S1440L | Missense Mutation | Uterine Endometrioid Carcinoma | Unknown |
| L1423F | Missense Mutation | Uterine Endometrioid Carcinoma | Unknown |
| Q780H | Missense Mutation | Uterine Endometrioid Carcinoma | Unknown |
| Q780H | Missense Mutation | Lung Adenocarcinoma | Unknown |
| R226Q | Missense Mutation | Uterine Endometrioid Carcinoma | Unknown |
| R226Q | Missense Mutation | Cutaneous Melanoma | Unknown |
| E1680* | Nonsense Mutation | Uterine Endometrioid Carcinoma | Unknown |
| P2018T | Missense Mutation | Uterine Serous Carcinoma/Uterine Papillary Serous Carcinoma | Unknown |
| V80L | Missense Mutation | Uterine Endometrioid Carcinoma | Unknown |
| A2189V | Missense Mutation | Uterine Serous Carcinoma/Uterine Papillary Serous Carcinoma | Unknown |
| S1635* | Nonsense Mutation | Bladder Urothelial Carcinoma | Unknown |
| S1231* | Nonsense Mutation | Bladder Urothelial Carcinoma | Unknown |
| H2341Y | Missense Mutation | Bladder Urothelial Carcinoma | Unknown |
| I2258M | Missense Mutation | Bladder Urothelial Carcinoma | Unknown |
| E1474K | Missense Mutation | Bladder Urothelial Carcinoma | Unknown |
| S306* | Nonsense Mutation | Bladder Urothelial Carcinoma | Unknown |
| E917K | Missense Mutation | Bladder Urothelial Carcinoma | Unknown |
| R2241* | Nonsense Mutation | Renal Clear Cell Carcinoma | Unknown |
| Q2425K | Missense Mutation | Renal Clear Cell Carcinoma | Unknown |
| E1560V | Missense Mutation | Renal Clear Cell Carcinoma | Unknown |
| E1202A | Missense Mutation | Renal Clear Cell Carcinoma | Unknown |
| C1078S | Missense Mutation | Renal Clear Cell Carcinoma | Unknown |
| A534G | Missense Mutation | Glioblastoma Multiforme | Unknown |
| F944C | Missense Mutation | Glioblastoma Multiforme | Unknown |
| S967G | Missense Mutation | Lung Squamous Cell Carcinoma | Unknown |
| S967G | Missense Mutation | Lung Squamous Cell Carcinoma | Unknown |
| E685K | Missense Mutation | Lung Squamous Cell Carcinoma | Unknown |
| E685K | Missense Mutation | Lung Squamous Cell Carcinoma | Unknown |
| N2068D | Missense Mutation | Lung Squamous Cell Carcinoma | Unknown |
| N2068D | Missense Mutation | Lung Squamous Cell Carcinoma | Unknown |
| D1833E | Missense Mutation | Lung Squamous Cell Carcinoma | Unknown |
| D1833E | Missense Mutation | Lung Squamous Cell Carcinoma | Unknown |
| I1259T | Missense Mutation | Lung Squamous Cell Carcinoma | Unknown |
| I1259T | Missense Mutation | Lung Squamous Cell Carcinoma | Unknown |
| R2043L | Missense Mutation | Lung Squamous Cell Carcinoma | Unknown |
| R2043L | Missense Mutation | Lung Squamous Cell Carcinoma | Unknown |
| P1479S | Missense Mutation | Lung Squamous Cell Carcinoma | Unknown |
| P1479S | Missense Mutation | Lung Squamous Cell Carcinoma | Unknown |
| D602Y | Missense Mutation | Lung Squamous Cell Carcinoma | Unknown |
| D602Y | Missense Mutation | Lung Squamous Cell Carcinoma | Unknown |
| G524A | Missense Mutation | Lung Squamous Cell Carcinoma | Unknown |
| G524A | Missense Mutation | Lung Squamous Cell Carcinoma | Unknown |
| P1798S | Missense Mutation | Lung Squamous Cell Carcinoma | Unknown |
| P1798S | Missense Mutation | Lung Squamous Cell Carcinoma | Unknown |
| P1640Q | Missense Mutation | Lung Squamous Cell Carcinoma | Unknown |
| S2233L | Missense Mutation | Lung Squamous Cell Carcinoma | Unknown |
| S2233L | Missense Mutation | Lung Squamous Cell Carcinoma | Unknown |
| V1016F | Missense Mutation | Lung Squamous Cell Carcinoma | Unknown |
| V1016F | Missense Mutation | Lung Adenocarcinoma | Unknown |
| V1016F | Missense Mutation | Lung Squamous Cell Carcinoma | Unknown |
| S1628L | Missense Mutation | Lung Squamous Cell Carcinoma | Unknown |
| S1628L | Missense Mutation | Lung Squamous Cell Carcinoma | Unknown |
| M639L | Missense Mutation | Lung Squamous Cell Carcinoma | Unknown |
| M639L | Missense Mutation | Lung Squamous Cell Carcinoma | Unknown |
| Q1360K | Missense Mutation | Lung Squamous Cell Carcinoma | Unknown |
| R200H | Missense Mutation | Pancreatic Adenocarcinoma | Unknown |
| R200H | Missense Mutation | Mucinous Adenocarcinoma of the Colon and Rectum | Unknown |
| Q2467E | Missense Mutation | Lung Squamous Cell Carcinoma | Unknown |
| R1159G | Missense Mutation | Lung Squamous Cell Carcinoma | Unknown |
| Y758Rfs*36 | Frame Shift Deletion | Lung Squamous Cell Carcinoma | Unknown |
| R2518L | Missense Mutation | Lung Squamous Cell Carcinoma | Unknown |
| R1589G | Missense Mutation | Lung Squamous Cell Carcinoma | Unknown |
| R2505* | Nonsense Mutation | Lung Squamous Cell Carcinoma | Unknown |
| X537_splice | Splice Site | Lung Adenocarcinoma | Unknown |
| C189F | Missense Mutation | Lung Adenocarcinoma | Unknown |
| D1887E | Missense Mutation | Lung Adenocarcinoma | Unknown |
| K2066M | Missense Mutation | Lung Adenocarcinoma | Unknown |
| S1800I | Missense Mutation | Lung Adenocarcinoma | Unknown |
| S601I | Missense Mutation | Lung Adenocarcinoma | Unknown |
| V215L | Missense Mutation | Lung Adenocarcinoma | Unknown |
| P733L | Missense Mutation | Lung Squamous Cell Carcinoma | Unknown |
| G2524V | Missense Mutation | Lung Adenocarcinoma | Unknown |
| W1631L | Missense Mutation | Lung Adenocarcinoma | Unknown |
| Q2401R | Missense Mutation | Lung Squamous Cell Carcinoma | Unknown |
| E2088Q | Missense Mutation | Lung Squamous Cell Carcinoma | Unknown |
| V1478F | Missense Mutation | Lung Adenocarcinoma | Unknown |
| R2460L | Missense Mutation | Lung Squamous Cell Carcinoma | Unknown |
| T333K | Missense Mutation | Lung Adenocarcinoma | Unknown |
| D1531V | Missense Mutation | Lung Adenocarcinoma | Unknown |
| V38G | Missense Mutation | Lung Squamous Cell Carcinoma | Unknown |
| L446V | Missense Mutation | Lung Squamous Cell Carcinoma | Unknown |
| K2521E | Missense Mutation | Lung Squamous Cell Carcinoma | Unknown |
| R1849Q | Missense Mutation | Lung Adenocarcinoma | Unknown |
| D1497Y | Missense Mutation | Lung Adenocarcinoma | Unknown |
| A2364S | Missense Mutation | Lung Adenocarcinoma | Unknown |
| D660Y | Missense Mutation | Lung Adenocarcinoma | Unknown |
| Q2081* | Nonsense Mutation | Lung Squamous Cell Carcinoma | Unknown |
| I437M | Missense Mutation | Lung Squamous Cell Carcinoma | Unknown |
| Q805L | Missense Mutation | Lung Squamous Cell Carcinoma | Unknown |
| G1622W | Missense Mutation | Lung Adenocarcinoma | Unknown |
| P1777H | Missense Mutation | Lung Adenocarcinoma | Unknown |
| S1786Ifs*30 | Frame Shift Deletion | Lung Squamous Cell Carcinoma | Unknown |
| D286E | Missense Mutation | Lung Squamous Cell Carcinoma | Unknown |
| L440F | Missense Mutation | Lung Adenocarcinoma | Unknown |
| S925F | Missense Mutation | Lung Squamous Cell Carcinoma | Unknown |
| R1670* | Nonsense Mutation | Lung Squamous Cell Carcinoma | Unknown |
| L1128V | Missense Mutation | Lung Squamous Cell Carcinoma | Unknown |
| V1830L | Missense Mutation | Lung Squamous Cell Carcinoma | Unknown |
| S301F | Missense Mutation | Cutaneous Melanoma | Unknown |
| S1660L | Missense Mutation | Breast Invasive Ductal Carcinoma | Unknown |
| L329F | Missense Mutation | Breast Invasive Ductal Carcinoma | Unknown |
| T663S | Missense Mutation | Breast Invasive Ductal Carcinoma | Unknown |
| X1925_splice | Splice Site | Breast Invasive Ductal Carcinoma | Unknown |
| A2329V | Missense Mutation | Follicular Thyroid Cancer | Unknown |
| T1089P | Missense Mutation | Esophageal Adenocarcinoma | Unknown |
| A2356V | Missense Mutation | Esophageal Adenocarcinoma | Unknown |
| S502* | Nonsense Mutation | Esophageal Adenocarcinoma | Unknown |
| L536M | Missense Mutation | Esophageal Adenocarcinoma | Unknown |
| T604K | Missense Mutation | Esophageal Squamous Cell Carcinoma | Unknown |
| E526K | Missense Mutation | Esophageal Squamous Cell Carcinoma | Unknown |
| L1324V | Missense Mutation | Esophageal Squamous Cell Carcinoma | Unknown |
| S929T | Missense Mutation | Prostate Adenocarcinoma | Unknown |
| L1808S | Missense Mutation | Prostate Adenocarcinoma | Unknown |
| I2165N | Missense Mutation | Prostate Adenocarcinoma | Unknown |
| K933Nfs*2 | Frame Shift Deletion | Prostate Adenocarcinoma | Unknown |
| R1197Q | Missense Mutation | Mucinous Stomach Adenocarcinoma | Unknown |
| W771* | Nonsense Mutation | Mucinous Stomach Adenocarcinoma | Unknown |
| R396H | Missense Mutation | Mucinous Stomach Adenocarcinoma | Unknown |
| V188I | Missense Mutation | Mucinous Stomach Adenocarcinoma | Unknown |
| G173S | Missense Mutation | Mucinous Stomach Adenocarcinoma | Unknown |
| P1505Ifs*3 | Frame Shift Insertion | Stomach Adenocarcinoma | Unknown |
| K1025Rfs*4 | Frame Shift Deletion | Stomach Adenocarcinoma | Unknown |
| P1891L | Missense Mutation | Undifferentiated Pleomorphic Sarcoma/Malignant Fibrous Histiocytoma/High-Grade Spindle Cell Sarcoma | Unknown |
| D2408Y | Missense Mutation | Dedifferentiated Liposarcoma | Unknown |
| V1586L | Missense Mutation | Undifferentiated Pleomorphic Sarcoma/Malignant Fibrous Histiocytoma/High-Grade Spindle Cell Sarcoma | Unknown |
| P1588S | Missense Mutation | Undifferentiated Pleomorphic Sarcoma/Malignant Fibrous Histiocytoma/High-Grade Spindle Cell Sarcoma | Unknown |
| K151* | Nonsense Mutation | Uterine Carcinosarcoma/Uterine Malignant Mixed Mullerian Tumor | Unknown |
| R2466H | Missense Mutation | Colon Adenocarcinoma | Unknown |
| R2043* | Nonsense Mutation | Colon Adenocarcinoma | Unknown |
| G1256V | Missense Mutation | Colon Adenocarcinoma | Unknown |
| E1680K | Missense Mutation | Colon Adenocarcinoma | Unknown |
| S1786R | Missense Mutation | Colon Adenocarcinoma | Unknown |
| R533Q | Missense Mutation | Rectal Adenocarcinoma | Unknown |
| I1973L | Missense Mutation | Rectal Adenocarcinoma | Unknown |
| S1032L | Missense Mutation | Rectal Adenocarcinoma | Unknown |
| E386* | Nonsense Mutation | Rectal Adenocarcinoma | Unknown |
| P611A | Missense Mutation | Serous Ovarian Cancer | Unknown |
| P2322A | Missense Mutation | Serous Ovarian Cancer | Unknown |
| P2279S | Missense Mutation | Serous Ovarian Cancer | Unknown |
| A1062T | Missense Mutation | Thymoma | Unknown |
| G490= | Splice Region | Stomach Adenocarcinoma | Unknown |
| D2377Y | Missense Mutation | Adrenocortical Carcinoma | Unknown |
| E1480* | Nonsense Mutation | Breast Invasive Ductal Carcinoma | Unknown |
| Y1298* | Nonsense Mutation | Renal Clear Cell Carcinoma | Unknown |
| G788D | Missense Mutation | Diffuse Large B-Cell Lymphoma | Unknown |
| V1991M | Missense Mutation | Esophageal Adenocarcinoma | Unknown |
| S1697F | Missense Mutation | Head and Neck Squamous Cell Carcinoma | Unknown |
| S910L | Missense Mutation | Head and Neck Squamous Cell Carcinoma | Unknown |
| S1102C | Missense Mutation | Head and Neck Squamous Cell Carcinoma | Unknown |
| R1261K | Missense Mutation | Head and Neck Squamous Cell Carcinoma | Unknown |
| M1509I | Missense Mutation | Head and Neck Squamous Cell Carcinoma | Unknown |
| S1805L | Missense Mutation | Head and Neck Squamous Cell Carcinoma | Unknown |
| L1196V | Missense Mutation | Head and Neck Squamous Cell Carcinoma | Unknown |
| A842S | Missense Mutation | Head and Neck Squamous Cell Carcinoma | Unknown |
| X370_splice | Splice Site | Diffuse Type Stomach Adenocarcinoma | Unknown |
| X115_splice | Splice Site | Stomach Adenocarcinoma | Unknown |
| E2300V | Missense Mutation | Hepatocellular Carcinoma | Unknown |
| X211_splice | Splice Site | Hepatocellular Carcinoma | Unknown |
| V1146M | Missense Mutation | Hepatocellular Carcinoma | Unknown |
| R745S | Missense Mutation | Hepatocellular Carcinoma | Unknown |
| A2329S | Missense Mutation | Hepatocellular Carcinoma | Unknown |
| E314G | Missense Mutation | Hepatocellular Carcinoma | Unknown |
| F923L | Missense Mutation | Hepatocellular Carcinoma plus Intrahepatic Cholangiocarcinoma | Unknown |
| Y2420N | Missense Mutation | Hepatocellular Carcinoma | Unknown |
| K9I | Missense Mutation | Hepatocellular Carcinoma | Unknown |
| Q162R | Missense Mutation | Hepatocellular Carcinoma | Unknown |
| T2442Nfs*6 | Frame Shift Deletion | Hepatocellular Carcinoma | Unknown |
| G580R | Missense Mutation | Hepatocellular Carcinoma | Unknown |
| P1998L | Missense Mutation | Hepatocellular Carcinoma | Unknown |
| P1374L | Missense Mutation | Hepatocellular Carcinoma | Unknown |
| V213M | Missense Mutation | Pancreatic Adenocarcinoma | Unknown |
| L128F | Missense Mutation | Papillary Renal Cell Carcinoma | Unknown |
| Y2387C | Missense Mutation | Papillary Renal Cell Carcinoma | Unknown |
| K717T | Missense Mutation | Papillary Renal Cell Carcinoma | Unknown |
| T1347I | Missense Mutation | Cutaneous Melanoma | Unknown |
| H1389Y | Missense Mutation | Cutaneous Melanoma | Unknown |
| Q2381H | Missense Mutation | Cutaneous Melanoma | Unknown |
| K2367T | Missense Mutation | Cutaneous Melanoma | Unknown |
| P1577S | Missense Mutation | Cutaneous Melanoma | Unknown |
| R438W | Missense Mutation | Cutaneous Melanoma | Unknown |
| G2307E | Missense Mutation | Cutaneous Melanoma | Unknown |
| C1122F | Missense Mutation | Cutaneous Melanoma | Unknown |
| N997K | Missense Mutation | Cutaneous Melanoma | Unknown |
| T721I | Missense Mutation | Cutaneous Melanoma | Unknown |
| P1514S | Missense Mutation | Cutaneous Melanoma | Unknown |
| P145L | Missense Mutation | Cutaneous Melanoma | Unknown |
| H417Y | Missense Mutation | Cutaneous Melanoma | Unknown |
| S916F | Missense Mutation | Cutaneous Melanoma | Unknown |
| S1694L | Missense Mutation | Cutaneous Melanoma | Unknown |
| C99R | Missense Mutation | Cutaneous Melanoma | Unknown |
| A714S | Missense Mutation | Lung Squamous Cell Carcinoma | Unknown |
| M713I | Missense Mutation | Lung Squamous Cell Carcinoma | Unknown |
| E1329D | Missense Mutation | Uterine Carcinosarcoma/Uterine Malignant Mixed Mullerian Tumor | Unknown |
| F1840Y | Missense Mutation | Head and Neck Squamous Cell Carcinoma | Unknown |
| I2578K | Missense Mutation | Cutaneous Melanoma | Unknown |
| P1891S | Missense Mutation | Cutaneous Melanoma | Unknown |
| F1030I | Missense Mutation | Cutaneous Melanoma | Unknown |
| S1563F | Missense Mutation | Cutaneous Melanoma | Unknown |
| Q1565* | Nonsense Mutation | Melanoma | Unknown |
| S1696F | Missense Mutation | Melanoma | Unknown |
| G55= | Splice Region | Renal Clear Cell Carcinoma | Unknown |
| P410S | Missense Mutation | Cutaneous Melanoma | Unknown |
| R480Vfs*10 | Frame Shift Deletion | Hepatocellular Carcinoma | Unknown |
| X1925_splice | Splice Site | Undifferentiated Pleomorphic Sarcoma/Malignant Fibrous Histiocytoma/High-Grade Spindle Cell Sarcoma | Unknown |
| P145S | Missense Mutation | Lentigo Maligna Melanoma | Unknown |
| D1574Y | Missense Mutation | Non-Seminomatous Germ Cell Tumor | Unknown |
| M713_A714delinsIS | Missense Mutation | Lung Squamous Cell Carcinoma | Unknown |
| H1187N | Missense Mutation | Lung Squamous Cell Carcinoma | Unknown |
| M220I | Missense Mutation | Stomach Adenocarcinoma | Unknown |
| Q1921P | Missense Mutation | Cutaneous Melanoma | Unknown |
| E1674* | Nonsense Mutation | Breast Invasive Lobular Carcinoma | Unknown |
| N1443K | Missense Mutation | Diffuse Large B-Cell Lymphoma | Unknown |
| D250A | Missense Mutation | Diffuse Large B-Cell Lymphoma | Unknown |
| L292P | Missense Mutation | Diffuse Large B-Cell Lymphoma | Unknown |
| A1162T | Missense Mutation | Diffuse Large B-Cell Lymphoma | Unknown |
| Q1565H | Missense Mutation | Diffuse Large B-Cell Lymphoma | Unknown |
| N1179Kfs*10 | Frame Shift Insertion | Renal Clear Cell Carcinoma | Unknown |
| X488_splice | Splice Site | Renal Clear Cell Carcinoma | Unknown |
| K1024T | Missense Mutation | Esophageal Squamous Cell Carcinoma | Unknown |
| Q571E | Missense Mutation | Head and Neck Squamous Cell Carcinoma | Unknown |
| L1057M | Missense Mutation | Lung Adenocarcinoma | Unknown |

| **RAD51 Mutation** | **Mutation Type** | **Cancer Type** | **OncoKB Analysis** |
| --- | --- | --- | --- |
| Q145* | Nonsense Mutation | Serous Ovarian Cancer | Likely Oncogenic |
| R254* | Nonsense Mutation | Pleural Mesothelioma, Epithelioid Type | Likely Oncogenic |
| T297Nfs*26 | Frame Shift Insertion | Hepatocellular Carcinoma | Likely Oncogenic |
| L218R | Missense Mutation | Breast Invasive Ductal Carcinoma | Unknown |
| E176Q | Missense Mutation | Lung Squamous Cell Carcinoma | Unknown |
| A209V | Missense Mutation | Stomach Adenocarcinoma | Unknown |
| S296L | Missense Mutation | Head and Neck Squamous Cell Carcinoma | Unknown |
| A276T | Missense Mutation | Lung Adenocarcinoma | Unknown |
| A194V | Missense Mutation | Uterine Endometrioid Carcinoma | Unknown |
| K57N | Missense Mutation | Uterine Endometrioid Carcinoma | Unknown |
| D37N | Missense Mutation | Bladder Urothelial Carcinoma | Unknown |
| C312Y | Missense Mutation | Bladder Urothelial Carcinoma | Unknown |
| Q206E | Missense Mutation | Bladder Urothelial Carcinoma | Unknown |
| R150Q | Missense Mutation | Renal Clear Cell Carcinoma | Unknown |
| Q272L | Missense Mutation | Renal Clear Cell Carcinoma | Unknown |
| S67I | Missense Mutation | Lung Squamous Cell Carcinoma | Unknown |
| S67I | Missense Mutation | Esophageal Adenocarcinoma | Unknown |
| S67I | Missense Mutation | Lung Squamous Cell Carcinoma | Unknown |
| D12H | Missense Mutation | Lung Adenocarcinoma | Unknown |
| V334L | Missense Mutation | Esophageal Adenocarcinoma | Unknown |
| A175T | Missense Mutation | Esophageal Adenocarcinoma | Unknown |
| H47N | Missense Mutation | Prostate Adenocarcinoma | Unknown |
| F86L | Missense Mutation | Breast Invasive Ductal Carcinoma | Unknown |
| K107N | Missense Mutation | Hepatocellular Carcinoma | Unknown |
| R215K | Missense Mutation | Cutaneous Melanoma | Unknown |
| A246S | Missense Mutation | Non-Seminomatous Germ Cell Tumor | Unknown |
| A192D | Missense Mutation | Adrenocortical Carcinoma | Unknown |
| C312S | Missense Mutation | Lung Squamous Cell Carcinoma | Unknown |

| **RAD51B Mutation** | **Mutation Type** | **Cancer Type** | **OncoKB Analysis** |
| --- | --- | --- | --- |
| E363* | Nonsense Mutation | Uterine Endometrioid Carcinoma | Likely Oncogenic |
| G203* | Nonsense Mutation | Uterine Endometrioid Carcinoma | Likely Oncogenic |
| X151_splice | Splice Site | Lung Squamous Cell Carcinoma | Likely Oncogenic |
| V207L | Missense Mutation | Breast Invasive Lobular Carcinoma | Likely Oncogenic |
| X252_splice | Splice Region | Stomach Adenocarcinoma | Likely Oncogenic |
| Q367* | Nonsense Mutation | Head and Neck Squamous Cell Carcinoma | Likely Oncogenic |
| N130Ifs*9 | Frame Shift Deletion | Hepatocellular Carcinoma | Likely Oncogenic |
| Q319= | Splice Region | Lung Adenocarcinoma | Likely Oncogenic |
| Y244C | Missense Mutation | Stomach Adenocarcinoma | Unknown |
| E37D | Missense Mutation | Uterine Endometrioid Carcinoma | Unknown |
| V177A | Missense Mutation | Uterine Endometrioid Carcinoma | Unknown |
| S3I | Missense Mutation | Lung Squamous Cell Carcinoma | Unknown |
| S3I | Missense Mutation | Lung Squamous Cell Carcinoma | Unknown |
| L52V | Missense Mutation | Lung Squamous Cell Carcinoma | Unknown |
| L52V | Missense Mutation | Lung Squamous Cell Carcinoma | Unknown |
| S403F | Missense Mutation | Undifferentiated Pleomorphic Sarcoma/Malignant Fibrous Histiocytoma/High-Grade Spindle Cell Sarcoma | Unknown |
| D210V | Missense Mutation | Lung Adenocarcinoma | Unknown |
| S287F | Missense Mutation | Lung Squamous Cell Carcinoma | Unknown |
| D272Y | Missense Mutation | Lung Adenocarcinoma | Unknown |
| R58K | Missense Mutation | Lung Squamous Cell Carcinoma | Unknown |
| E51K | Missense Mutation | Lung Squamous Cell Carcinoma | Unknown |
| I251V | Missense Mutation | Breast Invasive Ductal Carcinoma | Unknown |
| K63N | Missense Mutation | Breast Invasive Lobular Carcinoma | Unknown |
| S75P | Missense Mutation | Prostate Adenocarcinoma | Unknown |
| A328S | Missense Mutation | Colon Adenocarcinoma | Unknown |
| K243R | Missense Mutation | Diffuse Large B-Cell Lymphoma | Unknown |
| T260A | Missense Mutation | Hepatocellular Carcinoma | Unknown |
| P109L | Missense Mutation | Cutaneous Melanoma | Unknown |
| L171H | Missense Mutation | Cutaneous Melanoma | Unknown |
| K233T | Missense Mutation | Uterine Carcinosarcoma/Uterine Malignant Mixed Mullerian Tumor | Unknown |
| A222T | Missense Mutation | Prostate Adenocarcinoma | Unknown |
| L31V | Missense Mutation | Esophageal Adenocarcinoma | Unknown |

| **RAD51C Mutation** | **Mutation Type** | **Cancer Type** | **OncoKB Analysis** |
| --- | --- | --- | --- |
| Y224H | Missense Mutation | Bladder Urothelial Carcinoma | Predicted Oncogenic |
| Y224H | Missense Mutation | Bladder Urothelial Carcinoma | Predicted Oncogenic |
| P21S | Missense Mutation | Breast Invasive Ductal Carcinoma | Predicted Oncogenic |
| Q178* | Nonsense Mutation | Lung Adenocarcinoma | Likely Oncogenic |
| Q178* | Nonsense Mutation | Lung Adenocarcinoma | Likely Oncogenic |
| T120Qfs*21 | Frame Shift Deletion | Head and Neck Squamous Cell Carcinoma | Likely Oncogenic |
| G162Efs*9 | Frame Shift Deletion | Lung Adenocarcinoma | Likely Oncogenic |
| G162Efs*9 | Frame Shift Deletion | Lung Adenocarcinoma | Likely Oncogenic |
| X49_splice | Splice Site | Uterine Endometrioid Carcinoma | Likely Oncogenic |
| X49_splice | Splice Site | Lung Squamous Cell Carcinoma | Likely Oncogenic |
| R193Q | Missense Mutation | Uterine Endometrioid Carcinoma | Likely Oncogenic |
| S105Ffs*5 | Frame Shift Insertion | Lung Squamous Cell Carcinoma | Likely Oncogenic |
| X135_splice | Splice Site | Lung Adenocarcinoma | Likely Oncogenic |
| X49_splice | Splice Region | Stomach Adenocarcinoma | Likely Oncogenic |
| X343_splice | Splice Site | Pancreatic Adenocarcinoma | Likely Oncogenic |
| R237* | Nonsense Mutation | Cutaneous Melanoma | Likely Oncogenic |
| E360Q | Missense Mutation | Breast Invasive Ductal Carcinoma | Unknown |
| R249C | Missense Mutation | Cutaneous Melanoma | Unknown |
| V350I | Missense Mutation | Stomach Adenocarcinoma | Unknown |
| P18R | Missense Mutation | Head and Neck Squamous Cell Carcinoma | Unknown |
| K320M | Missense Mutation | Lung Adenocarcinoma | Unknown |
| S231L | Missense Mutation | Lung Adenocarcinoma | Unknown |
| S231L | Missense Mutation | Lung Adenocarcinoma | Unknown |
| N71D | Missense Mutation | Uterine Endometrioid Carcinoma | Unknown |
| A279D | Missense Mutation | Uterine Endometrioid Carcinoma | Unknown |
| L357S | Missense Mutation | Uterine Endometrioid Carcinoma | Unknown |
| R368Q | Missense Mutation | Uterine Endometrioid Carcinoma | Unknown |
| D167N | Missense Mutation | Bladder Urothelial Carcinoma | Unknown |
| D167N | Missense Mutation | Bladder Urothelial Carcinoma | Unknown |
| P330H | Missense Mutation | Bladder Urothelial Carcinoma | Unknown |
| Q340E | Missense Mutation | Glioblastoma Multiforme | Unknown |
| K26N | Missense Mutation | Lung Squamous Cell Carcinoma | Unknown |
| R24L | Missense Mutation | Lung Squamous Cell Carcinoma | Unknown |
| L205F | Missense Mutation | Lung Squamous Cell Carcinoma | Unknown |
| D318E | Missense Mutation | Breast Invasive Ductal Carcinoma | Unknown |
| R312W | Missense Mutation | Colon Adenocarcinoma | Unknown |
| A324T | Missense Mutation | Mucinous Adenocarcinoma of the Colon and Rectum | Unknown |
| S16N | Missense Mutation | Thymoma | Unknown |
| A155V | Missense Mutation | Adrenocortical Carcinoma | Unknown |
| K84M | Missense Mutation | Head and Neck Squamous Cell Carcinoma | Unknown |
| E92K | Missense Mutation | Pancreatic Adenocarcinoma | Unknown |
| G345R | Missense Mutation | Cutaneous Melanoma | Unknown |
| G162R | Missense Mutation | Cutaneous Melanoma | Unknown |
| I63S | Missense Mutation | Lung Adenocarcinoma | Unknown |

| **RAD51D Mutation** | **Mutation Type** | **Cancer Type** | **OncoKB Analysis** |
| --- | --- | --- | --- |
| S257N | Missense Mutation | Uterine Endometrioid Carcinoma | Likely Oncogenic |
| S257N | Missense Mutation | Adrenocortical Carcinoma | Likely Oncogenic |
| Q41* | Nonsense Mutation | Lung Squamous Cell Carcinoma | Likely Oncogenic |
| X49_splice | Splice Site | Stomach Adenocarcinoma | Likely Oncogenic |
| Q115= | Splice Region | Lung Squamous Cell Carcinoma | Likely Oncogenic |
| T154Pfs*40 | Frame Shift Deletion | Hepatocellular Carcinoma | Likely Oncogenic |
| V212I | Missense Mutation | Prostate Adenocarcinoma | Unknown |
| M292I | Missense Mutation | Tubular Stomach Adenocarcinoma | Unknown |
| S88I | Missense Mutation | Head and Neck Squamous Cell Carcinoma | Unknown |
| S298Y | Missense Mutation | Lung Adenocarcinoma | Unknown |
| S298Y | Missense Mutation | Lung Adenocarcinoma | Unknown |
| A59D | Missense Mutation | Uterine Endometrioid Carcinoma | Unknown |
| Q319H | Missense Mutation | Bladder Urothelial Carcinoma | Unknown |
| Q327P | Missense Mutation | Bladder Urothelial Carcinoma | Unknown |
| Q130E | Missense Mutation | Lung Squamous Cell Carcinoma | Unknown |
| Q130E | Missense Mutation | Lung Squamous Cell Carcinoma | Unknown |
| G139V | Missense Mutation | Lung Adenocarcinoma | Unknown |
| L141M | Missense Mutation | Lung Adenocarcinoma | Unknown |
| S62L | Missense Mutation | Lung Squamous Cell Carcinoma | Unknown |
| S33F | Missense Mutation | Breast Invasive Ductal Carcinoma | Unknown |
| R300Q | Missense Mutation | Tubular Stomach Adenocarcinoma | Unknown |
| R145C | Missense Mutation | Mucinous Stomach Adenocarcinoma | Unknown |
| R253Q | Missense Mutation | Rectal Adenocarcinoma | Unknown |
| S213F | Missense Mutation | Breast Invasive Ductal Carcinoma | Unknown |
| K76N | Missense Mutation | Colon Adenocarcinoma | Unknown |
| A296T | Missense Mutation | Colon Adenocarcinoma | Unknown |
| G85C | Missense Mutation | Hepatocellular Carcinoma | Unknown |
| V208D | Missense Mutation | Head and Neck Squamous Cell Carcinoma | Unknown |

| **RAD54L Mutation** | **Mutation Type** | **Cancer Type** | **OncoKB Analysis** |
| --- | --- | --- | --- |
| R52Q | Missense Mutation | Serous Ovarian Cancer | Unknown |
| *748Lext*1 | Nonstop Mutation | Breast Invasive Ductal Carcinoma | Unknown |
| R75Q | Missense Mutation | Uterine Endometrioid Carcinoma | Unknown |
| R154W | Missense Mutation | Pancreatic Adenocarcinoma | Unknown |
| R154W | Missense Mutation | Glioblastoma Multiforme | Unknown |
| R319W | Missense Mutation | Mucinous Adenocarcinoma of the Colon and Rectum | Unknown |
| M442T | Missense Mutation | Breast Invasive Ductal Carcinoma | Unknown |
| R534H | Missense Mutation | Anaplastic Astrocytoma | Unknown |
| R365Q | Missense Mutation | Stomach Adenocarcinoma | Unknown |
| E491K | Missense Mutation | Stomach Adenocarcinoma | Unknown |
| T190M | Missense Mutation | Stomach Adenocarcinoma | Unknown |
| M570V | Missense Mutation | Stomach Adenocarcinoma | Unknown |
| P269T | Missense Mutation | Head and Neck Squamous Cell Carcinoma | Unknown |
| R33M | Missense Mutation | Lung Adenocarcinoma | Unknown |
| K12N | Missense Mutation | Lung Adenocarcinoma | Unknown |
| K12N | Missense Mutation | Lung Adenocarcinoma | Unknown |
| G236A | Missense Mutation | Lung Adenocarcinoma | Unknown |
| G236A | Missense Mutation | Lung Adenocarcinoma | Unknown |
| D366V | Missense Mutation | Lung Adenocarcinoma | Unknown |
| D366V | Missense Mutation | Lung Adenocarcinoma | Unknown |
| R202P | Missense Mutation | Lung Adenocarcinoma | Unknown |
| R202P | Missense Mutation | Lung Adenocarcinoma | Unknown |
| L580P | Missense Mutation | Uterine Endometrioid Carcinoma | Unknown |
| R609* | Nonsense Mutation | Uterine Endometrioid Carcinoma | Unknown |
| V734I | Missense Mutation | Uterine Endometrioid Carcinoma | Unknown |
| L55F | Missense Mutation | Bladder Urothelial Carcinoma | Unknown |
| P15H | Missense Mutation | Uterine Carcinosarcoma/Uterine Malignant Mixed Mullerian Tumor | Unknown |
| P15H | Missense Mutation | Renal Clear Cell Carcinoma | Unknown |
| R395G | Missense Mutation | Lung Squamous Cell Carcinoma | Unknown |
| R395G | Missense Mutation | Lung Squamous Cell Carcinoma | Unknown |
| P483T | Missense Mutation | Lung Adenocarcinoma | Unknown |
| R380W | Missense Mutation | Prostate Adenocarcinoma | Unknown |
| H708Q | Missense Mutation | Breast Invasive Ductal Carcinoma | Unknown |
| Q310R | Missense Mutation | Colon Adenocarcinoma | Unknown |
| I631V | Missense Mutation | Colon Adenocarcinoma | Unknown |
| N707D | Missense Mutation | Serous Ovarian Cancer | Unknown |
| S19A | Missense Mutation | Pancreatic Adenocarcinoma | Unknown |
| F353L | Missense Mutation | Head and Neck Squamous Cell Carcinoma | Unknown |
| N228S | Missense Mutation | Head and Neck Squamous Cell Carcinoma | Unknown |
| K213Tfs*12 | Frame Shift Insertion | Head and Neck Squamous Cell Carcinoma | Unknown |
| P155Lfs*7 | Frame Shift Deletion | Renal Clear Cell Carcinoma | Unknown |
| R537K | Missense Mutation | Lung Adenocarcinoma | Unknown |
| E206Q | Missense Mutation | Lung Squamous Cell Carcinoma | Unknown |
| L400F | Missense Mutation | Lung Squamous Cell Carcinoma | Unknown |
| V216E | Missense Mutation | Lung Adenocarcinoma | Unknown |
| A264T | Missense Mutation | Prostate Adenocarcinoma | Unknown |
| P112S | Missense Mutation | Uterine Carcinosarcoma/Uterine Malignant Mixed Mullerian Tumor | Unknown |
| C391R | Missense Mutation | Esophageal Adenocarcinoma | Unknown |
| S512N | Missense Mutation | Head and Neck Squamous Cell Carcinoma | Unknown |
| X2_splice | Splice Site | Uterine Serous Carcinoma/Uterine Papillary Serous Carcinoma | Unknown |
| S304Y | Missense Mutation | Pancreatic Adenocarcinoma | Unknown |
| L200R | Missense Mutation | Thymoma | Unknown |
| S563 | Splice Region | Uterine Endometrioid Carcinoma | Unknown |
| R511H | Missense Mutation | Pancreatic Adenocarcinoma | Unknown |
| E660D | Missense Mutation | Undifferentiated Pleomorphic Sarcoma/Malignant Fibrous Histiocytoma/High-Grade Spindle Cell Sarcoma | Unknown |
| S337I | Missense Mutation | Intrahepatic Cholangiocarcinoma | Unknown |

| **XRCC3 Mutation** | **Mutation Type** | **Cancer Type** | **OncoKB Analysis** |
| --- | --- | --- | --- |
| A229T | Missense Mutation | Stomach Adenocarcinoma | Unknown |
| S23L | Missense Mutation | Tubular Stomach Adenocarcinoma | Unknown |
| S326Pfs*43 | Frame Shift Deletion | Stomach Adenocarcinoma | Unknown |
| R162H | Missense Mutation | Uterine Endometrioid Carcinoma | Unknown |
| R220H | Missense Mutation | Lung Squamous Cell Carcinoma | Unknown |
| S54Y | Missense Mutation | Lung Squamous Cell Carcinoma | Unknown |
| E222Q | Missense Mutation | Stomach Adenocarcinoma | Unknown |
| S326Lfs*33 | Frame Shift Insertion | Rectal Adenocarcinoma | Unknown |
| I12M | Missense Mutation | Serous Ovarian Cancer | Unknown |
| F175S | Missense Mutation | Uveal Melanoma | Unknown |
| R160Q | Missense Mutation | Pancreatic Adenocarcinoma | Unknown |
| D34E | Missense Mutation | Diffuse Large B-Cell Lymphoma | Unknown |

**Table S3. OncoKB functional analysis of the mutations identified in the TCGA pan cancer cohort (N = 12,153)**

| **Gene A** | **Gene B** | **Neither** | **A Not B** | **B Not A** | **Both** | **P Value** | **Q Value** | **Tendency** |
| --- | --- | --- | --- | --- | --- | --- | --- | --- |
| ATM | FANCM | 8544 | 341 | 188 | 53 | <0.001 | <0.001 | Co-occurrence |
| FANCM | POLQ | 8629 | 194 | 256 | 47 | <0.001 | <0.001 | Co-occurrence |
| BRCA2 | FANCM | 8626 | 259 | 195 | 46 | <0.001 | <0.001 | Co-occurrence |
| ATM | BRCA2 | 8482 | 339 | 250 | 55 | <0.001 | <0.001 | Co-occurrence |
| BRCA2 | BRIP1 | 8715 | 273 | 106 | 32 | <0.001 | <0.001 | Co-occurrence |
| BRCA2 | FANCD2 | 8715 | 274 | 106 | 31 | <0.001 | <0.001 | Co-occurrence |
| FANCE | FANCD2 | 8945 | 44 | 120 | 17 | <0.001 | <0.001 | Co-occurrence |
| ATM | POLQ | 8480 | 343 | 252 | 51 | <0.001 | <0.001 | Co-occurrence |
| POLQ | CDK12 | 8681 | 269 | 142 | 34 | <0.001 | <0.001 | Co-occurrence |
| BRCA2 | CDK12 | 8679 | 271 | 142 | 34 | <0.001 | <0.001 | Co-occurrence |
| BRCA2 | POLQ | 8561 | 262 | 260 | 43 | <0.001 | <0.001 | Co-occurrence |
| BRCA1 | BRCA2 | 8663 | 158 | 271 | 34 | <0.001 | <0.001 | Co-occurrence |
| ATM | BRIP1 | 8626 | 362 | 106 | 32 | <0.001 | <0.001 | Co-occurrence |
| BRCA1 | POLQ | 8664 | 159 | 270 | 33 | <0.001 | <0.001 | Co-occurrence |
| ATM | FANCD2 | 8626 | 363 | 106 | 31 | <0.001 | <0.001 | Co-occurrence |
| BARD1 | FANCM | 8813 | 72 | 220 | 21 | <0.001 | <0.001 | Co-occurrence |
| BRIP1 | POLQ | 8712 | 111 | 276 | 27 | <0.001 | <0.001 | Co-occurrence |
| BRCA1 | BRIP1 | 8818 | 170 | 116 | 22 | <0.001 | <0.001 | Co-occurrence |
| BRCA2 | BARD1 | 8750 | 283 | 71 | 22 | <0.001 | <0.001 | Co-occurrence |
| BRCA2 | FANCE | 8778 | 287 | 43 | 18 | <0.001 | <0.001 | Co-occurrence |
| BRIP1 | FANCD2 | 8869 | 120 | 119 | 18 | <0.001 | <0.001 | Co-occurrence |
| FANCM | CDK12 | 8734 | 216 | 151 | 25 | <0.001 | <0.001 | Co-occurrence |
| ATM | BARD1 | 8662 | 371 | 70 | 23 | <0.001 | <0.001 | Co-occurrence |
| FAN1 | POLQ | 8763 | 60 | 284 | 19 | <0.001 | <0.001 | Co-occurrence |
| BRIP1 | FANCM | 8769 | 116 | 219 | 22 | <0.001 | <0.001 | Co-occurrence |
| BARD1 | PALB2 | 8940 | 80 | 93 | 13 | <0.001 | <0.001 | Co-occurrence |
| BRIP1 | PALB2 | 8897 | 123 | 91 | 15 | <0.001 | <0.001 | Co-occurrence |
| FANCM | FANCD2 | 8769 | 220 | 116 | 21 | <0.001 | <0.001 | Co-occurrence |
| FANCE | CDK12 | 8902 | 48 | 163 | 13 | <0.001 | <0.001 | Co-occurrence |
| BRCA1 | CDK12 | 8779 | 171 | 155 | 21 | <0.001 | <0.001 | Co-occurrence |
| POLQ | FANCD2 | 8709 | 280 | 114 | 23 | <0.001 | <0.001 | Co-occurrence |
| CDK12 | FANCD2 | 8831 | 158 | 119 | 18 | <0.001 | <0.001 | Co-occurrence |
| ATM | BRCA1 | 8571 | 363 | 161 | 31 | <0.001 | <0.001 | Co-occurrence |
| FANCM | RAD51B | 8864 | 230 | 21 | 11 | <0.001 | <0.001 | Co-occurrence |
| ATM | CDK12 | 8585 | 365 | 147 | 29 | <0.001 | <0.001 | Co-occurrence |
| ATM | RAD54L | 8693 | 378 | 39 | 16 | <0.001 | <0.001 | Co-occurrence |
| FANCE | POLQ | 8777 | 46 | 288 | 15 | <0.001 | <0.001 | Co-occurrence |
| BARD1 | POLQ | 8748 | 75 | 285 | 18 | <0.001 | <0.001 | Co-occurrence |
| BARD1 | BRIP1 | 8908 | 80 | 125 | 13 | <0.001 | <0.001 | Co-occurrence |
| BRCA2 | RAD54L | 8780 | 291 | 41 | 14 | <0.001 | <0.001 | Co-occurrence |
| BRCA2 | RAD51B | 8800 | 294 | 21 | 11 | <0.001 | <0.001 | Co-occurrence |
| FANCD2 | DMC1 | 8960 | 128 | 29 | 9 | <0.001 | <0.001 | Co-occurrence |
| ATM | PALB2 | 8647 | 373 | 85 | 21 | <0.001 | <0.001 | Co-occurrence |
| ATM | RAD51B | 8712 | 382 | 20 | 12 | <0.001 | <0.001 | Co-occurrence |
| PALB2 | CDK12 | 8858 | 92 | 162 | 14 | <0.001 | <0.001 | Co-occurrence |
| BRCA1 | FANCD2 | 8813 | 176 | 121 | 16 | <0.001 | <0.001 | Co-occurrence |
| BRCA1 | FANCM | 8714 | 171 | 220 | 21 | <0.001 | <0.001 | Co-occurrence |
| FANCM | DMC1 | 8857 | 231 | 28 | 10 | <0.001 | <0.001 | Co-occurrence |
| BRCA2 | FAN1 | 8757 | 290 | 64 | 15 | <0.001 | <0.001 | Co-occurrence |
| BRCA1 | PALB2 | 8842 | 178 | 92 | 14 | <0.001 | <0.001 | Co-occurrence |
| CHEK2 | FANCL | 8931 | 166 | 21 | 8 | <0.001 | <0.001 | Co-occurrence |
| POLQ | RAD51B | 8801 | 293 | 22 | 10 | <0.001 | <0.001 | Co-occurrence |
| PALB2 | FANCD2 | 8895 | 94 | 125 | 12 | <0.001 | <0.001 | Co-occurrence |
| PALB2 | POLQ | 8734 | 89 | 286 | 17 | <0.001 | <0.001 | Co-occurrence |
| BRCA2 | PALB2 | 8732 | 288 | 89 | 17 | <0.001 | <0.001 | Co-occurrence |
| FANCM | PALB2 | 8794 | 226 | 91 | 15 | <0.001 | <0.001 | Co-occurrence |
| BRIP1 | RAD54L | 8942 | 129 | 46 | 9 | <0.001 | <0.001 | Co-occurrence |
| FAN1 | FANCM | 8819 | 66 | 228 | 13 | <0.001 | <0.001 | Co-occurrence |
| BARD1 | FANCD2 | 8907 | 82 | 126 | 11 | <0.001 | <0.001 | Co-occurrence |
| BRCA2 | RAD51D | 8802 | 296 | 19 | 9 | <0.001 | <0.001 | Co-occurrence |
| POLQ | DMC1 | 8795 | 293 | 28 | 10 | <0.001 | <0.001 | Co-occurrence |
| BRCA2 | DMC1 | 8793 | 295 | 28 | 10 | <0.001 | <0.001 | Co-occurrence |
| BRCA2 | CHEK2 | 8668 | 284 | 153 | 21 | <0.001 | <0.001 | Co-occurrence |
| BRIP1 | CHEK2 | 8828 | 124 | 160 | 14 | <0.001 | <0.001 | Co-occurrence |
| BRIP1 | RAD51B | 8963 | 131 | 25 | 7 | <0.001 | <0.001 | Co-occurrence |
| BRCA1 | BARD1 | 8853 | 180 | 81 | 12 | <0.001 | <0.001 | Co-occurrence |
| POLQ | RAD51C | 8792 | 293 | 31 | 10 | <0.001 | <0.001 | Co-occurrence |
| RAD54L | FANCL | 9047 | 50 | 24 | 5 | <0.001 | <0.001 | Co-occurrence |
| ATM | FAN1 | 8668 | 379 | 64 | 15 | <0.001 | <0.001 | Co-occurrence |
| BARD1 | RAD54L | 8985 | 86 | 48 | 7 | <0.001 | <0.001 | Co-occurrence |
| POLQ | RAD54L | 8779 | 292 | 44 | 11 | <0.001 | <0.001 | Co-occurrence |
| RAD51B | CDK12 | 8925 | 25 | 169 | 7 | <0.001 | <0.001 | Co-occurrence |
| RAD51C | FANCD2 | 8955 | 34 | 130 | 7 | <0.001 | <0.001 | Co-occurrence |
| BRIP1 | RAD51C | 8954 | 131 | 34 | 7 | <0.001 | <0.001 | Co-occurrence |
| BRIP1 | CDK12 | 8825 | 125 | 163 | 13 | <0.001 | <0.001 | Co-occurrence |
| FAN1 | FANCD2 | 8919 | 70 | 128 | 9 | <0.001 | <0.001 | Co-occurrence |
| BARD1 | FANCE | 8979 | 86 | 54 | 7 | <0.001 | <0.001 | Co-occurrence |
| BRIP1 | FAN1 | 8918 | 129 | 70 | 9 | <0.001 | <0.001 | Co-occurrence |
| FANCE | FANCM | 8834 | 51 | 231 | 10 | <0.001 | <0.001 | Co-occurrence |
| ATM | CHEK2 | 8580 | 372 | 152 | 22 | <0.001 | <0.001 | Co-occurrence |
| FANCM | RAD51D | 8864 | 234 | 21 | 7 | <0.001 | <0.001 | Co-occurrence |
| CHEK2 | CDK12 | 8790 | 160 | 162 | 14 | <0.001 | <0.001 | Co-occurrence |
| CDK12 | DMC1 | 8919 | 169 | 31 | 7 | <0.001 | <0.001 | Co-occurrence |
| RAD51B | FANCD2 | 8963 | 26 | 131 | 6 | <0.001 | <0.001 | Co-occurrence |
| FANCE | RAD51C | 9029 | 56 | 36 | 5 | <0.001 | <0.001 | Co-occurrence |
| FAN1 | RAD51B | 9020 | 74 | 27 | 5 | <0.001 | <0.001 | Co-occurrence |
| FANCM | RAD51C | 8852 | 233 | 33 | 8 | <0.001 | <0.001 | Co-occurrence |
| RAD51C | CDK12 | 8916 | 34 | 169 | 7 | <0.001 | <0.001 | Co-occurrence |
| FANCM | RAD54L | 8839 | 232 | 46 | 9 | <0.001 | <0.001 | Co-occurrence |
| BARD1 | FAN1 | 8961 | 86 | 72 | 7 | <0.001 | <0.001 | Co-occurrence |
| ATM | RAD51D | 8712 | 386 | 20 | 8 | <0.001 | <0.001 | Co-occurrence |
| FAN1 | DMC1 | 9014 | 74 | 33 | 5 | <0.001 | <0.001 | Co-occurrence |
| BRCA1 | RAD54L | 8887 | 184 | 47 | 8 | <0.001 | <0.001 | Co-occurrence |
| CHEK2 | FANCE | 8899 | 166 | 53 | 8 | <0.001 | <0.001 | Co-occurrence |
| BRIP1 | DMC1 | 8956 | 132 | 32 | 6 | <0.001 | <0.001 | Co-occurrence |
| RAD51C | DMC1 | 9051 | 37 | 34 | 4 | <0.001 | <0.001 | Co-occurrence |
| ATM | DMC1 | 8703 | 385 | 29 | 9 | <0.001 | <0.001 | Co-occurrence |
| CHEK2 | RAD51B | 8926 | 168 | 26 | 6 | <0.001 | <0.001 | Co-occurrence |
| ATM | RAD51C | 8700 | 385 | 32 | 9 | <0.001 | <0.001 | Co-occurrence |
| ATM | FANCE | 8682 | 383 | 50 | 11 | <0.001 | <0.001 | Co-occurrence |
| CHEK2 | FANCM | 8726 | 159 | 226 | 15 | <0.001 | <0.001 | Co-occurrence |
| BARD1 | RAD51C | 8997 | 88 | 36 | 5 | <0.001 | <0.001 | Co-occurrence |
| CHEK2 | POLQ | 8666 | 157 | 286 | 17 | <0.001 | <0.001 | Co-occurrence |
| BRIP1 | FANCL | 8964 | 133 | 24 | 5 | <0.001 | <0.001 | Co-occurrence |
| BARD1 | CHEK2 | 8868 | 84 | 165 | 9 | <0.001 | <0.001 | Co-occurrence |
| ATM | XRCC3 | 8725 | 389 | 7 | 5 | <0.001 | <0.001 | Co-occurrence |
| FANCM | FANCL | 8862 | 235 | 23 | 6 | <0.001 | <0.001 | Co-occurrence |
| RAD51C | RAD54L | 9034 | 37 | 51 | 4 | <0.001 | <0.001 | Co-occurrence |
| CHEK2 | FAN1 | 8881 | 166 | 71 | 8 | <0.001 | <0.001 | Co-occurrence |
| RAD54L | FANCD2 | 8940 | 49 | 131 | 6 | <0.001 | <0.001 | Co-occurrence |
| FAN1 | FANCE | 8991 | 74 | 56 | 5 | <0.001 | <0.001 | Co-occurrence |
| BRCA1 | RAD51C | 8899 | 186 | 35 | 6 | <0.001 | <0.001 | Co-occurrence |
| RAD51D | DMC1 | 9063 | 25 | 35 | 3 | <0.001 | <0.001 | Co-occurrence |
| BRCA1 | FAN1 | 8863 | 184 | 71 | 8 | <0.001 | <0.001 | Co-occurrence |
| BRCA1 | RAD51D | 8911 | 187 | 23 | 5 | <0.001 | <0.001 | Co-occurrence |
| CHEK2 | FANCD2 | 8825 | 164 | 127 | 10 | <0.001 | <0.001 | Co-occurrence |
| BARD1 | RAD51B | 9005 | 89 | 28 | 4 | <0.001 | <0.001 | Co-occurrence |
| POLQ | FANCL | 8800 | 297 | 23 | 6 | <0.001 | <0.001 | Co-occurrence |
| BRCA2 | FANCL | 8798 | 299 | 23 | 6 | <0.001 | <0.001 | Co-occurrence |
| BRCA2 | RAD51C | 8787 | 298 | 34 | 7 | <0.001 | <0.001 | Co-occurrence |
| BARD1 | CDK12 | 8865 | 85 | 168 | 8 | <0.001 | <0.001 | Co-occurrence |
| FAN1 | RAD51C | 9010 | 75 | 37 | 4 | <0.001 | <0.001 | Co-occurrence |
| PALB2 | RAD54L | 8970 | 101 | 50 | 5 | <0.001 | <0.001 | Co-occurrence |
| BRCA2 | XRCC3 | 8813 | 301 | 8 | 4 | <0.001 | <0.001 | Co-occurrence |
| CHEK2 | DMC1 | 8919 | 169 | 33 | 5 | <0.001 | 0.001 | Co-occurrence |
| FANCD2 | FANCL | 8964 | 133 | 25 | 4 | <0.001 | 0.002 | Co-occurrence |
| BRCA1 | CHEK2 | 8771 | 181 | 163 | 11 | 0.001 | 0.002 | Co-occurrence |
| FANCE | RAD51B | 9036 | 58 | 29 | 3 | 0.001 | 0.002 | Co-occurrence |
| ATM | FANCL | 8709 | 388 | 23 | 6 | 0.001 | 0.002 | Co-occurrence |
| CHEK2 | RAD51D | 8928 | 170 | 24 | 4 | 0.002 | 0.003 | Co-occurrence |
| FANCE | DMC1 | 9030 | 58 | 35 | 3 | 0.002 | 0.004 | Co-occurrence |
| POLQ | RAD51D | 8800 | 298 | 23 | 5 | 0.002 | 0.004 | Co-occurrence |
| BRCA1 | RAD51 | 8910 | 188 | 24 | 4 | 0.003 | 0.005 | Co-occurrence |
| FANCM | XRCC3 | 8876 | 238 | 9 | 3 | 0.003 | 0.006 | Co-occurrence |
| CHEK2 | RAD54L | 8902 | 169 | 50 | 5 | 0.004 | 0.007 | Co-occurrence |
| CDK12 | RAD54L | 8900 | 171 | 50 | 5 | 0.004 | 0.007 | Co-occurrence |
| FAN1 | CDK12 | 8877 | 73 | 170 | 6 | 0.004 | 0.007 | Co-occurrence |
| BRCA1 | RAD51B | 8906 | 188 | 28 | 4 | 0.004 | 0.007 | Co-occurrence |
| FANCE | RAD54L | 9013 | 58 | 52 | 3 | 0.006 | 0.010 | Co-occurrence |
| ATM | RAD51 | 8709 | 389 | 23 | 5 | 0.006 | 0.010 | Co-occurrence |
| POLQ | XRCC3 | 8814 | 300 | 9 | 3 | 0.006 | 0.010 | Co-occurrence |
| FANCL | DMC1 | 9061 | 27 | 36 | 2 | 0.006 | 0.010 | Co-occurrence |
| BARD1 | DMC1 | 8998 | 90 | 35 | 3 | 0.007 | 0.011 | Co-occurrence |
| CHEK2 | RAD51C | 8915 | 170 | 37 | 4 | 0.007 | 0.012 | Co-occurrence |
| RAD51B | DMC1 | 9058 | 30 | 36 | 2 | 0.008 | 0.012 | Co-occurrence |
| BRCA1 | DMC1 | 8900 | 188 | 34 | 4 | 0.008 | 0.013 | Co-occurrence |
| BRIP1 | RAD51D | 8963 | 135 | 25 | 3 | 0.008 | 0.013 | Co-occurrence |
| RAD51B | RAD51C | 9055 | 30 | 39 | 2 | 0.009 | 0.014 | Co-occurrence |
| BRCA1 | FANCE | 8878 | 187 | 56 | 5 | 0.009 | 0.014 | Co-occurrence |
| PALB2 | DMC1 | 8985 | 103 | 35 | 3 | 0.010 | 0.015 | Co-occurrence |
| PALB2 | RAD51C | 8982 | 103 | 38 | 3 | 0.012 | 0.018 | Co-occurrence |
| RAD51D | RAD54L | 9045 | 26 | 53 | 2 | 0.012 | 0.019 | Co-occurrence |
| BRCA2 | FAAP20 | 8809 | 302 | 12 | 3 | 0.012 | 0.019 | Co-occurrence |
| FAAP20 | PALB2 | 9007 | 13 | 104 | 2 | 0.013 | 0.019 | Co-occurrence |
| POLQ | RAD51 | 8799 | 299 | 24 | 4 | 0.013 | 0.019 | Co-occurrence |
| BRIP1 | FANCE | 8931 | 134 | 57 | 4 | 0.013 | 0.020 | Co-occurrence |
| RAD51B | RAD54L | 9041 | 30 | 53 | 2 | 0.016 | 0.023 | Co-occurrence |
| RAD51 | CDK12 | 8925 | 25 | 173 | 3 | 0.016 | 0.024 | Co-occurrence |
| CDK12 | FANCL | 8924 | 173 | 26 | 3 | 0.018 | 0.026 | Co-occurrence |
| FAAP20 | XRCC3 | 9100 | 14 | 11 | 1 | 0.020 | 0.028 | Co-occurrence |
| CDK12 | XRCC3 | 8940 | 174 | 10 | 2 | 0.021 | 0.031 | Co-occurrence |
| BRCA1 | FANCL | 8908 | 189 | 26 | 3 | 0.022 | 0.032 | Co-occurrence |
| ATM | FAAP20 | 8720 | 391 | 12 | 3 | 0.025 | 0.035 | Co-occurrence |
| BRCA1 | XRCC3 | 8924 | 190 | 10 | 2 | 0.025 | 0.036 | Co-occurrence |
| BARD1 | RAD51 | 9007 | 91 | 26 | 2 | 0.033 | 0.046 | Co-occurrence |
| BARD1 | RAD51D | 9007 | 91 | 26 | 2 | 0.033 | 0.046 | Co-occurrence |
| FANCE | PALB2 | 8962 | 58 | 103 | 3 | 0.034 | 0.047 | Co-occurrence |
| BARD1 | FANCL | 9006 | 91 | 27 | 2 | 0.035 | 0.048 | Co-occurrence |
| FANCM | RAD51 | 8860 | 238 | 25 | 3 | 0.037 | 0.050 | Co-occurrence |

**Table S4. Co-occurrence of multiple mutations associated with the recombination pathway in the TCGA pan cancer cohort (N = 12,153)**

| **Gene** | **HR Altered group** | **HR Unaltered group** | **P Value** | **Q Value** |
| --- | --- | --- | --- | --- |
| **TTN** | 1001 (55.12%) | 1682 (23.01%) | 6.81e-148 | 1.33e-143 |
| **MUC16** | 716 (39.43%) | 1045 (14.30%) | 9e-115 | 8.8e-111 |
| **LRP1B** | 525 (28.91%) | 638 (8.73%) | 1.46e-99 | 9.51e-96 |
| **XIRP2** | 403 (22.19%) | 388 (5.31%) | 2.7e-94 | 1.32e-90 |
| **RYR2** | 508 (27.97%) | 651 (8.91%) | 7.88e-90 | 3.08e-86 |
| **USH2A** | 477 (26.27%) | 587 (8.03%) | 8.68e-88 | 2.83e-84 |
| **SYNE1** | 443 (24.39%) | 519 (7.10%) | 1.7e-85 | 4.74e-82 |
| **DNAH9** | 347 (19.11%) | 327 (4.47%) | 1.05e-81 | 2.56e-78 |
| **FLG** | 435 (23.95%) | 522 (7.14%) | 1.82e-81 | 3.95e-78 |
| **ZFHX4** | 419 (23.07%) | 489 (6.69%) | 1.14e-80 | 2.22e-77 |
| **PCDH15** | 340 (18.72%) | 336 (4.60%) | 2.06e-76 | 3.66e-73 |
| **CSMD3** | 423 (23.29%) | 522 (7.14%) | 2.3e-76 | 3.75e-73 |
| **FAT3** | 373 (20.54%) | 409 (5.60%) | 5.26e-76 | 7.92e-73 |
| **CSMD1** | 369 (20.32%) | 417 (5.70%) | 1.09e-72 | 1.52e-69 |
| **DNAH8** | 304 (16.74%) | 285 (3.90%) | 2.97e-71 | 3.87e-68 |
| **ANK3** | 264 (14.54%) | 211 (2.89%) | 1.99e-70 | 2.43e-67 |
| **DNAH7** | 302 (16.63%) | 285 (3.90%) | 2.52e-70 | 2.9e-67 |
| **DNAH5** | 369 (20.32%) | 433 (5.92%) | 1.14e-69 | 1.24e-66 |
| **CACNA1E** | 287 (15.80%) | 263 (3.60%) | 1.88e-68 | 1.94e-65 |
| **MUC17** | 364 (20.04%) | 436 (5.96%) | 5.08e-67 | 4.96e-64 |
| **CDH10** | 254 (13.99%) | 207 (2.83%) | 1.36e-66 | 1.26e-63 |
| **NAV3** | 297 (16.35%) | 294 (4.02%) | 5.02e-66 | 4.46e-63 |
| **ZNF536** | 272 (14.98%) | 245 (3.35%) | 1.06e-65 | 9.03e-63 |
| **ANK2** | 288 (15.86%) | 278 (3.80%) | 1.78e-65 | 1.45e-62 |
| **ASPM** | 239 (13.16%) | 184 (2.52%) | 2.12e-65 | 1.66e-62 |
| **DSCAM** | 244 (13.44%) | 194 (2.65%) | 3.75e-65 | 2.82e-62 |
| **DNAH11** | 277 (15.25%) | 262 (3.58%) | 4.68e-64 | 3.39e-61 |
| **HMCN1** | 329 (18.12%) | 377 (5.16%) | 3.32e-63 | 2.32e-60 |
| **RYR3** | 325 (17.90%) | 369 (5.05%) | 5.02e-63 | 3.38e-60 |
| **MDN1** | 233 (12.83%) | 183 (2.50%) | 1.02e-62 | 6.63e-60 |
| **PCLO** | 388 (21.37%) | 521 (7.13%) | 2.51e-62 | 1.58e-59 |
| **KMT2D** | 325 (17.90%) | 374 (5.12%) | 4.4e-62 | 2.69e-59 |
| **OBSCN** | 325 (17.90%) | 381 (5.21%) | 8.71e-61 | 5.16e-58 |
| **ADAMTS12** | 249 (13.71%) | 222 (3.04%) | 1.86e-60 | 1.05e-57 |
| **CUBN** | 272 (14.98%) | 268 (3.67%) | 1.88e-60 | 1.05e-57 |
| **VPS13D** | 207 (11.40%) | 148 (2.02%) | 1.28e-59 | 6.95e-57 |
| **PAPPA2** | 287 (15.80%) | 304 (4.16%) | 1.72e-59 | 9.06e-57 |
| **APOB** | 329 (18.12%) | 400 (5.47%) | 5.12e-59 | 2.64e-56 |
| **SI** | 282 (15.53%) | 297 (4.06%) | 9.55e-59 | 4.79e-56 |
| **UNC79** | 223 (12.28%) | 180 (2.46%) | 1.17e-58 | 5.61e-56 |
| **NEB** | 286 (15.75%) | 306 (4.19%) | 1.18e-58 | 5.61e-56 |
| **FAT4** | 318 (17.51%) | 377 (5.16%) | 1.28e-58 | 5.96e-56 |
| **SYNE2** | 250 (13.77%) | 236 (3.23%) | 1.19e-57 | 5.41e-55 |
| **FAM135B** | 278 (15.31%) | 294 (4.02%) | 1.27e-57 | 5.64e-55 |
| **LAMA2** | 246 (13.55%) | 229 (3.13%) | 1.98e-57 | 8.6e-55 |
| **SPTA1** | 354 (19.49%) | 471 (6.44%) | 4.71e-57 | 2e-54 |
| **VPS13B** | 232 (12.78%) | 204 (2.79%) | 7.12e-57 | 2.96e-54 |
| **ADGRB3** | 235 (12.94%) | 210 (2.87%) | 7.98e-57 | 3.25e-54 |
| **RELN** | 275 (15.14%) | 295 (4.04%) | 3.95e-56 | 1.58e-53 |
| **MYO18B** | 222 (12.22%) | 189 (2.59%) | 7.06e-56 | 2.76e-53 |

**Table S5. Mutations that co-occur with those associated with the homologous recombination pathway in the TCGA pan cancer cohort (N = 12,153)**

Q = False discovery rate adjusted P value

| **Gene** | **Median Months Survival without Mutation** | **Median Months Survival with Mutation** | **P Value** |  |
| --- | --- | --- | --- | --- |
| **TTN** | 81.11 | 61.47 | P = 5.33e-9 |  |
| **MUC16** | 76.15 | 65.18 | P = 0.0208 |  |
| **LRP1B** | 77.60 | 55.09 | P = 2.008e-6 |  |
| **XIRP2** | 75.69 | 61.24 | P = 0.266 |  |
| **RYR2** | 76.18 | 64.88 | P = 0.0576 |  |
| **USH2A** | 76.58 | 63.59 | P = 0.0376 |  |
| **SYNE1** | 75.53 | 64.72 | P = 0.0904 |  |
| **DNAH9** | 76.15 | 64.52 | P = 0.987 |  |
| **FLG** | 75.53 | 64.24 | P = 0.0857 |  |
| **ZFHX4** | 76.94 | 61.56 | P = 1.728e-3 |  |
| **PCDH15** | 76.30 | 53.61 | P = 1.705e-3 |  |
| **CSMD3** | 76.30 | 56.90 | P = 0.02 |  |
| **FAT3** | 74.67 | 78.61 | P = 0.928 |  |
| **CSMD1** | 75.69 | 63.59 | P = 0.214 |  |
| **DNAH8** | 74.67 | 78.00 | P = 0.754 |  |
| **ANK3** | 74.67 | 78.91 | P = 0.824 |  |
| **DNAH7** | 75.43 | 67.81 | P = 0.284 |  |
| **DNAH5** | 76.30 | 63.93 | P = 0.0622 |  |
| **CACNA1E** | 76.15 | 61.24 | P = 0.204 |  |
| **MUC17** | 74.67 | 76.15 | P = 0.460 |  |
| **CDH10** | 74.97 | 71.50 | P = 0.909 |  |
| **NAV3** | 75.53 | 63.50 | P = 0.178 |  |
| **ZNF536** | 74.97 | 66.43 | P = 0.939 |  |
| **ANK2** | 75.43 | 65.44 | P = 0.0544 |  |
| **ASPM** | 74.87 | 76.18 | P = 0.412 |  |
| **DSCAM** | 73.62 | 102.04 | P = 0.0631 |  |
| **DNAH11** | 75.69 | 64.78 | P = 0.0161 |  |
| **HMCN1** | 76.35 | 61.05 | P = 0.0574 |  |
| **RYR3** | 75.03 | 71.29 | P = 0.489 |  |
| **MDN1** | 75.43 | 61.64 | P = 0.168 |  |
| **PCLO** | 76.18 | 63.70 | P = 0.0407 |  |
| **KMT2D** | 74.97 | 85.45 | P = 0.112 |  |
| **OBSCN** | 75.03 | 61.64 | P = 0.487 |  |
| **ADAMTS12** | 75.53 | 63.50 | P = 0.0129 |  |
| **CUBN** | 75.10 | 62.81 | P = 1.478e-3 |  |
| **VPS13D** | 75.13 | 65.18 | P = 0.375 |  |
| **PAPPA2** | 76.15 | 63.59 | P = 0.0524 |  |
| **APOB** | 75.10 | 66.69 | P = 0.161 |  |
| **SI** | 75.69 | 61.64 | P = 3.257e-3 |  |
| **UNC79** | 74.87 | 76.18 | P = 0.719 |  |
| **NEB** | 75.43 | 64.88 | P = 0.162 |  |
| **FAT4** | 74.87 | 79.24 | P = 0.844 |  |
| **SYNE2** | 75.69 | 60.48 | P = 0.0116 |  |
| **FAM135B** | 75.13 | 62.81 | P = 0.179 |  |
| **LAMA2** | 74.97 | 80.62 | P = 0.635 |  |
| **SPTA1** | 77.04 | 56.44 | P = 0.0234 |  |
| **VPS13B** | 75.43 | 61.47 | P = 0.160 |  |
| **ADGRB3** | 75.10 | 65.18 | P = 0.164 |  |
| **RELN** | 75.53 | 55.23 | P = 0.0799 |  |
| **MYO18B** | 75.10 | 66.69 | P = 0.818 |  |

**Table S6. Prognostic value of mutations that co-occur with those associated with the homologous recombination pathway in the TCGA pan cancer cohort (N = 12,153)**

| Gene | Observed Mutations Diffuse Large B Cell Lymphoma (N=47) | Mutation Frequency (%) |
| --- | --- | --- |
| ATM | 7 | 14.89361702 |
| BARD1 | 1 | 2.127659574 |
| BRCA1 | 2 | 4.255319149 |
| BRAC2 | 1 | 2.127659574 |
| BRCA1/2 | 3 | 6.382978723 |
| BRIP1 | 2 | 4.255319149 |
| CHEK2 | 2 | 4.255319149 |
| CDK12 | 2 | 4.255319149 |
| DMC1 | - | - |
| FAAP20 | - | - |
| FAN1 | 1 | 2.127659574 |
| FANCD2 | 2 | 4.255319149 |
| FANCE | 1 | 2.127659574 |
| FANCL | 1 | 2.127659574 |
| FANCM | 1 | 2.127659574 |
| PALB2 | 2 | 4.255319149 |
| POLQ | 5 | 10.63829787 |
| RAD51 | - | - |
| RAD51B | 1 | 2.127659574 |
| RAD51C | - | - |
| RAD51D | - | - |
| RAD54L | - | - |
| XRCC3 | 1 | 2.127659574 |
| Any HR Mutation | 18 | 38.29787234 |

**Table S7. Mutation frequencies of genes associated with the homologous recombination DNA repair pathway in the TCGA Diffuse Large B Cell Lymphoma cohort (N = 47)**

| Gene | Observed Mutations Cholangiocarcinoma (N=34) | Mutation Frequency (%) |
| --- | --- | --- |
| ATM | 5 | 14.70588235 |
| BARD1 | - | - |
| BRCA1 | 1 | 2.941176471 |
| BRAC2 | 3 | 8.823529412 |
| BRCA1/2 | 4 | 11.76470588 |
| BRIP1 | - | - |
| CHEK2 | 3 | 8.823529412 |
| CDK12 | 2 | 5.882352941 |
| DMC1 | - | - |
| FAAP20 | - | - |
| FAN1 | - | - |
| FANCD2 | 1 | 2.941176471 |
| FANCE | - | - |
| FANCL | - | - |
| FANCM | - | - |
| PALB2 | - | - |
| POLQ | - | - |
| RAD51 | - | - |
| RAD51B | - | - |
| RAD51C | - | - |
| RAD51D | - | - |
| RAD54L | 1 | 2.941176471 |
| XRCC3 | - | - |
| Any HR Mutation | 12 | 33.33333333 |

**Table S8. Mutation frequencies of genes associated with the homologous recombination DNA repair pathway in the TCGA Cholangiocarcinoma cohort (N = 34)**

| Gene | Observed Mutations Uterine Carcinoma (N=57) | Mutation Frequency (%) |
| --- | --- | --- |
| ATM | 2 | 3.50877193 |
| BARD1 | 1 | 1.754385965 |
| BRCA1 | - | - |
| BRAC2 | 3 | 5.263157895 |
| BRCA1/2 | 3 | 5.263157895 |
| BRIP1 | - | - |
| CHEK2 | 4 | 7.01754386 |
| CDK12 | - | - |
| DMC1 | - | - |
| FAAP20 | - | - |
| FAN1 | 1 | 1.754385965 |
| FANCD2 | 1 | 1.754385965 |
| FANCE | 1 | 1.754385965 |
| FANCL | - | - |
| FANCM | 3 | 5.263157895 |
| PALB2 | - | - |
| POLQ | 2 | 3.50877193 |
| RAD51 | - | - |
| RAD51B | 1 | 1.754385965 |
| RAD51C | - | - |
| RAD51D | - | - |
| RAD54L | 2 | 3.50877193 |
| XRCC3 | - | - |
| Any HR Mutation | 12 | 21.05263158 |

**Table S9. Mutation frequencies of genes associated with the homologous recombination DNA repair pathway in the TCGA Uterine carcinoma cohort (N = 57)**
